# Reasons for nonadherence to vaccination for influenza among older people in Brazil

**DOI:** 10.1371/journal.pone.0259640

**Published:** 2021-11-08

**Authors:** Aldiane Gomes de Macedo Bacurau, Ana Paula Sayuri Sato, Priscila Maria Stolses Bergamo Francisco

**Affiliations:** 1 Department of Collective Health, School of Medical Sciences, State University of Campinas, Campinas, São Paulo, Brazil; 2 Department of Epidemiology, School of Public Health, University of São Paulo, São Paulo, Brazil; ISI Foundation: Fondazione ISI - Istituto per l’lnterscambio Scientifico, ITALY

## Abstract

This study aimed to estimate the prevalence of non-vaccination and the reasons for nonadherence to the influenza vaccine among older Brazilians according to sociodemographic characteristics. A cross-sectional study was conducted with data from older people (≥ 60 years of age; n = 23,815) who participated in the 2013 National Health Survey. Frequencies of non-vaccination and the main reasons for nonadherence were calculated with respective 95% confidence intervals. The prevalence of non-vaccination was 26.9% (approximately 7,106,730 older people). The reason *rarely gets the flu* was the most cited among the men (28.2%), the 60-to-69-year-old age group (29.6%), individuals with higher education (41.9%), and those with health insurance (32.3%). *Fear of a reaction* was the most cited reason in the northeastern region (25.4%), among women (29.3%), longer-lived individuals (≥70 years; 28.7%), and those who did not know how to read/write (26.7%). A total of 12.1% reported not believing in the vaccine’s protection, and 5.5% did not know that it was necessary to take vaccine. The proportions of the main reasons for non-vaccination varied by sociodemographic characteristics. This study’s findings highlight the need to increase older people’s knowledge regarding influenza and influenza vaccines. Healthcare providers should be encouraged to counsel older people–especially those in subgroups with lower adherence, such as residents in the Northeast region, those aged 60–69 years, those who do not know how to read/write, those without a spouse/companion, and those without health insurance–regarding the different aspects of the vaccine and formally indicate it for groups at risk.

## Introduction

Influenza is an acute viral respiratory disease of considerable importance to public health that affects 10 to 20% of the world population and causes the death of 290 to 650 thousand people annually [1–3]. It also poses an important challenge in terms of other global health threats, such as chronic diseases [4, 5], as it increases the risk of acute myocardial infarction and stroke and can exacerbate chronic obstructive lung disease (COPD), asthma, diabetes, other diseases, and chronic conditions [2, 3, 5]. Complications and deaths related to influenza are more frequent in high-risk groups, such as older people and individuals with underlying chronic diseases [3, 5].

Vaccination is the most effective way to prevent influenza and is especially important for individuals at high risk of severe forms of the disease [1, 2]. Studies have shown that vaccination is cost effective for high-risk subgroups, including older people [6–8]. In Brazil, the trivalent vaccine composed of the inactivated virus (influenza A/H1N1, A/H3N2, and influenza B) is available free of charge through the public healthcare system to older people and other risk groups [2]. National vaccination campaigns for older people have been conducted since 1999 to reduce the number of complications and deaths related to influenza in this subgroup [2].

The effectiveness of the flu vaccination strategy depends on several factors, including the adherence of the population and the similarity of vaccine composition to circulating strains [6–9]. In Brazil, vaccine coverage among older people fluctuated between 87.3% in 1999 to 91.6% in 2019 [2, 10]. Up to the year 2007, the goal was to vaccinate at least 70% of the target population. Between 2008 and 2016, the goal was 80%, increasing to 90% beginning in 2017 [2, 10]. In 2013, a study identified that the prevalence of influenza vaccination among older people was 73.1% and was also lower than 80% among those with specific chronic diseases [11].

Vaccinated individuals are at a lower risk of developing influenza and similar respiratory conditions [12, 13]. Moreover, vaccination is associated with a reduction in the risk of hospitalization and death not only due to influenza and pneumonia, but also due to cardiovascular disease and is associated with a reduction in the risk of all-cause mortality [14]. Recent studies suggest a possible adjuvant effect of the flu vaccine on the reduction in the severity of Covid-19 (Sars-Cov-2) as well as the mortality rate related to this infection [13, 15]. Studies have shown that vaccination contributes to reducing hospitalizations and deaths due to causes related to influenza in the Brazilian older population [16–18].

Despite the vaccine’s recommendation by the World Health Organization for high-risk groups [1] and the benefits found in vaccinated individuals, nonadherence is common and threatens the reach of the protection necessary for the control of the disease and its complications. Complacency, inconvenience in terms of access, and a lack of trust are considered determinants of non-vaccination [4, 19, 20]. In Brazil, a lack of awareness regarding the benefits of the vaccine, lack of concern with influenza, fear of a reaction, and even the stigma of being considered “elderly” have been the most cited reasons for non-vaccination since the onset of the campaigns [21–23].

Brazil is the sixth most populous country in the world with about 213 million inhabitants [24]. It is experiencing a rapid process of demographic aging in a context of scarce resources and considerable social inequalities [25, 26] among its five geographic regions (North, Northeast, Midwest, Southeast, and South), where the 26 Brazilian states and the Federal District are located. The two most populous cities in the country (São Paulo and Rio de Janeiro) are located in the Southeast region. Demographic and epidemiological changes have not occurred uniformly among Brazilian regions and states, resulting in social and health inequalities and challenges for the national public health care system [27, 28].

Population-based studies conducted with older people in different locations in Brazil have investigated factors associated with vaccination and have indicated some of the reasons for non-vaccination [22, 29–35]. However, no previous study has investigated the distribution of these reasons according to sociodemographic characteristics in a representative sample of the Brazilian older population. Such information could help plan strategies for improving vaccine coverage in different subgroups, as the determinants of nonadherence may be attributed to different sociocultural, political, and personal factors [36, 37].

Therefore, this study aimed to estimate the prevalence of non-vaccination and reasons for nonadherence to the influenza vaccine among Brazilian older people according to sociodemographic characteristics.

## Methods

A cross-sectional study was conducted involving data from older people (≥ 60 years of age; n = 23,815) who participated in the 2013 National Health Survey (in Portuguese: “PNS 2013”), which was a national, home-based survey conducted in 2013 by the Brazilian Institute of Geography and Statistics (IBGE) in partnership with the Health Ministry. The “PNS 2013” collected data on multiple aspects related to the health of the Brazilian population, making it the most comprehensive study on health and its determinants ever conducted in Brazil [38].

To obtain a representative sample of the Brazilian population for the “PNS 2013”, cluster sampling was performed in three stages with the stratification of units. The primary sampling units were formed by census sectors or a set of sectors. The units in the second stage were formed by residences selected by simple random sampling. The unit in the third stage consisted of an adult resident (≥ 18 years) selected with equiprobability in each residence [38].

The survey questionnaire was composed of three parts addressing the household and all residents, which could be answered by a resident with information on the socioeconomic and health status of all residents, and the individual, which was answered exclusively by an adult ≥ 18 years of age selected randomly among all adult residents in the household. Further details on the health survey method, sampling design, and weighting can be found elsewhere [38].

For this study, information was used on the sociodemographic characteristics of the residents (Modules C and D), health insurance (Module I), and health of older people (Module K). Information on vaccination was obtained from the following questions: “*Have you taken the flu vaccine in the last 12 months*?” (yes/no); for those who answered negatively: “*What was the main reason why you did not take the flu vaccine*?”, the “PNS 2013” response categories of which were: *rarely gets the flu*, *did not know taking the flu vaccine was necessary* (recommended), *did not know where to take the vaccine*, *fear of a reaction*, *fear of needles*, *had no accompanier to the health service*, *had financial difficulties*, *had transportation difficulties*, *the health service was distant*, *the vaccine was not available at the service*, *medical contraindication*, *does not believe that the vaccine protects from influenza*, and *other*. In this study, the category “other” consisted of the grouping of the following reasons: *other* (reasons that were not detailed in the “PNS 2013”), *had no accompanier to the health service*, and *had financial difficulties*.

The following sociodemographic variables were considered: region of Brazil (North, Northeast, Central West, South, and Southeast), sex (male or female), age group (60–69, 70–79 or ≥ 80 years), race/skin color (white or black/brown/yellow/indigenous), schooling (no schooling/incomplete primary school, complete primary school/complete high school or incomplete/complete higher education), lives with spouse/companion (yes/no), knows how to read/write (yes/no), and has health insurance (yes/no).

To estimate the absolute number of non-vaccinated older people (≥ 60 years), a variable referring to the population projection provided by the IBGE was used in the analysis command [38]. The point prevalence and prevalence per weighted intervals (95% CI) were calculated according to sociodemographic characteristics and differences between groups (vaccinated and non-vaccinated) were determined using the Rao-Scott chi-square test with the significance level set at 5%. The prevalence rates of the main reasons for non-adherence were also estimated and 95% confidence intervals were considered to compare reasons according to sociodemographic characteristics.

All analyses were performed with the survey module of the Stata 14.0 (*StataCorp LP*, College Station, USA) [39], considering the effects of stratification and clustering in the estimation of indicators and their measures of precision (95% confidence intervals) related to the complex sampling design [38, 39]. We used *svyset* to identify variables for sampling weights and stratification. The technique used for the estimation of variance was linearization (linearized/robust variance estimation). The final weighting consisted of the product of the inverse selection probabilities at each stage of the sampling plan plus the non-response correction processes and calibration adjustments to the known population totals. The command used in the analyses was “svyset upa_pns [pweight = v00281], strata(v0024) vce(linearized) singleunit(certainty)”. The variables mentioned in the command are specific to analysis using the information in the selected resident questionnaire (domicile). Information on the “PNS 2013” sampling plan is available in previous publications [38].

This study was conducted with secondary data in the public domain from the “PNS 2013” available at https://www.ibge.gov.br/en/statistics/social/health/16840-national-survey-of-health.html?=&t=microdados, accessed on August 31, 2020. The survey received approval from the National Human Research Ethics Committee of the Health Ministry (certificate number: 328.159, 26 June 2013).

## Results

The mean age of the older population was 69.9 years (95% CI: 69.7–70.1) and women accounted for the majority of the sample (56.4%; 95% CI: 55.6–57.2). The prevalence of non-vaccination was 26.9% (95% CI: 25.9–28.0). By extrapolating this figure, an estimated 7,106,730 older people (≥ 60 years) were not vaccinated.

In the analysis of non-vaccination according to sociodemographic characteristics, differences were found among the regions of the country, with lower proportions of non-vaccinated older people in the South (22.1%), Central West (22.9%), and Southeast (27.0%) in comparison to the Northeast region (30.6%); p < 0.001. Regarding age, the proportion of non-vaccinated individuals was higher in those aged 60–69 years (28.7%; p < 0.001). Higher proportions of non-vaccination were also found among individuals without a spouse/companion (28.4%; p = 0.010), those did not know how to read/write (29.0%; p = 0.017), and those who did not have health insurance (28.1%; p < 0.001) (Table 1).

**Table 1:** Prevalence of non-vaccination and vaccination for influenza among Brazilian older people according to sociodemographic characteristics. National Health Survey, Brazil, 2013.

*Variable*	N	*Took vaccine for flu in previous 12 months*	
		No	Yes
		% (CI_95%_)	% (CI_95%_)
***Region***		**p-value < 0.001**	
North	4,067	27.6 (24.9–30.4)	72.4 (69.7–75.1)
Northeast	7,373	30.6 (28.7–32.5)	69.4 (67.5–71.3)
Central West	2,658	22.9 (20.8–25.2)	77.1 (74.8–79.2)
Southeast	6,537	27.0 (25.2–28.8)	73.0 (71.2–74.8)
South	3,180	22.1 (20.0–24.4)	77.9 (75.6–80.0)
***Sex***		**p-value = 0.237**	
Male	10,541	27.5 (26.2–28.9)	72.5 (71.1–73.9)
Female	13,274	26.5 (25.2–27.8)	73.5 (72.2–74.8)
***Age group***		**p-value < 0.001**	
60–69 years	13,517	28.7 (27.4–30.0)	71.3 (70.0–72.6)
70–79 years	7,069	24.5 (22.7–26.3)	75.5 (73.7–77.3)
80 year or older	3,229	24.9 (22.4–27.6)	75.1 (72.4–77.6)
***Race/Skin color***		**p-value = 0.300**	
White	11,017	26.4 (25.0–27.9)	73.6 (72.1–75.0)
Black/brown/yellow/indigenous	12,794	27.5 (26.1–29.0)	72.5 (71.0–74.0)
***Lives with spouse/companion***		**p-value = 0.010**	
Yes	13,443	25.8 (24.4–27.2)	74.2 (72.8–75.6)
No	10,372	28.4 (26.9–29.9)	71.6 (70.1–73.1)
***Knows how to read/write***		**p-value = 0.017**	
Yes	17,985	26.3 (25.2–27.5)	73.7 (72.5–74.9)
No	5,830	29.0 (27.1–31.0)	71.0 (69.0–73.0)
***Schooling level***		**p-value = 0.644**	
No schooling/incomplete primary school	16,530	27.1 (25.8–28.3)	72.9 (71.7–74.2)
Complete primary/complete high school	4,926	26.0 (24.0–28.2)	74.0 (71.8–76.0)
Incomplete/complete higher education	2,359	27.7 (24.5–31.1)	72.3 (68.9–75.6)
***Health insurance***		**p-value < 0.001**	
Yes	6,964	24.2 (22.4–26.1)	75.8 (73.9–77.6)
No	16,851	28.1 (26.9–29.4)	71.9 (70.6–73.1)

**Note:** CI_95%_: 95% confidence interval; p-values determined by chi-square test (Rao-Scott).

The reasons for nonadherence to vaccination according to sociodemographic characteristics in the overall sample are displayed in Table 2. The main reasons were *rarely gets the flu* (25.5%) and *fear of a reaction* (25.0%). *Fear of needles* was mentioned by 7.0% of the older people, and 4.1% reported a *medical contraindication*. Moreover, 12.1% reported *not believing that the vaccine protects from the flu*, and 5.5% reported *not knowing that it was necessary to take the vaccine* (Table 2).

**Table 2:** Distribution of reasons for nonadherence of older people to vaccination for influenza according to sociodemographic characteristics. National Health Survey, Brazil, 2013.

*Variables*	Rarely gets flu	Fear of reaction	Other^(a)^	Does not believe vaccine protects from flu	Did not know it was necessary to take vaccine	Fear of needles	Medical contraindication	Vaccine not available at service where it was sought	Health service very distant	Had transportation difficulty	Did not know where to take vaccine
	n = 1.509	n = 1.504	n = 920	n = 704	n = 406	n = 391	n = 257	n = 158	n = 122	n = 111	n = 111
	%	%	%	%	%	%	%	%	%	%	%
	(CI_95%_)	(CI_95%_)	(CI_95%_)	(CI_95%_)	(CI_95%_)	(CI_95%_)	(CI_95%_)	(CI_95%_)	(CI_95%_)	(CI_95%_)	(CI_95%_)
***Total***	**25.5**	**25.0**	**14.3**	**12.1**	**5.5**	**7.0**	**4.1**	**2.5**	**1.2**	**1.6**	**1.3**
	(23.3–27.8)	(23.0–27.1)	**(12.9–15.8)**	**(10.7–13.7)**	**(4.7–6.4)**	**(6.0–8.1)**	**(3.4–4.9)**	**(1.9–3.3)**	(0.9–1.6)	(1.1–2.1)	(1.0–1.8)
***Region***											
North	**19.4**	**21.6**	**16.9**	**8.5**	**8.7**	**9.2**	*	**2.1**	**3.8**	*	**4.9**
	(14.9–29.0)	(17.9–25.7)	(13.5–20.9)	(6.1–11.9)	(5.9–12.6)	(6.0–13.9)		**(1.4–3.2)**	(2.2–6.5)		(2.7–8.7)
Northeast	**19.5**	**25.4**	**16.6**	**12.5**	**5.3**	**8.6**	**3.3**	**3.1**	**1.8**	**2.1**	**1.8**
	**(16.5–22.8)**	**(22.2–29.0)**	(14.1–19.4)	**(10.4–14.8)**	**(4.2–6.7)**	**(6.9–10.6)**	(2.6–4.2)	(1.9–5.0)	(1.0–3.1)	(1.3–3.4)	(1.9–2.8)
Central West	**27.1**	**21.4**	**17.7**	**11.2**	*	*	**4.8**	*	*	*	*
	**(22.8–32.0)**	(17.7–25.6)	(13.9–22.4)	**(8.0–15.4)**			**(3.3–7.2)**				
Southest	**30.5**	**25.4**	**12.4**	**11.9**	**5.6**	**5.9**	**3.9**	*	*	*	*
	**(26.6–34.6)**	**(22.0–29.2)**	(10.3–14.8)	(9.5–14.8)	(4.4–7.3)	(4.5–7.8)	**(2.8–5.4)**				
South	**22.0**	**25.4**	**13.8**	**14.0**	**4.0**	**8.0**	**7.0**	*	*	*	*
	(18.4–26.2)	(21.2–30.1)	(10.5–17.9)	(10.3–18.7)	**(2.5–6.5)**	(5.6–11.3)	(4.9–9.9)				
***Sex***											
Male	**28.2**	**19.7**	**14.2**	**13.6**	**6.3**	**7.8**	**2.2**	**2.8**	**1.5**	**1.9**	**1.8**
	**(25.4–31.1)**	**(17.4–22.1)**	(12.4–16.1)	(11.8–15.7)	**(5.2–7.6)**	**(6.4–9.5)**	(1.6–3.1)	(2.0–4.1)	(1.1–2.1)	(1.3–2.9)	(1.3–2.6)
Female	**23.3**	**29.3**	**14.4**	**10.9**	**4.8**	**6.3**	**5.6**	**2.2**	**0.9**	**1.3**	**1.0**
	**(20.9–25.9)**	**(26.8–31.9)**	**(12.7–16.3)**	**(9.2–12.8)**	(3.9–6.0)	(5.2–7.7)	(4.6–6.9)	**(1.5–3.3)**	(0.6–1.4)	(0.9–1.8)	(0.6–1.5)
***Age group***											
60–69 years	**29.6**	**22.5**	**14.8**	**10.3**	**5.8**	**7.2**	**2.8**	**2.8**	**1.2**	**1.2**	**1.8**
	**(27.1–32.3)**	**(20.1–25.1)**	**(13.1–16.7)**	**(8.8–12.0)**	**(4.9–7.0)**	**(6.0–8.7)**	(2.1–3.7)	(2.0–3.8)	(0.8–1.8)	(0.7–1.8)	(1.3–2.5)
70–79 years	**18.2**	**29.3**	**12.6**	**15.3**	**5.5**	**6.5**	**6.5**	**2.1**	**1.1**	**2.1**	*
	(15.4–21.3)	**(25.7–33.2)**	(10.2–15.4)	(12.6–18.3)	(4.1–7.4)	(4.7–8.9)	(5.0–8.5)	(1.1–3.9)	(0.7–1.9)	(1.3–3.4)	
80 years or older	**21.6**	**27.4**	**15.6**	**13.9**	**3.8**	**6.8**	**5.2**	*	*	*	*
	**(16.7–27.6)**	(22.6–32.8)	(11.9–20.2)	(9.9–19.2)	(2.4–6.0)	(4.5–10.2)	(3.5–7.7)				
***Race/Skin color***											
White	**28.9**	**23.6**	**13.2**	**13.1**	**5.3**	**6.1**	**4.6**	**2.3**	**1.0**	**1.4**	*
	**(25.8–32.2)**	**(21.2–26.3)**	(11.3–15.3)	(10.9–15.7)	(4.2–6.7)	(4.8–7.7)	(3.6–5.8)	**(1.5–3.5)**	(0.6–1.6)	(0.9–2.2)	
Black/brown/yellow/	**21.7**	**26.5**	**15.5**	**11.0**	**5.7**	**8.0**	**3.6**	**2.7**	**1.4**	**1.8**	**2.1**
indigenous	**(19.1–24.5)**	**(23.6–29.6)**	**(13.7–17.6)**	**(9.3–12.9)**	**(4.7–6.9)**	**(6.7–9.6)**	(2.7–4.7)	(1.9–3.9)	(0.9–1.9)	(1.2–2.6)	(1.5–3.0)
***Lives with spouse/companion***											
Yes	**26.2**	**23.1**	**14.9**	**12.3**	**5.4**	**7.4**	**3.8**	**3.1**	**1.4**	**1.2**	**1.2**
	**(23.2–29.3)**	**(20.5–25.9)**	**(12.9–17.2)**	**(10.4–14.5)**	**(4.3–6.6)**	**(6.1–8.9)**	(2.9–4.9)	(2.2–4.6)	(0.9–2.1)	(0.8–2.0)	(0.9–1.8)
No	**24.7**	**27.3**	**13.6**	**11.9**	**5.6**	**6.5**	**4.4**	**1.7**	**0.9**	**1.9**	**1.5**
	(22.1–27.4)	(24.5–30.2)	(11.7–15.7)	(10.1–13.9)	(4.5–7.1)	(5.1–8.2)	**(3.4–5.9)**	(1.1–2.5)	(0.6–1.5)	(1.3–2.9)	(0.9–2.3)
***Knows how to read/write***											
Yes	**27.9**	**24.4**	**14.3**	**12.5**	**5.1**	**6.1**	**4.0**	**2.6**	**0.8**	**1.3**	**1.0**
	**(25.4–30.6)**	**(22.2–26.8)**	(12.7–16.1)	**(10.8–14.5)**	(4.2–6.2)	(5.1–7.3)	**(3.2–4.8)**	**(1.9–3.6)**	(0.5–1.3)	(0.9–2.0)	(0.7–1.4)
No	**17.8**	**26.7**	**14.2**	**10.8**	**6.6**	**9.8**	**4.8**	**2.1**	**2.5**	**2.2**	**2.5**
	**(14.7–21.3)**	**(23.4–30.4)**	**(11.6–17.4)**	(8.8–13.1)	(5.2–8.4)	(7.6–12.5)	**(3.3–6.9)**	(1.2–3.8)	(1.7–3.5)	(1.5–3.4)	(1.6–3.7)
***Schooling level***											
No schooling/incomplete primary school	**21.7**	**26.6**	**13.6**	**12.0**	**6.0**	**8.4**	**4.3**	**2.7**	**1.5**	**1.7**	**1.5**
	**(19.4–24.2)**	**(24.3–29.0)**	(12.0–15.4)	(10.4–13.8)	**(5.0–7.1)**	**(7.2–9.9)**	**(3.5–5.3)**	**(2.0–3.8)**	(1.1–2.1)	(1.3–2.3)	(1.1–2.1)
Complete primary/complete high school	**31.4**	**22.9**	**16.9**	**12.4**	**4.5**	**4.1**	**3.0**	*	*	*	*
	**(27.6–35.5)**	**(19.2–27.1)**	**(13.8–20.4)**	**(9.8–15.6)**	(3.3–6.1)	(2.9–5.9)	(2.1–4.2)				
Incomplete/complete higher education	**41.9**	**17.3**	**14.5**	**12.2**	*	*	**4.9**	*	*	*	*
	**(34.3–50.0)**	(12.0–24.1)	(10.6–19.5)	(8.0–18.2)			**(3.0–7.8)**				
***Health insurance***											
Yes	**32.3**	**22.0**	**16.1**	**12.1**	**4.2**	**4.4**	**4.9**	**2.0**	*	*	*
	**(27.8–37.2)**	**(18.6–25.8)**	**(13.2–19.5)**	**(9.6–15.3)**	(2.9–5.9)	(3.1–6.2)	(3.5–6.6)	**(1.1–3.6)**			
No	**22.9**	**26.1**	**13.6**	**12.1**	**6.0**	**8.0**	**3.8**	**2.7**	**1.4**	**1.6**	**1.8**
	**(20.8–25.1)**	**(23.8–28.6)**	(12.1–15.3)	(10.5–13.9)	**(5.1–7.1)**	**(6.8–9.4)**	**(3.0–4.8)**	**(2.0–3.7)**	(1.0–2.0)	(1.2–2.2)	(1.3–2.3)

Note

* Number of observations (less than 30) insufficient to any estimate with acceptable precision.

**Other**^**(a)**^–Grouping of reasons did not have accompanier to health service, had financial difficulties and other reasons not detailed in National Health Survey.

CI_95%_: 95% confidence interval.

Differences were found among subgroups regarding the main reasons for non-vaccination according to sociodemographic characteristics. *Fear of a reaction* was cited more in the Northeast region of the country (25.4%) and *rarely gets the flu* was cited more in the Central West and Southeast regions (27.1% and 30.5%, respectively). *Rarely gets the flu* was the most common justification for non-vaccination among men (28.2%), whereas *fear of a reaction* was the most common justification for non-vaccination among women (29.3%). *Rarely gets the flu* was the most common justification among self-declared white older people (28.9%), whereas fear of adverse events (26.5%) and non-belief in the protective effect of the vaccine (11.0%) were the most common justifications among self-declared black, brown, yellow, and indigenous individuals. *Rarely gets the flu* was the most common justification among those who knew how to read/write (27.9%) and those who lived with spouse/companion (26.2%), whereas *fear of a reaction* was the most common justification among those who did not know how to read/write (26.7%) and those without spouse/companion (27.3%). *Rarely gets the flu* was the most common justification among those with an incomplete/complete higher education (42%) as well as those with health insurance (32.3%) (Table 2).

**Fig 1:**
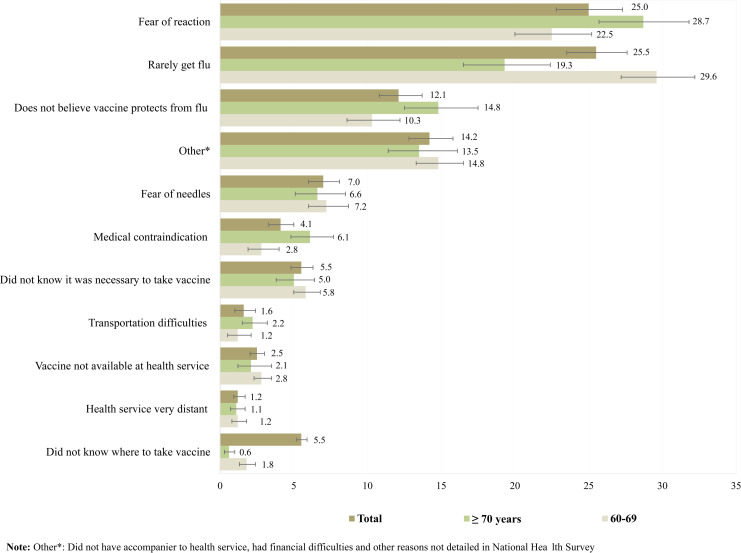
Percentage distribution of main reasons for nonadherence of older people to vaccination for influenza according to age group. National Health Survey, Brazil, 2013.

*Rarely gets the flu* was the most common justification among individuals 60–69 years of age (29.6%), whereas *fear of a reaction* was the most common justification among those ≥ 70 years of age (28.7%). In both age groups, more than 10% reported *not believing that the vaccine protects from the flu* and approximately 5% *did not know it was necessary to take the vaccine*. *Medical contraindication* was reported more by those aged ≥ 70 years (6.1%) (Fig 1).

## Discussion

The present study describes the main reasons for nonadherence to the flu vaccine given by older Brazilians and found that the proportion of these reasons varied according to sociodemographic characteristics. Considering the multiple social situations in different regions of Brazil [28], the findings of this study contribute knowledge on differences in the reasons for non-vaccination among older subgroups. These findings can be useful in the planning of public policies directed at vaccination strategies for overcoming health disparities in line with the needs of the older population.

Studies indicate a reduction in hospitalization and mortality indicators among older people after the start of influenza vaccination campaigns in Brazil [16–18, 40]. Greater adherence to vaccination by older people could contribute to reducing mortality, hospitalizations, and health expenses. Although Brazil is among the countries with the best influenza vaccination coverage in the older population [41–43], the nonadherence of 26.9% and the declared reasons for nonadherence in this group indicate the need to improve the strategies of vaccination campaigns targeting older people. The main reasons for nonadherence (*rarely gets the flu*, *fear of an adverse reaction*, and *not believing that the vaccine protects from influenza*) indicate a lack of concern regarding the disease on the part of older people, fear of adverse events (often mistakenly attributed to the vaccine) and a lack of trust regarding its protective effect. These findings agree with data described in national [34, 35] and international [44–48] studies investigating aspects related to vaccination for influenza in the adult and older populations.

Other investigations conducted in different Brazilian cities (studies with a smaller number of older participants) report not wanting to receive the vaccine [21, 29, 30], believing that it will provoke a reaction [21–23, 31], a lack of counseling from health providers [22], forgetfulness [21, 22], “never getting the flu” [29] and not thinking that the vaccine is necessary [22, 31] as justifications offered by older people for nonadherence. Studies on influenza vaccination for older people, who are particularly vulnerable to negative outcomes from influenza infection, are becoming increasingly relevant due to the rapid growth of this age group in the Brazilian population [24] and the importance of the vaccine as a public health strategy for the prevention of complications resulting from such infection [12, 14].

### Sociodemographic factors and nonadherence to vaccination

A study conducted with data from 11,175 older people of the “PNS 2013” investigating factors associated with vaccination with a focus on socioeconomic differences among Brazil’s regions also identified these reasons as the most frequent justifications for non-vaccination (*fear of side effects*, *rarely get flu*, and *does not believe the vaccine protects against flu*) [35]. However, no previous studies have specified the reasons for nonadherence according to sociodemographic characteristics of the Brazilian older population, which hinders the comparison of our findings. Region of residence alone was considered in one national study [35].

In the study conducted by Andrade *et al*. [35], the highest frequencies of non-vaccinated older people were also in the North and Northeast regions of Brazil and the main reasons for nonadherence were *fear of a reaction* (cited more often in the North, Northeast, and South regions) and *rarely gets the flu* (cited more in the Central West and Southeast regions), which are the same as those in the present study. The different regions of Brazil are marked by differences in demographic density and population aging as well as socio-economic aspects and access to healthcare services [28]. The South and Southeast regions are generally more urban and industrialized, with a larger proportion of older people, better infrastructure, and higher socioeconomic status compared to the North and Northeast (regions with poorer indices of socioeconomic development) [26–28]. Moreover, influenza activity differs among regions. Seasonal influenza activity starts in the equatorial regions of the North and Northeast, extending to areas of tropical and subtropical climate in the South and Southeast, where it reaches in winter [49]. Thus, peak influenza in the North and Northeast regions is believed to occur before the national vaccination campaign, which may exert an impact on the perceptions of older people regarding the effectiveness of the vaccine [16, 49].

In this study, sex was not significantly associated with differences in vaccination prevalence. Other studies involving the older population also found no differences between the sexes and vaccinal status [31, 32, 34, 35, 50, 51]. Regarding the main reasons for nonadherence, *rarely get the flu* was cited more among men (28.2% versus 23.3% among women) and *fear of a reaction* was cited more among women (29.3% versus 19.7% among men). One must bear in mind that perceptions of health, disease, and care may differ between the sexes and that gender patterns established throughout the lives of older people may exert an influence on actions related to health [23]. The stereotype of masculinity, in which men often deny the frailty of illness, can result in the denial of health problems or a tendency to diminish them [52, 53], which may contribute to a lower perception among older men regarding influenza and the need for preventive care.

Several studies have shown that age is associated with the vaccination for influenza [20, 33, 37, 47, 54, 55] and there is a consensus in the literature that individuals between 60–69 years of age adhere less to this prevention measure [22–32, 34, 50, 51]. Sato *et al*. [34] identified lower vaccination coverage in this age group (60–69 years) among Brazilian older people, along with 30% higher odds of vaccination among those age ≥ 70 years (OR = 1.37; 95% CI: 1.17–1.61 for the 70-79-year age group and OR = 1.33; 95% CI: 1.04–1.70 for those ≥ 80 years). A Canadian study [44] involving older people also found that the younger age group was associated with nonadherence. Nonadherence in this younger subgroup (60–69 years) could increase the likelihood of spreading the disease and, consequently, the exposure of individuals aged 70 years or older.

Considering the distribution of the main reasons for nonadherence between age groups, the reason *rarely gets flu* (29.6%) was mentioned more than *fear of reaction* (22.5%) among those aged 60–69 years. *Fear of reaction* was reported more than *rarely gets flu* among those aged 70–79 (29.3% and 18.2%, respectively) as well as those aged 80 and over (27.4% and 21.6%, respectively). The lower perceived risk of getting the flu [20, 37, 47] and the lack of trust in the safety and effectiveness of the vaccine [20, 34, 37] pose a challenge for greater adherence to vaccination. Studies have shown that the perception that the vaccine is not needed [37, 44], the belief that one is not susceptible to influenza [47], and a better self-perception of health status [44] contribute to nonadherence [20, 34, 37, 44, 46–48]. Moreover, self-perceived good health tends to be inversely correlated with age [56], which may favor the recognition of greater vulnerability to the effects of influenza among those aged 70 years or older.

As reported in previous studies [22, 32, 34, 50, 51], no significant association was found between the prevalence of non-vaccination and race/skin color (p = 0.300). This may be partially explained by the Brazilian National Immunization Program’s success, which is based on the public healthcare system’s principles and seeks to ensure free-of-charge, universal access to the vaccine. However, some ethnic groups may have fears and distrust modern medicine and believe that influenza is a natural disease that can be avoided in natural, alternative ways [37], thereby favoring non-vaccination.

In the present study, nonadherence was modest among older people without a spouse/companion (28.4%) compared to those who lived with their spouse/companion (25.8%). Studies have shown an inverse association between vaccination and marital status (single [20, 35, 50, 57] and separated/divorced [35, 51]) and living alone [20, 32, 57]. Thus, social support, access to health services, medical care, and family members’ advice and opinions can stimulate adherence [37, 48]. Older people without company may be less subject to these influences. With the aging of the population, the number of older people living alone tends to increase, which underscores the importance of rethinking vaccination strategies for this subgroup, such as the need for family support regarding the vaccine’s acceptance.

Although this and other studies [32, 34, 50, 51] found no significant association between vaccination and schooling (p = 0.644), some researchers have reported such an association [31, 35, 37, 44, 46, 47, 55]. Jain *et al*. [57] indicate that the effect of schooling is minimized in countries where the vaccine is offered free of charge, whereas adherence is greater among individuals with higher levels of schooling in countries where it is necessary to pay for the vaccine. Higher levels of schooling are positively associated with self-perceived health and income [56, 57] and can lead to better health outcomes, such as adherence to preventive measures, including vaccination. In this study, the reasons for nonadherence differed among individuals with different levels of schooling—*rarely gets the flu* was the most common justification among those with an incomplete/complete higher education (41.9%) and *fear of a reaction* was the most common among those no schooling or with incomplete primary school (26.6%). This suggests that, despite universal coverage, knowledge regarding the vaccine and adverse reactions may be more tenuous among those with a lower level of schooling. Thus, improving health communication can be a strategy for reducing social inequalities in health on the primary care level.

*Fear of a reaction* was the most cited reason for nonadherence among the older people who did not know how to read/write and those with an incomplete primary school education (about 27%). Individuals with a lower level of schooling may have less access to information on the vaccine and are more susceptible to negative beliefs regarding adverse reactions [37]. Bertoldo *et al*. [47] found that in comparison to individuals with a university diploma, those with a lower educational level were less likely to know that influenza is avoidable through the vaccine and that individuals with comorbidities are at greater risk of developing serious influenza complications.

Divergent results have been reported regarding the association between vaccination and having health insurance [34, 35, 57]. A study involving data on Brazilian older people who participated in the “ELSI-Brasil” study found no such association [34], whereas Andrade *et al*. [35] identified a positive association. Sato *et al*. [32] analyzed factors associated with vaccination in 1,341 older residents of São Paulo/SP and found significantly greater coverage among those who had been to healthcare services recently, especially public services.

In Brazil, health insurance companies are not obligated to cover vaccines for older people, whereas the public healthcare system offers such vaccines free of charge [2]. Moreover, having health insurance is more frequent among individuals with a higher level of schooling, which is a *proxy* of income and indicated to be a determinant of greater access to health-related goods and services [58]. This may, at least partially, explain the differences in the distribution of the main reasons reported for non-vaccination, as *fear of a reaction* was mentioned more among older people without health insurance (26.1%) and *rarely get the flu* was mentioned more among those who had insurance (32.3%).

### Other factors related to nonadherence to vaccination

It is noteworthy that vaccines for influenza are generally safe and well-tolerated by older people. The most common side effects are self-limiting and do not result in serious outcomes [6, 12, 59, 60]. The most frequent events are local reactions, such as pain, erythema, swelling. Symptoms similar to those of the flu may also occur, such as malaise, a low fever, respiratory discomfort, cough, and coryza [2, 21, 23–30], which can give a false notion that the vaccine causes the flu. According to the study by Santos *et al*. [48], 74% of individuals in a risk group believed that the vaccine produces symptoms similar to those of the flu.

Being afraid of vaccination and its effects [32] and the myth that the vaccine causes influenza are considerable barriers to adherence [20]. Thus, it is essential for health professionals to explain to the population that the vaccine is composed of the inactivated virus and does not cause the disease as well as clarify the minimum time required to confer protection and the most common types of reactions [2, 23]. Strategies to bolster vaccine confidence must be strengthened, since confidence is related to an increase in vaccination rates among risk groups [32].

The literature reports the lack of belief in the protection offered by the vaccine (reported by more than 10% of the older people in this study) to be a barrier to vaccination [20, 37]. Several factors may be related to the belief that the vaccine does not offer protection, whereas knowledge regarding its safety could increase the likelihood of adherence [37, 47, 48]. A study with Japanese older outpatients [45] found that the frequency of vaccination was greater among individuals previously informed about influenza and belief in the effectiveness and safety of the vaccine was identified as one of the most important reasons for vaccination [55].

Another factor that may negatively contribute to older people’s perceptions regarding the vaccine’s protective effect is that this subgroup may have a lower immune response than young adults [6, 59, 61]. Moreover, the vaccine’s protection may be lower among older people who take medications for chronic conditions [62]. Older people are at greater risk of having complications of influenza and need to be aware that even if vaccinated individuals get influenza, the condition is milder [12]. Moreover, the vaccine’s efficacy is greater if the strains of the vaccine are identical to the circulating strains [60].

About 5% of the older people reported not knowing that the vaccine was necessary. This is higher than the percentage found by Sato *et al*. [34] for older Brazilians and lower than that reported in an international study [45]. Even after more than 20 years of influenza vaccination campaigns for older people in Brazil, aspects related to divulgation and clarification for the population at risk need to be improved.

Medical contraindication was the reason cited by approximately 5% of the respondents. This proportion is close to that found by Sato *et al*. [34]. There are few cases for which the vaccine is contraindicated, such as a severe allergy to some component of the vaccine (anaphylaxis); hives alone after exposure to the egg is not a contraindication and, in cases of moderate or severe acute fever, the recommendation is merely to postpone the vaccination [2].

Considering the importance of the vaccination to reducing morbidity and mortality related to influenza in the older population [12, 16–18, 60], many older people do not adhere to vaccination due to issues that may be the target of interventions. For instance, the lack of a medical recommendation has been reported in the literature as a determinant factor to non-vaccination in this subgroup [20, 47]. The orientation and recommendation of healthcare providers are essential for enhancing knowledge and adherence [37, 45, 55].

Investing in health communication is warranted, with the wide dissemination of clear, correct information about the importance and safety of vaccines. The expansion of “fake news” and the dissemination of news that minimizes the benefits of the vaccine and maximizes possible side effects further underscore the need for correct information. However, the impacts of the political and economic crisis and the austerity measures incorporated in Brazil in recent years, including the approval of Constitutional Amendment No. 95 of 2016, which freezes the federal budget for 20 years [63] (including investments in health), compromises and challenges the Brazilian public health care system and may increase difficulties regarding the development of health promotion and disease prevention/control actions [25, 64, 65].

### Limitations

This study used data from a representative sample of the community-dwelling Brazilian older population and obtained information on reasons for nonadherence to vaccination considering sociodemographic characteristics. The study has limitations should be considered. The cross-sectional design impedes the establishment of causal relations in the associations found. The use of an informant (proxy) in cases for which an older person was unable to answer all or part of the questionnaire constitutes another limitation. Moreover, the survey only considered individuals who resided in private households, excluding institutionalized individuals.

## Conclusions

In conclusion, the proportions of the main reasons given for nonadherence to the vaccination for influenza differed according to sociodemographic characteristics among older people in Brazil. The main three reasons were *rarely gets the flu (25*.*5%)*, *fear of adverse events (25*.*0%)*, and *lack of belief in the vaccine (12*.*1%)*. These findings could assist in establishing more assertive actions focused on specific groups and needs as well as the planning of novel strategies to enhance the participation of older people in vaccination campaigns. As influential, reliable advisers regarding health-related decision-making, healthcare providers should be encouraged to counsel older people–especially those in subgroups with lower adherence, such as residents in the Northeast region, those aged 60–69 years, those who do not know how to read/write, those without a spouse/companion, and those without health insurance–regarding the different aspects of the vaccine and formally indicate it for groups at risk.

## Supporting information

S1 File The survey questionnaire. (PDF)

S2 File Analysis scripts. (PDF)

S3 File Data. (RAR)
