# Correction: Reasons for nonadherence to vaccination for influenza among older people in Brazil

**DOI:** 10.1371/journal.pone.0270443

**Published:** 2022-06-21

**Authors:** Aldiane Gomes de Macedo Bacurau, Ana Paula Sayuri Sato, Priscila Maria Stolses Bergamo Francisco

The first author’s initials appear incorrectly in the citation. The correct citation is: Bacurau AGM, Sato APS, Francisco PMSB (2021) Reasons for nonadherence to vaccination for influenza among older people in Brazil. PLoS ONE 16(11): e0259640. https://doi.org/10.1371/journal.pone.0259640.
