# Supplementary material for: Reasons for nonadherence to vaccination for influenza among older people in Brazil
Source: PLoS One. 2021 Nov 8;16(11):e0259640. doi: 10.1371/journal.pone.0259640 (PMC8575254; doi:10.1371/journal.pone.0259640)
Supplement: S1 File — (PDF) [file pone.0259640.s001.pdf]

IBGE
Instituto Brasileiro de Geografia e Estatística

Diretoria de Pesquisas
Coordenação de Trabalho e Rendimento

PESQUISA NACIONAL DE SAÚDE- 2013

Questionário dos moradores do domicílio

PESQUISA NACIONAL DE SAÚDE

Identificação do Questionário

Unidade da Federação	Município	Distrito	Subdistrito

Bairro	CEP

OBRIGATORIEDADE DE SIGILO DE INFORMAÇÕES - a legislação vigente mantém o caráter obrigatório e confidencial atribuído às informações coletadas pelo IBGE, as quais se destinam, exclusivamente, a fins estatísticos e não poderão ser objeto de certidão e nem terão eficácia jurídica como meio de prova.

Tipo A - Unidade Ocupada

01 **Realizada**
(Quando se realizar a entrevista.)

V0015

02 **Fechada**
(Quando a pesquisa não for realizada na unidade domiciliar devido aos moradores estarem temporariamente ausentes por motivo de férias, viagem etc, durante todo o período de entrevistas.)

03 **Recusa**
(Quando os moradores se recusarem a prestar as informações.)

04 **Outra**
(Quando não houver entrevista na unidade ocupada por motivo que não se enquadre nas duas condições anteriores e que deve ser esclarecido no espaço destinado a observações.)

Tipo B - Unidade Vaga

05 **Em condições de ser habitada**
(Quando a unidade estiver em condições de ser habitada, mas se encontra vaga ou ocupada por pessoas não abrangidas pela pesquisa, como é o caso das unidades de habitação em domicílio coletivo ocupadas exclusivamente por pessoas não moradoras.)

06 **Uso ocasional**
(Quando a unidade for utilizada para descansode fim de semana, férias ou outros fins por pessoas que, presentes ou não no momento da visita do entrevistador são moradoras em outra residência.)

07 **Em construção ou reforma**
(Quando a unidade não estiver ocupada por estar em construção ou reforma.)

08 **Em ruínas**
(Quando a unidade não estiver ocupada por estar em ruínas.)

Tipo C - Unidade Inexistente

09 **Demolida**
(Quando a unidade já foi demolida ou se encontra em fase de demolição.)

10 **Não foi encontrada**
(Quando a unidade houver mudado de lugar (como é o caso de tendas, barracas, reboques etc.) ou não for encontrada por qualquer outro motivo.)

11 **Não residencial**
(Quando a unidade estver sendo utilizada exclusivamente para fins não residenciais.)

12 **Fora do setor**
(Quando, por uma falha, a unidade houver sido listada como pertencente à área (o que tornou possível a sua seleção), embora estivesse situada fora dos seus limites.)

Se Tipo de entrevista = 01, seguir para o módulo A. Caso contrário, encerrar entrevista.

Módulo A - Informações do Domicílio

A1. Este domicílio é do tipo:

A001

1. Casa
2. Apartamento
3. Habitação em casa de cômodos, cortiço ou cabeça-de-porco

(siga A2)

A2. Qual o material que predomina na construção das paredes externas deste domicílio?

A002

1. Alvenaria com revestimento
2. Alvenaria sem revestimento
3. Madeira apropriada para construção
4. Taipa não revestida
5. Madeira aproveitada
6. Palha
7. Outro material (

(siga A3)

A3. Qual o material que predomina na cobertura (telhado) do domicílio?

A003

1. Telha
2. Laje de concreto
3. Madeira apropriada para construção
4. Zinco ou chapa metálica
5. Madeira aproveitada
6. Palha
7. Outro material

(siga A4)

A4. Qual o material que predomina no piso do domicílio?

A004

1. Carpete
2. Cerâmica, lajota ou pedra
3. Tacos ou tábua corrida
4. Cimento
5. Madeira aproveitada
6. Terra
7. Outro material

(siga A5)

A5. Qual é a principal forma de abastecimento de água deste domicílio?

A005

1. Rede geral de distribuição
2. Poço ou nascente na propriedade
3. Poço ou nascente fora da propriedade
4. Carro-pipa
5. Água da chuva armazenada em cisterna
6. Água da chuva armazenada de outro modo
7. Rios, lagos e igarapés
8. Outro material

(Se A5 ≠ 1: passe ao A7.)

A6. Com que frequência a água proveniente da rede geral está habitualmente disponível para este domicílio?(Leia as opções de resposta)

A006

1. Diariamente
2. Pelo menos uma vez por semana
3. Menos que uma vez por semana

(siga A7)

A7. Este domicílio tem água canalizada para pelo menos um cômodo?

A007

1. Sim
2. Não

(siga A8)

A8. Além da principal, que outra forma de abastecimento de água é utilizada neste domicílio?

A008

1. Nenhuma
2. Rede geral de distribuição
3. Poço ou nascente na propriedade
4. Poço ou nascente fora da propriedade
5. Carro-pipa
6. Água da chuva armazenada em cisterna
7. Água da chuva armazenada de outro modo
8. Rios, lagos e igarapés
9. Outra

(siga A9)

A9. A água utilizada para beber neste domicílio é:

A009

1. Filtrada
2. Fervida
3. Tratada de outra forma no domicílio
4. Mineral industrializada
5. Sem tratamento no domicílio

(siga A10)

A10. Qual o número de cômodos no seu domicílio, incluindo banheiro(s) e cozinha(s)?

A010

|__|__| cômodos

(siga A11)

A11. Quantos cômodos estão servindo permanentemente de dormitório para os moradores deste domicílio?

A011 |__|__|

cômodos

(siga A12)

A12. O seu domicílio tem cozinha?

1. Sim
2. Não

A012

(siga A13)

A13. O fogão deste domicílio utiliza predominantemente:

A013

1. Gás de botijão
2. Gás canalizado
3. Lenha
4. Carvão
5. Energia elétrica
6. Outro
7. Não tem fogão

(siga A14)

A14. Quantos banheiros ou sanitários existem neste domicílio?

A014 |__|__| banheiros

0. Nenhum

(Se A14 ≠ 0, siga A15.
Se A14=0, passe ao A16.)

A15. De que forma é feito o escoadouro dos banheiros ou sanitários?

A015

1. Rede geral de esgoto ou pluvial
2. Fossa séptica
3. Fossa rudimentar
4. Vala
5. Direto para rio, lago ou mar
6. Outra

(siga A16)

A16. Qual o destino dado ao lixo?(Leia as opções de resposta)

A016

1. Coletado diretamente por serviço de limpeza
2. Coletado em caçamba de serviço de limpeza
3. É queimado na propriedade
4. É enterrado na propriedade
5. Jogado em terreno baldio ou logradouro
6. Jogado em rio, lago ou mar
7. Outro

(siga A17)

A17. Qual a origem da energia elétrica utilizada neste domicílio?

A017

1. Rede geral
2. Outra origem (gerador, placa solar, eólica etc.)
3. Não tem energia elétrica

(siga A18)

A18. Neste domicílio existe: (Leia as opções de resposta)

a. Televisão em cores? A01801	A01802	☐☐	1. Sim	2. Não
b. Geladeira? A01803	A01804	☐☐	1. Sim	2. Não
c. Vídeo/DVD? A01805	A01806	☐☐	1. Sim	2. Não
d. Máquina de lavar roupa? A01807	A01808	☐☐	1. Sim	2. Não
e. Telefone fixo? A01809	A01810	☐☐	1. Sim	2. Não
f. Telefone celular? A01811	A01812	☐☐	1. Sim	2. Não
g. Forno micro-ondas? A01813	A01814	☐☐	1. Sim	2. Não
h. Computador? A01815	A01816	☐☐	1. Sim	2. Não
i. Motocicleta? A01817	A01818	☐☐	1. Sim	2. Não

(siga A19)

A19. Os moradores têm acesso a internet no domicílio?

A019

1. Sim
2. Não

(siga A20)

A20. Quantos carros tem este domicílio?

A020

☐☐ carros

0. Nenhum

(siga A21)

A21. Em seu domicílio, trabalha algum(a) empregado(a) doméstico(a) mensalista?

A021

1. Sim
2. Não

(siga A22)

A22. Em seu domicílio, há algum cachorro, gato, ave ou peixe?

A022

1. Sim
2. Não

(Se A22 = 2, passe ao módulo B.)

A23. Quantos destes animais há no seu domicílio?(Leia as opções de resposta)

a. gatos	A02301	☐☐	0. Nenhum
b. cachorros	A02302	☐☐	0. Nenhum
c. aves	A02303	☐☐	0. Nenhum
d. peixes	A02304	☐☐	0. Nenhum

(Se A23a ou b>0, siga A24. Se A23a e b=0, encerre a parte.)

A24. Nos últimos 12 meses, os gatos e os cachorros foram vacinados contra raiva?

A024

1. Sim, todos
2. Não, nem todos
3. Nenhum deles

(Encerre o módulo. Passe ao módulo B.)

Módulo B - Visitas domiciliares de Equipe de Saúde da Família e Agentes de Endemias

B1. O seu domicílio está cadastrado na unidade de saúde da família?

B001

1. Sim
2. Não
3. Não sei

(Se B1=2 ou 3, passe ao B4.)

B2. Quando o seu domicílio foi cadastrado?(Leia as opções de resposta)

B002

1. Há menos de 2 meses
2. De 2 a menos de 6 meses
3. De 6 meses a menos de um ano
4. Há um ano ou mais

(siga B3)

B3. Nos últimos 12 meses, com que frequência o seu domicílio recebeu uma visita de algum Agente Comunitário ou algum membro da Equipe de Saúde da Família? (Leia as opções de resposta)

B003

1. Mensalmente
2. A cada 2 meses
3. De 2 a 4 vezes
4. Uma vez
5. Nunca recebeu

(siga B4)

B4. Nos últimos 12 meses, com que frequência o seu domicílio recebeu uma visita de algum agente de endemias (como a dengue, por exemplo)? (Leia as opções de resposta)

B004

1. Mensalmente
2. A cada 2 meses
3. De 2 a 4 vezes
4. Uma vez
5. Nunca recebeu

(Encerre o módulo. Passe ao Módulo C.)

Módulo C - Características gerais dos moradores

C1. Quantas pessoas moram neste domicílio: C001

C3. Número de ordem: C00301

C4. Condição no domicílio: C004

1. Pessoa responsável pelo domicílio
2. Cônjuge ou companheiro(a) de sexo diferente
3. Cônjuge ou companheiro(a) do mesmo sexo
4. Filho(a) do responsável e do cônjuge
5. Filho(a) somente do responsável
6. Enteado(a)
7. Genro ou nora
8. Pai, mãe, padrasto ou madrasta
9. Sogro(a)
10. Neto(a)
11. Bisneto(a)
12. Irmão ou irmã
13. Avô ou avó
14. Outro parente
15. Agregado(a) - Não parente que não compartilha despesas
16. Convivente - Não parente que compartilha despesas
17. Pensionista
18. Empregado(a) doméstico(a)
19. Parente do(a) empregado(a) doméstico(a)

(siga C6)

C6. Sexo: C006

1. Masculino
2. Feminino

(siga C7)

C7. Data de nascimento: C00701 / C00702 / C00703

dia / mês / ano

(siga C8)

C8. Idade: C008

(siga C9)

C9. Cor ou raça:(Leia as opções de resposta) C009

1. Branca
2. Preta
3. Amarela
4. Parda
5. Indígena

(Se C008>=10 anos, siga C10.
Se C008<10, passe ao C12.)

Para moradores de 10 anos ou mais de idade.

C10. _______ vive com cônjuge ou companheiro(a)? C010

1. Sim
2. Não

(siga C11)

C11. Qual o estado civil de ____?(Leia as opções de resposta) C011

1. Casado(a)
2. Separado(a) ou desquitado(a) judicialmente
3. Divorciado(a)
4. Viúvo(a)
5. Solteiro(a)

(siga C12)

Para todos os moradores

C12. O informante desta parte foi: C012

1. A própria pessoa
2. Outro morador
3. Não morador

(Encerre o módulo. Passe ao Módulo D.)

Módulo D - Características de educação das pessoas de 5 anos ou mais de idade

Nesta parte, abordaremos questões sobre a educação de pessoas com 5 anos ou mais de idade.

D1. _______ sabe ler e escrever?

D001

1. Sim
2. Não

(siga D2)

D2. ______ frequenta escola?

D002

1. Sim
2. Não

(Se D2=2, passe ao D8.)

D3. Qual é o curso que _____ frequenta?

D003

1. Pré-escolar (maternal e jardim de infância)
2. Alfabetização de jovens e adultos
3. Regular do ensino fundamental
4. Educação de jovens e adultos (EJA) ou supletivo do ensino fundamental
5. Regular do ensino médio
6. Educação de jovens e adultos (EJA) ou supletivo do ensino médio
7. Superior - graduação
8. Mestrado
9. Doutorado

(Se D3=1, 2, 8 ou 9, passe ao D15. Se D3=3 siga D4. Se D3=4, 5 ou 6, passar ao D5. Se D3=7, passe ao D6.)

D4. A duração deste curso que _____ frequenta é de:

D004

1. 8 anos
2. 9 anos

(siga D5)

D5. Este curso que _____ frequenta é seriado?

D005

1. Sim
2. Não

(Se D5= 2, passe ao D15.)

D6. Se D3 = 3 e D4 = 2 ou Se D3 = 7: Qual é o ano que _____ frequenta?
ou Para os demais casos: Qual é a série que _____ frequenta?

D006

1. Primeira(o)
2. Segunda(o)
3. Terceira(o)
4. Quarta(o)
5. Quinta(o)
6. Sexta(o)
7. Sétima(o)
8. Oitava(o)
9. Nona(o)

(Se D3 = 7 , siga para D7. Caso contrário, passe ao D15.)

D7. _____ já concluiu algum outro curso superior de graduação?

D007

1. Sim
2. Não

(Se D7 = 1, passe ao D13)
(Se D7 = 2, passe ao D15)

D8. Anteriormente _____ frequentou escola?

D008

1. Sim
2. Não

(Se D8=2, passe ao D15.)

D9. Qual foi o curso mais elevado que _____ frequentou anteriormente?

D009

1. Classe de alfabetização – CA
2. Alfabetização de jovens e adultos
3. Antigo primário (elementar)
4. Antigo ginásio (médio 1º ciclo)
5. Regular do ensino fundamental ou do 1º grau
6. Educação de jovens e adultos (EJA) ou supletivo do ensino fundamental
7. Antigo científico, clássico etc. (médio 2º ciclo)
8. Regular do ensino médio ou do 2º grau
9. Educação de jovens e adulto (EJA) ou supletivo do ensino médio
10. Superior - graduação
11. Mestrado
12. Doutorado

(Se D9=1, 2, 11 ou 12, passe ao D14.)
(Se D9=3 ou 10, passe ao D12.)
(Se D9=4, 6, 7, 8 ou 9, passe ao D11.)
(Se D9=5, siga D10.)

D10. A duração deste curso que _____ frequentou anteriormente era de:

D010

1. 8 anos
2. 9 anos

(siga D11)

D11. Este curso que _____ frequentou anteriormente era seriado?

D011

1. Sim
2. Não

(Se D11=2, passe ao D14.)

D12. _____ concluiu, com aprovação, pelo menos a primeira série deste curso que _____ frequentou anteriormente?

D012

1. Sim
2. Não

(Se D12=2, passe ao D15.)

D13. Se D10 = 2 (9 anos): Qual foi o último ano que _____ concluiu, com aprovação, neste curso que frequentou anteriormente? ou
Para os demais casos: Qual foi a última série que _____ concluiu, com aprovação, neste curso que frequentou anteriormente?

D013

1. Primeira(o)
2. Segunda(o)
3. Terceira(o)
4. Quarta(o)
5. Quinta(o)
6. Sexta(o)
7. Sétima(o)
8. Oitava(o)
9. Nona(o)

(siga D14)

D14. ____ concluiu este curso que frequentou anteriormente?

D014

1. Sim
2. Não

(siga D15)

D15. O informante desta parte foi:

D015

1. A própria pessoa
2. Outro morador
3. Não morador

(Encerre o módulo. Passe ao Módulo E.)

Módulo E - Trabalho dos moradores do domicílio

de 14 anos ou mais de idade

Ocupação

E1. Na semana de 21 a 27 de julho de 2013 (semana de referência), ___________ trabalhou ou estagiou, durante pelo menos uma hora, em alguma atividade remunerada em dinheiro?

(Para a pessoa cuja natureza do trabalho implica em ofertar serviços ou aguardar clientes e que esteve à disposição, mas não conseguiu clientes na semana de referência, marque "Sim".)

E001

☐ 1. Sim ☐ 2. Não

(Se E1=1, passe ao E11.)

E2. Na semana de 21 a 27 de julho de 2013 (semana de referência), __________ trabalhou ou estagiou, durante pelo menos uma hora, em alguma atividade remunerada em produtos, mercadorias, moradia, alimentação, experiência profissional, etc.?

E002

☐ 1. Sim ☐ 2. Não

(Se E2=1, passe ao E11.)

E3. Apesar do que acaba de dizer, na semana de 21 a 27 de julho de 2013 (semana de referência), ________________ fez algum bico ou trabalhou em alguma atividade ocasional remunerada durante pelo menos uma hora?

*(**EXEMPLOS**: Na semana de referência a pessoa pode ter preparado doces ou salgados para fora, vendido cosméticos, prestado algum tipo de serviço, etc.)*

E003

☐ 1. Sim ☐ 2. Não

(Se E3=1, passe ao E11.)

E4. Na semana de 21 a 27 de julho de 2013 (semana de referência), __________ ajudou durante pelo menos uma hora, sem receber pagamento, no trabalho remunerado de algum morador do domicílio?

E004

☐ 1. Sim ☐ 2. Não

(Se E4=1, passe ao E11.)

E5. Na semana de 21 a 27 de julho de 2013 (semana de referência), ________________ tinha algum trabalho remunerado do qual estava temporariamente afastado?

*(**ATENÇÃO**: Trabalho remunerado é aquele pelo qual a pessoa recebia dinheiro, produtos, mercadorias ou benefícios, tais como moradia, alimentação, experiência profissional, etc.)*

E005

☐ 1. Sim ☐ 2. Não

(Se E5=2, passe ao E22.)

E6. Na semana de 21 a 27 de julho de 2013 (semana de referência), porque motivo ________________ estava afastado desse trabalho?

E006

☐ 1. Férias, folga ou jornada de trabalho variável
☐ 2. Licença maternidade
☐ 3. Licença remunerada por motivo de doença ou acidente da própria pessoa
☐ 4. Outro tipo de licença remunerada (estudo, paternidade, casamento, licença prêmio etc.)
☐ 5. Afastamento do próprio negócio/empresa por motivo de gestação, doença, acidente etc., sem ser remunerado por instituto de previdência
☐ 6. Fatores ocasionais (tempo, paralisação nos serviços de transporte etc.)
☐ 7. Greve ou paralisação
☐ 8. Outro motivo

(Se 1, 2 ou 6, passe E11. Se 3, passe E8. Se 4, 5 ou 7, passe E10. Se 8, siga E7.)

E7. Durante o tempo de afastamento, __________- continuou a receber ao menos uma parte do pagamento?

E007

☐ 1. Sim ☐ 2. Não

(passe ao E10)

E8. A doença ou acidente foi relacionado ao trabalho?

E008

☐ 1. Sim ☐ 2. Não

(siga E10)

E10. Em 27 de julho de 2013 (último dia da semana de referência), fazia quanto tempo que ___________estava afastado desse trabalho?

E01001 |_|_| dias E01002 |_|_| meses E01003 |_|_| anos

(Se E10 <4 meses e E6 = 3, 4, 5 ou 7, siga para E11.
Se E10 <4 meses e E6 = 8 e E7=1, siga para E11.
Caso contrário, passe E22.)

Pessoas ocupadas

E11. Quantos trabalhos ________________ tinha na semana de 21 a 27 de julho de 2013 (semana de referência)?

E011

☐ 1. Um ☐ 2. Dois ☐ 3. Três ou mais

(Se E11=1, siga para E12. Se E11=2 ou 3, leia o texto abaixo.)

As próximas perguntas são referentes ao trabalho principal.

Critério para definir trabalho principal:

. normalmente trabalhava o maior número de horas.
. recebia normalmente maior rendimento mensal.
. trabalhava há mais tempo, contando até o dia 27 de julho de 2013 (último dia da semana de referência).

Trabalho principal

E12. Qual era a ocupação (cargo ou função) que ________________ tinha nesse trabalho?

E01201

|_|_|_|_| Código |_|_|_|_|_|_|_|_|_|_|_|_|_|_|_|_|_|_|_|_|_|_|_|

(siga E14)

E14. Nesse trabalho, ________________ era:

E014

1. Trabalhador doméstico
2. Militar do exército, da marinha, da aeronáutica, da polícia militar ou do corpo de bombeiros militar
3. Empregado do setor privado
4. Empregado do setor público (inclusive empresas de economia mista)
5. Empregador
6. Conta própria
7. Trabalhador não remunerado em ajuda a conta própria ou empregador que era membro do domicílio
8. Trabalhador não remunerado em ajuda a empregado que era membro do domicílio

(Se E14=1, siga para E16. Caso contrário, siga E15.)

E15. (Se E14=2, 3 ou 4): Qual era a principal atividade desse negócio/empresa do qual ________________ recebia pagamento como empregado?

(Se E14=5 ou 6): Qual era a principal atividade desse negócio/empresa que ________________ tinha?

(Se E14=7 ou 8): Qual era a principal atividade desse negócio/empresa da pessoa do domicílio a quem ________________ ajudava sem receber pagamento?

(***ATENÇÃO****: Anote os principais produtos elaborados ou serviços prestados por esse negócio/empresa.)*

E01501

Código

(Se E14= 7 ou 8, passe ao E17.)

E16. (Se E14= 1, 2, 3 ou 4): Qual era o rendimento bruto mensal que ________________ recebia normalmente nesse trabalho?

(Se E14 = 5 ou 6): Qual era a retirada mensal que ________________ fazia normalmente nesse trabalho?

ATENÇÃO*: O quesito aceita a marcação múltipla para os itens 1 e 2*

1. Valor em dinheiro (R$): E01601 E01602 ,00
2. Valor estimado dos produtos ou mercadorias (R$): E01603 E01604 ,00
3. Somente em benefícios: E01605 (siga E17)

E17. Quantas horas ________________ trabalhava normalmente, por semana, nesse trabalho?

E017 horas

(Se E11 = 1, passe ao E20.
Se E11= 2 ou 3, siga E18.)

Outros trabalhos

E18. Qual era o rendimento bruto mensal ou retirada que ________________ fazia normalmente nesse(s) outro(s) trabalho(s)?

ATENÇÃO*: O quesito aceita a marcação múltipla para os itens 1 e 2*

1. Valor em dinheiro (R$): E01801 E01802 ,00
2. Valor estimado dos produtos ou mercadorias (R$): E01803 E01804 ,00
3. Somente em benefícios: E01805 (siga E19)

E19. Quantas horas ________________ trabalhava normalmente, por semana, nesse(s) outro(s) trabalho(s)?

E019 horas

(siga E20)

Procura de outro trabalho

E20. No período de 28 de junho a 27 de julho de 2013 (período de referência de 30 dias), ________________ tomou alguma providência para conseguir outro trabalho, seja um emprego ou um negócio próprio?

1. Sim
2. Não

E020

(Se E20=2, passe ao E27.)

E21. Qual foi o principal motivo que levou ________________ a procurar outro trabalho no período de 28 de junho a 27 de julho de 2013 (período de referência de 30 dias)?

E021

1. Achava que seria dispensado ou que o negócio/empresa seria fechado
2. Ter um trabalho com garantias trabalhistas ou com maior estabilidade
3. Ter o seu próprio negócio/empresa
4. Conseguir um trabalho de acordo com a sua experiência profissional e/ou formação
5. Melhorar suas condições de bem-estar (menos desgaste físico, melhores relações de trabalho, menos tempo de transporte etc .)
6. Aumentar os rendimentos
7. Trabalhar menor número de horas, mesmo com redução de rendimentos
8. Outro motivo

(passe ao E27)

Pessoas não ocupadas - Procura de trabalho

E22. No período de 28 de junho a 27 de julho de 2013 (período de referência de 30 dias), ________________ tomou alguma providência para conseguir trabalho, seja um emprego ou um negócio próprio?

E022

- 1. Sim
- 2. Não

(Se E22=2, passe ao E24.)

E23. No período de 28 de junho a 27 de julho de 2013 (período de referência de 30 dias), qual foi a principal providência que ________________ tomou para conseguir trabalho?

E023

- 1. Entrou diretamente em contato com empregador (em fábrica, fazenda, mercado, loja ou outro local de trabalho)
- 2. Fez ou inscreveu-se em concurso
- 3. Consultou agência privada ou sindicato
- 4. Consultou agência municipal, estadual ou o Sistema Nacional de Emprego (SINE)
- 5. Colocou ou respondeu anúncio
- 6. Consultou parente, amigo ou colega
- 7. Buscou ajuda financeira para iniciar o próprio negócio
- 8. Procurou local, equipamento ou maquinário para iniciar o próprio negócio
- 9. Solicitou registro ou licença para iniciar o próprio negócio
- 10. Tomou outra providência
- 11. Não tomou providência efetiva

(Se E23≠ 11, passe ao E25.)

E24. Qual foi o principal motivo de ________________ não ter tomado providência para conseguir trabalho no período de 28 de junho a 27 de julho de 2013 (período de referência de 30 dias)?

E024

- 1. Conseguiu proposta de trabalho para começar após a semana de referência
- 2. Aguardando resposta de medida tomada para conseguir trabalho
- 3. Desistiu de procurar por não conseguir encontrar trabalho
- 4. Acha que não vai encontrar trabalho por ser muito jovem ou muito idoso
- 5. Tinha que cuidar de filho(s), de outro(s) dependente(s) ou dos afazeres domésticos
- 6. Estudo
- 7. Incapacidade física, mental ou doença permanente
- 8. Aposentado por idade/tempo de serviço ou contribuição
- 9. Aposentado por doença/invalidez
- 10. Não desejava trabalhar
- 11. Outro motivo

(Se E24=1 ou 2, siga E25. Se E24=3, 4, 5, 6, 7, 8, 9, 10 ou 11, passe ao E26.)

E25. Até o dia 27 de julho de 2013 (último dia da semana de referência), fazia quanto tempo que ________________ estava sem qualquer trabalho e tentando conseguir trabalho?

ATENÇÃO*: O tempo de procura deve ser contínuo. Se a pessoa teve qualquer trabalho ou parou de procurar por 2 semanas ou mais, comece a contar a partir da data que reiniciou a procura.*

E025

- 1. Menos de 1 mês
- 2. De 1 mês a menos de 1 ano (|_|_| meses) E02501
- 3. De 1 ano a menos de 2 anos (1 ano e |_|_| meses) E02502
- 4. 2 anos ou mais (|_|_| anos) E02503

(siga E26)

E26. Se tivesse conseguido um trabalho ________________ poderia ter começado a trabalhar na semana de 21 a 27 de julho de 2013 (semana de referência)?

E026

- 1. Sim
- 2. Não

(siga E27)

E27. O informante desta parte foi:

E027

- 1. A própria pessoa
- 2. Outro morador |_|_|
- 3. Não morador

(Encerre o módulo. Passe ao Módulo F)

Módulo F - Rendimentos domiciliares

No mês de julho (mês de referência) algum morador desse domicílio recebeu rendimento de:

F1. Aposentadoria ou pensão de instituto de previdência federal (INSS), estadual, municipal ou do governo federal, estadual, municipal?

F001

- 1. Sim
  - 1. Morador 1 (R$ ______________) F00102
  - 2. Morador 2 (R$ ______________)
  - 3. Morador 3 (R$ ______________)
  - n. Morador n (R$ ______________)
- 2. Não

(siga F7)

F7. Pensão alimentícia ou doação em dinheiro de pessoa que não morava no domicílio?

F007

- 1. Sim
  - 1. Morador 1 (R$ ______________) F00702
  - 2. Morador 2 (R$ ______________)
  - 3. Morador 3 (R$ ______________)
  - n. Morador n (R$ ______________)
- 2. Não

(siga F8)

F8. Aluguel ou arrendamento?

1. Sim F008 — 1. Morador 1 (R$ ______________) F00802
2. Morador 2 (R$ ______________)
3. Morador 3 (R$ ______________)
n. Morador n (R$ ______________)

2. Não

(siga F10)

F10. Seguro-desemprego, seguro defeso?

1. Sim F010 — 1. Morador 1 (R$ ______________) F01002
2. Morador 2 (R$ ______________)
3. Morador 3 (R$ ______________)
n. Morador n (R$ ______________)

2. Não

(siga F11)

F11. Benefício Assistencial de Prestação Continuada BPC-LOAS?

1. Sim F011 — 1. Morador 1 (R$ ______________) F01102
2. Morador 2 (R$ ______________)
3. Morador 3 (R$ ______________)
n. Morador n (R$ ______________)

2. Não

(siga F12)

F12. Programa Bolsa Família?

1. Sim F012 — 1. Morador 1 (R$ ______________) F01202
2. Morador 2 (R$ ______________)
3. Morador 3 (R$ ______________)
n. Morador n (R$ ______________)

2. Não

(siga F13)

F13. Outros programas sociais do governo?

1. Sim F013 — 1. Morador 1 (R$ ______________) F01302
2. Morador 2 (R$ ______________)
3. Morador 3 (R$ ______________)
n. Morador n (R$ ______________)

2. Não

(siga F14)

F14. Rendimentos de caderneta de poupança, juros de aplicação financeira ou dividendos?

1. Sim F014 — 1. Morador 1 (R$ ______________) F01402
2. Morador 2 (R$ ______________)
3. Morador 3 (R$ ______________)
n. Morador n (R$ ______________)

2. Não

(siga F15)

F15. Outros rendimentos?

1. Sim F015 — 1. Morador 1 (R$ ______________) F01502
2. Morador 2 (R$ ______________)
3. Morador 3 (R$ ______________)
n. Morador n (R$ ______________)

2. Não

(Encerre o módulo. Passe ao Módulo G)

Módulo G - Pessoas com Deficiências

Neste módulo, abordaremos questões sobre deficiências. Primeiramente, vamos abordar a deficiência intelectual, isto é, desenvolvimento intelectual abaixo do normal.

G1. _______tem deficiência intelectual?

G001

1. Sim
2. Não

(Se G1=2, passe ao G6.)

G2. _________nasceu com a deficiência intelectual ou a deficiência foi adquirida por doença ou acidente?

G002

1. Nasceu com a deficiência
2. Foi adquirida . Com que idade? G00201 |__|__|

(siga G3)

G3. A deficiência intelectual está associada a alguma dessas síndromes ou transtornos de desenvolvimento?(Leia as opções de resposta)

G003

1. Síndrome de Down
2. Autismo
3. Paralisia cerebral
4. Outra síndrome
5. AVC, AVE, derrame. aneurisma ou epilepsia
6. Demência séria ou falhas de memória

(siga G4)

G4. Em geral, em que grau a deficiência intelectual limita as atividades habituais (como ir à escola, brincar, trabalhar etc.) de _______________?(Leia as opções de resposta)

G004

- 1. Não limita
- 2. Um pouco
- 3. Moderadamente
- 4. Intensamente
- 5. Muito intensamente/Não consegue

(siga G5)

G5. _________frequenta algum serviço de reabilitação devido à deficiência intelectual?

G005

- 1. Sim
- 2. Não

(siga G6)

G6. _________tem alguma deficiência física?

G006

- 1. Sim
- 2. Não

(Se G6=2, passe ao G14.)

G7. _________nasceu com a deficiência física ou a deficiência foi adquirida por doença ou acidente?

G007

- 1. Nasceu com a deficiência
- 2. Foi adquirida. Com que idade? G00701

(siga G8)

G8. Qual deficiência física?

G008

- 01. Paralisia permanente de um dos lados do corpo
- 02. Paralisia permanente das pernas e dos braços
- 03. Paralisia permanente das pernas
- 04. Paralisia permanente de uma das pernas
- 05. Amputação ou ausência de perna
- 06. Amputação ou ausência de braço
- 07. Amputação ou ausência de mão
- 08. Amputação ou ausência de pé
- 09. Deformidade congênita ou adquirida em um ou mais membros
- 10. Deficiência motora em decorrência de poliomielite ou paralisia infantil
- 11. Ostomia (adaptação de bolsa de fezes e/ou urina)
- 12. Nanismo
- 13. Outra
- 14. Amputação de dedos

(siga G9)

G9. Em geral, em que grau a deficiência física limita as atividades habituais de __________ ?(Leia as opções de resposta)

G009

- 1. Não limita
- 2. Um pouco
- 3. Moderadamente
- 4. Intensamente
- 5. Muito intensamente/ Não consegue

(siga G10)

G10. _________frequenta algum serviço de reabilitação devido à deficiência física?

G010

- 1. Sim
- 2. Não

(siga G14)

Agora vamos abordar a deficiência auditiva permanente, isto é, perda parcial ou total das possibilidades de ouvir.

G14. _________tem deficiência auditiva?

G014

- 1. Sim
- 2. Não

(Se G14=2, passe ao G21.)

G15. _________nasceu com a deficiência auditiva ou a deficiência foi adquirida?

G015

- 1. Nasceu com a deficiência
- 2. Foi adquirida. Com que idade? G01501

(siga G16)

G16. Qual deficiência auditiva?

G016

- 1. Surdez dos dois ouvidos
- 2. Surdez de um ouvido e audição reduzida do outro
- 3. Surdez de um ouvido e audição normal do outro
- 4. Audição reduzida de ambos os ouvidos
- 5. Audição reduzida em um dos ouvidos

(siga G17)

G17. Em geral, em que grau a deficiência auditiva limita as atividades habituais de _____________?(Leia as opções de resposta)

G017

- 1. Não limita
- 2. Um pouco
- 3. Moderadamente
- 4. Intensamente
- 5. Muito intensamente/ Não consegue

(siga G18)

G18. _________frequenta algum serviço de reabilitação devido à deficiência auditiva?

G018

- 1. Sim
- 2. Não

(siga G21)

Agora vamos abordar a deficiência visual permanente, isto é, perda parcial ou total das possibilidades de ver.

G21. ____ tem deficiência visual?

G021

- 1. Sim
- 2. Não

(Se G21=2, passe ao G32.)

G22. _________nasceu com a deficiência visual ou a deficiência foi adquirida?

G022

- 1. Nasceu com a deficiência
- 2. Foi adquirida. Com que idade? G02201

(siga G23)

G23. Qual deficiência visual?

G023

- 1. Cegueira de ambos os olhos
- 2. Cegueira de um olho e visão reduzida do outro
- 3. Cegueira de um olho e visão normal do outro
- 4. Baixa visão de ambos os olhos
- 5. Baixa visão em um dos olhos

(siga G24)

G24. _____ usa algum recurso para auxiliar a locomoção?

- 1. Sim
- 2. Não

(Se G24=1, siga G25.
Se G24=2, passe ao G26.)

G024

G25. Qual ou quais destes recursos _____ faz uso? (Leia as opções de resposta)

a. Bengala articulada	G02501	1. Sim	2. Não
b. Cão guia	G02502	1. Sim	2. Não
c. Outro	G02503	1. Sim	2. Não

(siga G26)

G26. Em geral, em que grau a deficiência visual limita as atividades habituais de __________________________________?(Leia as opções de resposta)

1. Não limita
2. Um pouco
3. Moderadamente
4. Intensamente
5. Muito intensamente

G026

(siga G27)

G27. _________frequenta algum serviço de reabilitação devido à deficiência visual?

1. Sim
2. Não

G027 (siga G32)

G32. O informante desta parte foi:

1. A própria pessoa
2. Outro morador
3. Não morador

G032

(Encerre o módulo. Passe ao Módulo I)

Módulo I - Cobertura de Plano de Saúde

Agora gostaria de lhe fazer algumas perguntas sobre plano ou seguro de saúde.

I1. _____ tem algum plano de saúde (médico ou odontológico), particular, de empresa ou órgão público?

1. Sim
2. Não

I001

(Se I1=2, passe ao I012.)

I2. _____ tem quantos planos de saúde (médico ou odontológico) particular, de empresa ou órgão público?

I002

(siga I3)

I3. _____ tem algum plano de saúde apenas para assistência odontológica?

1. Sim
2. Não

I003

(Se I2=1, siga I4. Se I2>1, leia o texto: Para as questões seguintes, considere o plano de saúde principal.)

I4. O plano de saúde (**único** ou **principal**) que _____ possui é de instituição de assistência de servidor público (municipal, estadual ou militar)?

1. Sim
2. Não

I004

(siga I5)

I5. Há quanto tempo sem interrupção _____ possui esse plano de saúde?(Leia as opções de resposta)

1. Até 6 meses
2. Mais de 6 meses até 1 ano
3. Mais de 1 ano até 2 anos
4. Mais de 2 anos

I005

(siga I006)

I6. _____ considera este plano de saúde:

1. Muito bom
2. Bom
3. Regular
4. Ruim
5. Muito ruim
6. Nunca usou o plano de saúde

I006

(siga I7)

I7. Quem é o titular do plano de saúde de ______?

I007

1. Número de ordem do titular
2. Titular não morador

I00701

(I7=2, passe ao I12.)

I8. _____ tem alguém que não mora neste domicílio como dependente ou agregado neste plano de saúde?

1. Sim
2. Não

I008

(Se I8=1, siga I9. Se I8=2, passe ao I10.)

I9. Quantas pessoas que não moram neste domicílio ______ tem como dependentes ou agregados no plano de saúde?

I009

(siga I10)

I10. Quem paga a mensalidade deste plano de saúde?

1. Somente o empregador do titular
2. O titular, através do trabalho atual
3. O titular, através do trabalho anterior
4. O titular, diretamente ao plano
5. Outro morador do domicílio
6. Pessoa não moradora do domicílio
7. Outro

I010

(Se I10=2, 3, 4 ou 5, siga I11. Se I10=1, 6 ou 7, passe ao I12.)

I11. Qual é o valor da mensalidade deste plano de saúde?(Leia as opções de resposta)

1. Menos de R$50,00
2. De R$50,00 a menos de R$100,00
3. De R$100,00 a menos de R$200,00
4. De R$200,00 a menos de R$300,00
5. De R$300,00 a menos de R$500,00
6. De R$500,00 a menos de R$1000,00
7. R$1000,00 e mais

I011

(Encerre o módulo. Passe ao módulo J.)

Módulo J - Utilização de Serviços de Saúde

Agora vou lhe fazer perguntas sobre o estado de saúde e utilização de serviços de saúde dos moradores do domicílio.

J1. De um modo geral, como é o estado de saúde de _________? (Leia as opções de resposta)

1. Muito bom
2. Bom
3. Regular
4. Ruim
5. Muito ruim

J001

(siga J2)

J2. Nas duas últimas semanas, _____ deixou de realizar quaisquer de suas atividades habituais (trabalhar, ir à escola, brincar, afazeres domésticos etc.) por motivo de saúde?

1. Sim
2. Não

J002

(Se J2=2, passe ao J7.)

J3. Nas duas últimas semanas, quantos dias _____ deixou de realizar suas atividades habituais, por motivo de saúde?

J003

dias

(siga J4)

J4. Qual foi o principal motivo de saúde que impediu _____ de realizar suas atividade habituais nas duas últimas semanas?

J004

- 01. Dor nas costas, problema no pescoço ou na nuca
- 02. Dor nos braços ou nas mãos
- 03. Artrite ou reumatismo
- 04. DORT- doença osteomuscular relacionada ao trabalho
- 05. Dor de cabeça ou enxaqueca
- 06. Problemas menstruais
- 07. Problemas da gravidez
- 08. Parto
- 09. Problema odontológico
- 10. Resfriado / gripe
- 11. Asma / bronquite / pneumonia
- 12. Diarréia / vômito / náusea / gastrite
- 13. Dengue
- 14. Pressão alta ou outra doença do coração (como infarto, angina, insuficiência cardíaca)
- 15. Diabetes
- 16. AVC ou derrame
- 17. Câncer
- 18. Depressão
- 19. Outro problema de saúde mental
- 20. Outra doença
- 21. Lesão provocada por acidente de trânsito
- 22. Lesão provocada por outro tipo de acidente
- 23. Lesão provocada por agressão ou outra violência
- 24. Outro problema de saúde

(siga J5)

J5. Nas duas últimas semanas _____ esteve acamado(a)?

J005

- 1. Sim
- 2. Não

(Se J5=2, passe ao J7.)

J6. Nas duas últimas semanas, quantos dias _____ esteve acamado(a)?

J006

dias

(siga J7)

J7. Algum médico já deu o diagnóstico de alguma doença crônica, física ou mental, ou doença de longa duração (de mais de 6 meses de duração) a ___________?

J007

- 1. Sim
- 2. Não

(Se J7=2, passe ao J9.)

J8. Esta doença limita de alguma forma suas atividades habituais (trabalhar, ir à escola, brincar, afazeres domésticos, etc.)?

J008

- 1. Sim
- 2. Não

(siga J9)

J9. _____ costuma procurar o mesmo lugar, mesmo médico ou mesmo serviço de saúde quando precisa de atendimento de saúde?

J009

- 1. Sim
- 2. Não

(Se J9=2, passe ao J11.)

J10. Quando está doente ou precisando de atendimento de saúde _____ costuma procurar:

J010

- 01. Farmácia
- 02. Unidade básica de saúde (posto ou centro de saúde ou unidade de saúde da família)
- 03. Centro de Especialidades, Policlínica pública ou PAM – Posto de Assistência Médica
- 04. UPA (Unidade de Pronto Atendimento)
- 05. Outro tipo de Pronto Atendimento Público (24 horas)
- 06. Pronto-socorro ou emergência de hospital público
- 07. Hospital público/ambulatório
- 08. Consultório particular ou clínica privada
- 09. Ambulatório ou consultório de empresa ou sindicato
- 10. Pronto-atendimento ou emergência de hospital privado
- 11. No domicílio, com profissional da equipe de saúde da família
- 12. No domicílio, com médico particular
- 13. Outro serviço

(siga J11)

J11. Quando ________ consultou um médico pela última vez?(Leia as opções resposta)

J011

- 1. Nos doze últimos meses
- 2. De 1 ano a menos de 2 anos
- 3. De 2 anos a menos de 3 anos
- 4. 3 anos ou mais
- 5. Nunca foi ao médico

(Se J11≠ 1, passe ao J13.)

J12. Quantas vezes _______ consultou o médico nos últimos 12 meses?

J012

vezes

(siga J13)

J13. Quando ___consultou um dentista pela última vez?(Leia as opções de resposta)

J013

- 1. Nos doze últimos meses
- 2. De 1 ano a menos de 2 anos
- 3. De 2 anos a menos de 3 anos
- 4. 3 anos ou mais
- 5. Nunca foi ao dentista

(siga J14)

J14. Nas duas últimas semanas, _____ procurou algum lugar, serviço ou profissional de saúde para atendimento relacionado à própria saúde?

J014

- 1. Sim
- 2. Não

(Se J14=2, passe ao J36.)

J15. Qual foi o motivo principal pelo qual _____ procurou atendimento relacionado à saúde nas duas últimas semanas?

J015

- 1. Acidente ou lesão
- 2. Doença
- 3. Problema odontológico
- 4. Reabilitação ou terapia
- 5. Continuação de tratamento
- 6. Pré-natal
- 7. Puericultura
- 8. Parto
- 9. Exame complementar de diagnóstico
- 10. Vacinação
- 11. Outro atendimento preventivo
- 12. Solicitação de atestado de saúde
- 13. Outro (

(siga J16)

J16. Onde _____ procurou o primeiro atendimento de saúde por este motivo nas duas últimas semanas?

J016

- 1. Farmácia
- 2. Unidade básica de saúde (posto ou centro de saúde ou unidade de saúde da família)
- 3. Centro de Especialidades, Policlínica pública ou PAM – Posto de Assistência Médica
- 4. UPA (Unidade de Pronto Atendimento)
- 5. Outro tipo de Pronto Atendimento Público (24 horas)
- 6. Pronto-socorro ou emergência de hospital público
- 7. Hospital público/ambulatório
- 8. Consultório particular ou clínica privada
- 9. Ambulatório ou consultório de empresa ou sindicato
- 10. Pronto-atendimento ou emergência de hospital privado
- 11. No domicílio, com profissional da equipe de saúde da família
- 12. No domicílio, com médico particular
- 13. Outro serviço (:

(siga J17)

J17. Nessa primeira vez que procurou atendimento de saúde, nas duas últimas semanas, _______ foi atendido(a)?

J017

- 1. Sim
- 2. Não

(Se J17=1, passe ao J23.
Se J17=2, siga J18.)

J18. Por que motivo _______ não foi atendido(a) na primeira vez que procurou atendimento de saúde nas duas últimas semanas?

J018

- 1. Não conseguiu vaga ou pegar senha
- 2. Não tinha médico atendendo
- 3. Não tinha dentista atendendo
- 4. Não havia serviço ou profissional de saúde especializado para atender
- 5. Esperou muito e desistiu
- 6. O serviço de saúde não estava funcionando
- 7. Os equipamentos do serviço de saúde não estavam funcionando ou disponíveis para uso
- 8. Não podia pagar pela consulta
- 9. Outro:

(siga J19)

J19. Nas duas últimas semanas, quantas vezes _______ voltou a procurar atendimento de saúde por este motivo?

J019

|__|__| vezes

- 0. Nenhuma

(Se J19≠0, siga J20.
Se J19=0, passe ao J37.)

J20. Onde ___ procurou o último atendimento de saúde por este motivo nas duas últimas semanas?

J020

- 01. Farmácia
- 02. Unidade básica de saúde (posto ou centro de saúde ou unidade de saúde da família)
- 03. Centro de Especialidades, Policlínica pública ou PAM – Posto de Assistência Médica
- 04. UPA (Unidade de Pronto Atendimento)
- 05. Outro tipo de Pronto Atendimento Público (24 horas)
- 06. Pronto-socorro ou emergência de hospital público
- 07. Hospital público/ambulatório
- 08. Consultório particular ou clínica privada
- 09. Ambulatório ou consultório de empresa ou sindicato
- 10. Pronto-atendimento ou emergência de hospital privado
- 11. No domicílio, com profissional da equipe de saúde da família
- 12. No domicílio, com médico particular
- 13. Outro serviço

(siga J21)

J21. Nessa última vez que procurou atendimento de saúde, nas duas últimas semanas, _______ foi atendido(a)?

J021

- 1. Sim
- 2. Não

(Se J21=1, passe ao J23.
Se J21=2, siga J22.)

J22. Por que motivo _______ não foi atendido(a) nessa última vez que procurou atendimento de saúde nas duas últimas semanas?

J022

- 1. Não conseguiu vaga ou pegar senha
- 2. Não tinha médico atendendo
- 3. Não tinha dentista atendendo
- 4. Não havia profissional de saúde especializado para atender
- 5. Esperou muito e desistiu
- 6. O serviço de saúde não estava funcionando
- 7. Os equipamentos do serviço de saúde não estavam funcionando ou disponíveis para uso
- 8. Não podia pagar pela consulta
- 9. Outro

(passe ao J37)

J23. Este serviço de saúde onde _______ foi atendido era: (Leia as opções de resposta)

J023

- 1. Público
- 2. Privado
- 3. Não sabe

(siga J24)

J24. Este atendimento de saúde de _______ foi coberto por algum plano de saúde?

J024

- 1. Sim
- 2. Não

(siga J25)

J25. ______ pagou algum valor por este atendimento de saúde recebido nas duas últimas semanas? (***Entrevistador**: se o(a) entrevistado(a) responder que pagou, mas teve reembolso total, marque a opção 2)*

J025

- 1. Sim
- 2. Não

(siga J26)

J26. O atendimento de _______ foi feito pelo SUS?

J026

- 1. Sim
- 2. Não
- 3. Não sabe

(siga J27)

J27. Qual foi o principal atendimento de saúde que _____ recebeu?

- 01. Consulta médica
- 02. Consulta odontológica
- 03. Consulta com outro profissional de saúde (fisioterapeuta, fonoaudiólogo, psicólogo, nutricionista, enfermeiro, etc.)
- 04. Atendimento com agente comunitário de saúde
- 05. Atendimento com parteira
- 06. Atendimento na farmácia
- 07. Vacinação
- 08. Injeção, curativo ou medição de pressão arterial
- 09. Quimioterapia, radioterapia, hemodiálise ou hemoterapia
- 10. Exames laboratoriais ou de imagem ou exames complementares de diagnóstico
- 11. Gesso ou imobilização
- 12. Pequena cirurgia em ambulatório
- 13. Internação hospitalar
- 14. Marcação de consulta
- 15. Práticas complementares como acupuntura, homeopatia e fitoterapia
- 16. Outro atendimento

J027

(Se J27≠14, siga J29.
Se J27= 14, passe ao J37.)

J29. No último atendimento de _____, foi receitado algum medicamento?

- 1. Sim
- 2. Não

J029

(Se J29=1, siga J30.
Se J29=2, passe ao J37.)

J30. _____ conseguiu obter os medicamentos receitados?
(Leia as opções de resposta)

- 1. Todos
- 2. Alguns
- 3. Nenhum

J030

(Se J30=1, passe ao J32.
Se J30= 2 ou 3, siga J31.)

J31. Qual o principal motivo de _____ não ter conseguido obter todos os medicamentos receitados?

J031

- 1. Não conseguiu obter no serviço público de saúde, pois a farmácia estava fechada
- 2. Os medicamentos não estavam disponíveis no serviço de saúde
- 3. Não conseguiu o(s) medicamento(s) no programa farmácia popular (PFP)
- 4. Não tinha farmácia próxima ou teve dificuldade de transporte
- 5. Não conseguiu encontrar todos os medicamentos na farmácia
- 6. Não tinha dinheiro para comprar
- 7. Não achou necessário
- 8. Desistiu de procurar, pois melhorou
- 9. Outro

(Se J30=3, passe ao J37. Se J30=2, siga J32.)

J32. Algum dos medicamentos foi coberto por plano de saúde?
(Leia as opções de resposta)

- 1. Sim, todos
- 2. Sim, alguns
- 3. Não, nenhum

J032

(Se J32=1, passe ao J35.
Se J32=2 ou 3, siga J33.)

J33. Algum dos medicamentos foi obtido no programa farmácia popular (PFP)? (Leia as opções de resposta)

- 1. Sim, todos
- 2. Sim, alguns
- 3. Não, nenhum

J033

(Se J33=1, passe ao J35.
Se J33=2 ou 3, siga J34.)

J34. Algum dos medicamentos foi obtido em serviço público de saúde?
Leia as opões de resposta)

- 1. Sim, todos
- 2. Sim, alguns
- 3. Não, nenhum

J034

(siga J35)

J35. _______ pagou algum valor pelos medicamentos?

- 1. Sim
- 2. Não

J035

(passe ao J37)

J36. Nas duas últimas semanas, por que motivo _____ não procurou serviço de saúde?

- 01. Não houve necessidade
- 02. Não tinha dinheiro
- 03. O local de atendimento era distante ou de difícil acesso
- 04. Horário incompatível
- 05. O atendimento é muito demorado
- 06. O estabelecimento não possuía especialista compatível com suas necessidades
- 07. Achou que não tinha direito
- 08. Não tinha quem o(a) acompanhasse
- 09. Não gostava dos profissionais do estabelecimento
- 10. Greve nos serviços de saúde
- 11. Dificuldade de transporte
- 12. Outro motivo

J036

(siga J37)

J37. Nos últimos 12 meses, _______ ficou internado(a) em hospital por 24 horas ou mais?

- 1. Sim
- 2. Não

J037

(Se J37=2, passe ao J46.)

J38. Nos últimos 12 meses, quantas vezes _____ esteve internado(a)?

J038

vezes

(siga J39)

J39. Qual foi o principal atendimento de saúde que _____ recebeu quando esteve internado(a) (pela última vez) nos doze últimos meses?

J039

- 1. Parto normal
- 2. Parto cesáreo
- 3. Tratamento clínico
- 4. Tratamento psiquiátrico
- 5. Cirurgia
- 6. Exames complementares de diagnóstico
- 7. Outro

(siga J40)

J40. Quanto tempo _____ ficou internado(a) na última vez?

Morador ficou internado

J04001 J04002

meses dias

- 88. Morador ainda está internado

(siga J41)

J41. O estabelecimento de saúde em que _____ esteve internado(a) (pela última vez) nos últimos 12 meses era: (Leia as opções de resposta)

- 1. Público
- 2. Privado
- 3. Não sabe

J041

(siga J42)

J42. A última internação de _____ nos últimos 12 meses foi coberta por algum plano de saúde?

1. Sim
2. Não

J042

(siga J43)

J43. _____ pagou algum valor por esta última internação?

***(Entrevistador**: se o(a) entrevistado(a) responder que pagou, mas teve reembolso total, marque a opção 2)*

1. Sim
2. Não

J043

(siga J44)

J44. Esta última internação de _____ foi feita através do Sistema Único de Saúde (SUS)?

1. Sim
2. Não
3. Não sabe

J044

(siga J45)

J45. Na última vez que _____ foi internado(a), como foi o atendimento recebido? (Leia as opções de resposta)

1. Muito bom
2. Bom
3. Regular
4. Ruim
5. Muito ruim

J045

(siga J46)

J46. Nos últimos 12 meses, _____ teve atendimento de emergência no domicílio?

1. Sim
2. Não

J046

(Se J46=2, passe ao J53.)

J47. Este atendimento foi coberto por algum plano de saúde?

1. Sim
2. Não

J047

(siga J48)

J48. _____ pagou algum valor por este atendimento?

***(Entrevistador**: se o(a) entrevistado(a) responder que pagou, mas teve reembolso total, marque a opção 2)*

1. Sim
2. Não

J048

(siga J49)

J49. Este atendimento foi feit através do Sistema Único de Saúde (SUS)?

1. Sim
2. Não
3. Não sabe

J049

(siga J50)

J50. Na última vez que _____ teve atendimento de urgência no domicílio, como foi o atendimento recebido? (Leia as opções de resposta)

1. Muito bom
2. Bom
3. Regular
4. Ruim
5. Muito ruim

J050

(siga J51)

J51. Neste atendimento, _____ foi transportado por ambulância para um serviço de saúde?

1. Sim
2. Não

J051

(Se J51=2, passe ao J53.)

J52. O transporte foi feito por: (Leia as opções de resposta)

1. SAMU
2. Ambulância de serviço público de saúde
3. Ambulância de serviço de saúde privado/plano de saúde
4. Corpo de Bombeiros
5. Outro

J052

(siga J53)

J53. Nos últimos 12 meses, _____ utilizou alguma prática integrativa e complementar, isto é, tratamento como acupuntura, homeopatia, plantas medicinais e fitoterapia etc.?

1. Sim
2. Não

J053

(Se J53=2, passe ao J58.)

J54. Qual tratamento _________ fez uso?(Leia as opções de resposta)

1. Acupuntura
2. Homeopatia
3. Plantas medicinais e fitoterapia
4. Outro

J054

(siga J55)

J55. Este tratamento foi coberto por algum plano de saúde?

1. Sim
2. Não

J055

(siga J56)

J56. _____ pagou algum valor por este tratamento?

***(Entrevistador**: se o(a) entrevistado(a) responder que pagou, mas teve reembolso total, marque a opção 2)*

1. Sim
2. Não

J056

(siga J57)

J57. Este tratamento foi feito através do Sistema Único de Saúde (SUS)?

1. Sim
2. Não
3. Não sabe

J057

(siga J58)

J58. _______ já teve dengue?

1. Sim
2. Não

J058

(Se J58=2, passe ao J60.)

J59. O diagnóstico foi dado por médico?

1. Sim
2. Não

J059

(siga J60)

J60. O informante desta parte foi:

1. A própria pessoa
2. Outro morador
3. Não morador

J060

(Encerre o módulo. Passe ao módulo K.)

Módulo K - Saúde dos indivíduos com 60 anos ou mais e cobertura de mamografia entre mulheres de 50 anos ou mais

Primeiramente, vamos falar sobre as dificuldades em realizar as atividades habituais.

K1. Em geral, que grau de dificuldade _____ tem para comer sozinho(a) com um prato colocado à sua frente, incluindo segurar um garfo, cortar alimentos e beber em um copo?(Leia as opções de resposta)

1. Não consegue
2. Tem grande dificuldade
3. Tem pequena dificuldade
4. Não tem dificuldade

K001

(Se K1=1, 2 ou 3, siga K2. Se K1=4, passe ao K4.)

K2. _______ recebe alguma ajuda para comer?

1. Sim
2. Não, porque não precisa
3. Não, porque não tem ajuda

K002

(Se K2=1, siga K3. Se K2=2 ou 3, passe ao K4.)

K3. Quem presta ajuda a __________ para comer? K003

1. Familiar que reside no domicílio e é remunerado para ajudar
2. Familiar que reside no domicílio e não é remunerado para ajudar
3. Familiar que não reside no domicílio e é remunerado para ajudar
4. Familiar que não reside no domicílio e não é remunerado para ajudar
5. Outra pessoa não familiar que não é remunerada para ajudar
6. Cuidador contratado
7. Empregada doméstica

(siga K4)

K4. Em geral, que grau de dificuldade ____ tem para tomar banho sozinho(a) incluindo entrar e sair do chuveiro ou banheira?(Leia as opções de resposta) K004

1. Não consegue
2. Tem grande dificuldade
3. Tem pequena dificuldade
4. Não tem dificuldade

(Se K4=1, 2 ou 3, siga K5.
Se K4=4, passe ao K7.)

K5. _____ recebe alguma ajuda para tomar banho? K005

1. Sim
2. Não, porque não precisa
3. Não, porque não tem ajuda

(Se K5=1, siga K6.
Se K5=2 ou 3, passe ao K7.)

K6. Quem presta ajuda a _____ para tomar banho? K006

1. Familiar que reside no domicílio e é remunerado para ajudar
2. Familiar que reside no domicílio e não é remunerado para ajudar
3. Familiar que não reside no domicílio e é remunerado para ajudar
4. Familiar que não reside no domicílio e não é remunerado para ajudar
5. Outra pessoa não familiar que não é remunerada para ajudar
6. Cuidador contratado
7. Empregada doméstica

(siga K7)

K7. Em geral, que grau de dificuldade ______ tem para ir ao banheiro sozinho(a) incluindo sentar e levantar do vaso sanitário?(Leia as opções de resposta) K007

1. Não consegue
2. Tem grande dificuldade
3. Tem pequena dificuldade
4. Não tem dificuldade

(Se K7=1, 2 ou 3, siga K8.
Se K7=4, passe ao K10.)

K8. ______ recebe alguma ajuda para ir ao banheiro? K008

1. Sim
2. Não, porque não precisa
3. Não, porque não tem ajuda

(Se K8=1, siga K9.
Se K8=2 ou 3, passe ao K10.)

K9. Quem presta ajuda a ______ para ir ao banheiro? K009

1. Familiar que reside no domicílio e é remunerado para ajudar
2. Familiar que reside no domicílio e não é remunerado para ajudar
3. Familiar que não reside no domicílio e é remunerado para ajudar
4. Familiar que não reside no domicílio e não é remunerado para ajudar
5. Outra pessoa não familiar que não é remunerada para ajudar
6. Cuidador contratado
7. Empregada doméstica

(siga K10)

K10. Em geral, que grau de dificuldade ______ tem para se vestir sozinho(a), incluindo calçar meias e sapatos, fechar o zíper, e fechar e abrir botões?(Leia as opções de resposta) K010

1. Não consegue
2. Tem grande dificuldade
3. Tem pequena dificuldade
4. Não tem dificuldade

(Se K10=1, 2 ou 3, siga K11.
Se K10=4, passe ao K13.)

K11. _______ recebe alguma ajuda para se vestir? K011

1. Sim
2. Não, porque não precisa
3. Não, porque não tem ajuda

(Se K11=1, siga K12.
Se K11=2 ou 3, passe ao K13.)

K12. Quem presta ajuda a _______ para se vestir? K012

1. Familiar que reside no domicílio e é remunerado para ajudar
2. Familiar que reside no domicílio e não é remunerado para ajudar
3. Familiar que não reside no domicílio e é remunerado para ajudar
4. Familiar que não reside no domicílio e não é remunerado para ajudar
5. Outra pessoa não familiar que não é remunerada para ajudar
6. Cuidador contratado
7. Empregada doméstica

(siga K13)

K13. Em geral, que grau de dificuldade _______ tem para andar em casa sozinho(a) de um cômodo a outro da casa, em um mesmo andar, como do quarto para a sala e cozinha? (Leia as opções de resposta) K013

1. Não consegue
2. Tem grande dificuldade
3. Tem pequena dificuldade
4. Não tem dificuldade

(Se K13=1, 2 ou 3, siga K14.
Se K13=4, passe ao K16.)

K14. _______ recebe alguma ajuda para andar em casa? K014

1. Sim
2. Não, porque não precisa
3. Não, porque não tem ajuda

(Se K14=1, siga K15.
Se K14=2 ou 3, passe ao K16.)

K15. Quem presta ajuda a _______para andar em casa? K015

1. Familiar que reside no domicílio e é remunerado para ajudar
2. Familiar que reside no domicílio e não é remunerado para ajudar
3. Familiar que não reside no domicílio e é remunerado para ajudar
4. Familiar que não reside no domicílio e não é remunerado para ajudar
5. Outra pessoa não familiar que não é remunerada para ajudar
6. Cuidador contratado
7. Empregada doméstica

(siga K16)

K16. Em geral, que grau de dificuldade _______ tem para deitar ou levantar da cama sozinho(a)? (Leia as opções de resposta) K016

1. Não consegue
2. Tem grande dificuldade
3. Tem pequena dificuldade
4. Não tem dificuldade

(Se K16=1, 2 ou 3, siga K17.
Se K16=4, passe ao K19.)

K17. _______ recebe alguma ajuda para deitar ou levantar da cama? K017

1. Sim
2. Não, porque não precisa
3. Não, porque não tem ajuda

(Se K17=1, siga K18.
Se K17=2 ou 3, passe ao K19.)

K18. Quem presta ajuda a __________ para deitar ou levantar da cama? K018

1. Familiar que reside no domicílio e é remunerado para ajudar
2. Familiar que reside no domicílio e não é remunerado para ajudar
3. Familiar que não reside no domicílio e é remunerado para ajudar
4. Familiar que não reside no domicílio e não é remunerado para ajudar
5. Outra pessoa não familiar que não é remunerada para ajudar
6. Cuidador contratado
7. Empregada doméstica

(siga K19)

K19. Em geral, que grau de dificuldade _______ tem para sentar ou levantar da cadeira sozinho?(Leia as opções de resposta) K019

1. Não consegue
2. Tem grande dificuldade
3. Tem pequena dificuldade
4. Não tem dificuldade

(Se K19=1, 2 ou 3, siga K20.
Se K19=4, passe ao K22.)

K20. _______ recebe alguma ajuda para sentar ou levantar da cadeira? K020

1. Sim
2. Não, porque não precisa
3. Não, porque não tem ajuda

(Se K20=1, siga K21.
Se K20=2 ou 3, passe ao K22.)

K21. Quem presta ajuda a _______ para sentar ou levantar da cadeira? K021

1. Familiar que reside no domicílio e é remunerado para ajudar
2. Familiar que reside no domicílio e não é remunerado para ajudar
3. Familiar que não reside no domicílio e é remunerado para ajudar
4. Familiar que não reside no domicílio e não é remunerado para ajudar
5. Outra pessoa não familiar que não é remunerada para ajudar
6. Cuidador contratado
7. Empregada doméstica

(siga K22)

K22. Em geral, que grau de dificuldade _______ tem para fazer compras sozinho(a), por exemplo de alimentos, roupas ou medicamentos? (Leia as opções de resposta) K022

1. Não consegue
2. Tem grande dificuldade
3. Tem pequena dificuldade
4. Não tem dificuldade

(Se K22=1, 2 ou 3, siga K23.
Se K22=4, passe ao K25.)

K23. _______ recebe alguma ajuda para fazer compras? K023

1. Sim
2. Não, porque não precisa
3. Não, porque não tem ajuda

(Se K23=1, siga K24.
Se K23=2 ou 3, passe ao K25.)

K24. Quem presta ajuda a _______ para fazer compras? K024

1. Familiar que reside no domicílio e é remunerado para ajudar
2. Familiar que reside no domicílio e não é remunerado para ajudar
3. Familiar que não reside no domicílio e é remunerado para ajudar
4. Familiar que não reside no domicílio e não é remunerado para ajudar
5. Outra pessoa não familiar que não é remunerada para ajudar
6. Cuidador contratado
7. Empregada doméstica

(siga K25)

K25. Em geral, que grau de dificuldade _______ tem para administrar as finanças sozinho(a) (cuidar do seu próprio dinheiro)? (Leia as opções de resposta). K025

1. Não consegue
2. Tem grande dificuldade
3. Tem pequena dificuldade
4. Não tem dificuldade

(Se K25=1, 2 ou 3, siga K26.
Se K25=4, passe ao K28.)

K26. _______ recebe alguma ajuda para administrar as finanças? K026

1. Sim
2. Não, porque não precisa
3. Não, porque não tem ajuda

(Se K26=1, siga K27.
Se K26=2 ou 3, passe ao K28.)

K27. Quem presta ajuda a ______ para administrar as finanças? K027

1. Familiar que reside no domicílio e é remunerado para ajudar
2. Familiar que reside no domicílio e não é remunerado para ajudar
3. Familiar que não reside no domicílio e é remunerado para ajudar
4. Familiar que não reside no domicílio e não é remunerado para ajudar
5. Outra pessoa não familiar que não é remunerada para ajudar
6. Cuidador contratado
7. Empregada doméstica

(siga K28)

K28. Em geral, que grau de dificuldade _______ tem para tomar os remédios sozinho(a)?(Leia as opções de resposta) K028

1. Não consegue
2. Tem grande dificuldade
3. Tem pequena dificuldade
4. Não tem dificuldade
5. Não faz uso de medicamento

(Se K28=1, 2 ou 3, siga K29.
Se K28= 4 ou 5, passe ao K31.)

K29. _______ recebe alguma ajuda para tomar os remédios? K029

1. Sim
2. Não, porque não precisa
3. Não, porque não tem ajuda

(Se K29=1, siga K30.
Se K29=2 ou 3, passe ao K31.)

K30. Quem presta ajuda a _________ para tomar os remédios? K030

1. Familiar que reside no domicílio e é remunerado para ajudar
2. Familiar que reside no domicílio e não é remunerado para ajudar
3. Familiar que não reside no domicílio e é remunerado para ajudar
4. Familiar que não reside no domicílio e não é remunerado para ajudar
5. Outra pessoa não familiar que não é remunerada para ajudar
6. Cuidador contratado
7. Empregada doméstica

(siga K31)

K31. Em geral, que grau de dificuldade _______ tem para ir ao médico sozinho(a)? (Leia as opções de resposta) K031

1. Não consegue
2. Tem grande dificuldade
3. Tem pequena dificuldade
4. Não tem dificuldade

(Se K31=1, 2 ou 3, siga K32.
Se K31=4, passe ao K34.)

K32. _______ recebe alguma ajuda para ir ao médico? K032

1. Sim
2. Não, porque não precisa
3. Não, porque não tem ajuda

(Se K32=1, siga K33.
Se K32=2 ou 3, passe ao K34.)

K33. Quem presta ajuda a ______ para ir ao médico? K033

- 1. Familiar que reside no domicílio e é remunerado para ajudar
- 2. Familiar que reside no domicílio e não é remunerado para ajudar
- 3. Familiar que não reside no domicílio e é remunerado para ajudar
- 4. Familiar que não reside no domicílio e não é remunerado para ajudar
- 5. Outra pessoa não familiar que não é remunerada para ajudar
- 6. Cuidador contratado
- 7. Empregada doméstica

(siga K34)

K34. Em geral, que grau de dificuldade _______ tem para sair sozinho(a) utilizando um transporte como ônibus, metrô, táxi, carro, etc.? (Leia as opções de resposta) K034

- 1. Não consegue
- 2. Tem grande dificuldade
- 3. Tem pequena dificuldade
- 4. Não tem dificuldade

(Se K34=1, 2 ou 3, siga K35.
Se K34=4, passe ao K39.)

K35. _______ recebe alguma ajuda para sair? K035

- 1. Sim
- 2. Não, porque não precisa
- 3. Não, porque não tem ajuda

(Se K35=1, siga K36.
Se K35=2 ou 3, passe ao K39.)

K36. Quem presta ajuda a _________ para sair? K036

- 1. Familiar que reside no domicílio e é remunerado para ajudar
- 2. Familiar que reside no domicílio e não é remunerado para ajudar
- 3. Familiar que não reside no domicílio e é remunerado para ajudar
- 4. Familiar que não reside no domicílio e não é remunerado para ajudar
- 5. Outra pessoa não familiar que não é remunerada para ajudar
- 6. Cuidador contratado
- 7. Empregada doméstica

(siga K39)

Rede de apoio familiar e social

Agora vamos falar sobre redes de apoio social.

K39. _______ participa de atividades sociais organizadas (clubes, grupos comunitários ou religiosos, centros de convivência do idoso etc.)? K039

- 1. Sim
- 2. Não

(siga K40)

Agora vamos falar sobre assistência de saúde

O bloco de perguntas K40 a K43 é dirigido às mulheres com 50 anos ou mais de idade. Se o morador for homem com 60 anos e mais de idade, passe ao K44.

K40. Quando foi a última vez que _____ fez um exame de mamografia? (Leia as opções de resposta) K040

- 1. Menos de 1 ano atrás
- 2. De 1 ano a menos de 2 anos
- 3. De 2 anos a menos de 3 anos
- 4. 3 anos ou mais atrás
- 5. Nunca fez

(Se K40=5, passe ao K44.)

K41. A última mamografia foi coberta por algum plano de saúde? K041

- 1. Sim
- 2. Não

(siga K42)

K42. _____ pagou algum valor pela última mamografia?
*(**Entrevistador**: se a entrevistada responder que pagou, mas teve reembolso total: marque a opção 2.)* K042

- 1. Sim
- 2. Não

(siga K43)

K43. A última mamografia foi feita através do Sistema Único de Saúde (SUS)? K043

- 1. Sim
- 2. Não
- 3. Não sabe

Se mulher (C006=2) com 60 anos ou mais, siga K44.
Se mulher (C006=2) com 50 a 59 anos, passe ao K62.

K44. Quando foi a última vez que _________ fez exame de vista por profissional de saúde?(Leia as opções de resposta) K044

- 1. Há menos de 6 meses
- 2. Entre 6 meses e menos de 1 ano
- 3. Entre 1 ano e menos de 2 anos
- 4. Entre 2 e 3 anos atrás
- 5. Mais de 3 anos atrás
- 6. Nunca fez

(Se K44=6, passe ao K52.)

K45. Algum médico já deu a _________ diagnóstico de catarata em uma ou em ambas as vistas? K045

- 1. Sim
- 2. Não

(Se K45=2, passe ao K52.)

K46. Houve indicação para realização de cirurgia nos olhos para retirar a catarata? K046

- 1. Sim
- 2. Não

(Se K46=2, passe ao K52.)

K47. _______ fez a cirurgia? K047

- 1. Sim
- 2. Não

(Se K47=1, passe ao K49.)

K48. Qual o principal motivo do(a) _______ não ter feito a cirurgia de catarata? K048

- 1. Está marcada, mas ainda não fez
- 2. Não achou necessário
- 3. Não conseguiu vaga
- 4. Estava com dificuldades financeiras
- 5. O serviço de saúde era muito distante
- 6. O plano de saúde não cobria a cirurgia
- 7. Não sabia onde realizar a cirurgia
- 8. Não tinha quem o(a) acompanhasse
- 9. Outro (

(passe ao K52)

K49. A cirurgia de _____ foi coberta pelo plano de saúde?

1. Sim
2. Não

K049

(siga K50)

K50. _______ pagou algum valor pela cirurgia?

***Entrevistador**: se o(a) entrevistado(a) responder que pagou, mas teve reembolso total: marque a opção 2*

1. Sim
2. Não

K050

(siga K51)

K51. A cirurgia foi feita através do Sistema Único de Saúde (SUS)?

1. Sim
2. Não
3. Não sabe

K051

(siga K52)

K52. Nos últimos 12 meses, _______ tomou vacina contra gripe?

1. Sim
2. Não

K052

(Se K52=1, passe ao K54.)

K53. Qual o principal motivo por não ter tomado a vacina contra gripe?

K053

1. Raramente fica gripado(a)
2. Não sabia que era necessário tomar vacina contra gripe
3. Não sabia onde tomar a vacina
4. Tem medo da reação
5. Tem medo de injeção
6. Não tinha quem o(a) acompanhasse ao serviço de saúde
7. Estava com dificuldades financeiras
8. Teve dificuldades de transporte
9. O serviço de saúde era muito distante
10. A vacina não estava disponível no serviço que procurou
11. Contra-indicação médica
12. Não acredita que a vacina protege contra gripe
13. Outro (

(siga K54)

K54. Nos últimos 12 meses, _______ teve alguma queda que o(a) levou a procurar o serviço de saúde?

1. Sim
2. Não

K054

(Se K54=2, passe ao K62.)

K55. Na ocasião dessas quedas nos últimos 12 meses, _______ fraturou quadril ou fêmur?

1. Sim
2. Não

K055

(Se K55=2, passe ao K62.)

K56. Fez cirurgia por causa dessa fratura?(Leia as opções de resposta)

1. Sim, sem colocação de prótese
2. Sim, com colocação de prótese
3. Não

K056

(Se K56=3, passe ao K62.)

K57. A cirurgia foi coberta pelo plano de saúde?

1. Sim
2. Não

K057

(siga K58)

K58. _______ pagou algum valor pela cirurgia?

***Entrevistador**: se o(a) entrevistado(a) responder que pagou, mas teve reembolso total: marque a opção 2.*

1. Sim
2. Não

K058

(siga K59)

K59. A cirurgia foi feita através do Sistema Único de Saúde (SUS)?

1. Sim
2. Não
3. Não sabe

K059

(siga K60)

K60. Quanto tempo ______ esperou desde a hora que chegou ao hospital até a realização da cirurgia?

K06001

horas (*a ser completado para menos de 24 horas*)

K06002 K06003

dias meses

(siga K61)

K61. Por quanto tempo _______ ficou internado(a) por causa dessa cirurgia?

K06101 K06102

dias meses

(siga K62)

K62. O informante desta parte foi:

1. A própria pessoa
2.Outro morador
3.Não morador

K062

(Se tiver morador(a) com menos de 2 anos passe ao módulo L. Caso contrário, encerre o módulo.)

Módulo L - Crianças com Menos de 2 Anos

As perguntas deste módulo são dirigidas às crianças do domicílio que ainda não completaram 2 anos de idade. No caso de mais de uma criança, escolher a mais nova. É importante que a mãe ou responsável pela criança seja a pessoa que responda ao questionário.

Data de referência: crianças nascidas de 28 de julho de 2011 a 27 de julho de 2013. Selecionar a mais nova.

Cuidados preventivos

As próximas perguntas são sobre cuidados preventivos, como vacinas e testes do pezinho, orelhinha, e olhinho.

L2. Com quanto tempo de vida ________ recebeu a primeira consulta médica depois da alta da maternidade?

L00201 L00202 L00203

dias meses anos

0. Nunca recebeu

(Se L2≠0, siga L3. Se L2=0, passe ao L4.)

L3. Onde foi realizada a primeira consulta médica ou de enfermagem?

1. Unidade básica de saúde (posto ou centro de saúde ou unidade de saúde da família)
2. Centro de Especialidades, Policlínica pública ou PAM – Posto de Assistência Médica
3. UPA (Unidade de Pronto Atendimento)
4. Outro tipo de Pronto Atendimento Público (24 horas)
5. Pronto-socorro ou emergência de hospital público
6. Hospital público/ambulatório
7. Consultório particular ou clínica privada
8. Ambulatório ou consultório de empresa ou sindicato
9. Pronto-atendimento ou emergência de hospital privado
10. Visita domiciliar de médico particular
11. Visita domiciliar da equipe de saúde da família
12. Outro

L003

(siga L4)

L4. Onde é realizado o acompanhamento do crescimento e desenvolvimento de ___?

1. Unidade de saúde pública (posto ou centro de saúde ou unidade de saúde da família)
2. Centro de Especialidades, Policlínica pública ou PAM – Posto de Assistência Médica
3. Hospital público/ambulatório
4. Consultório particular ou clínica privada
5. Ambulatório ou consultório de empresa ou sindicato
6. Outro (
7. Não faz acompanhamento

L004

(siga L5)

L5. Foi realizado o teste do pezinho?

1. Sim
2. Não
3. Não sabe

L005

(Se L5=1, siga L6.
Se L5=2 ou 3, passe ao L8.)

L6. Quando foi realizado o teste do pezinho? (Leia as opções de resposta)

L006

1. Na primeira semana de vida
2. Após a primeira semana e antes do primeiro mês de vida
3. Após o primeiro mês de vida
4. Não sabe

(siga L7)

L7. Quanto tempo depois da realização do exame, a sra recebeu o resultado do teste do pezinho?(Leia as opções de resposta)

1. Em 15 dias ou menos
2. Entre 16 dias e menos de 1 mês
3. Entre 1 mês e menos de 2 meses
4. Entre 2 meses e menos de 3 meses
5. Há 3 meses ou mais
6. Não recebeu

L007

(siga L8)

L8. Foi realizado o teste da orelhinha?

1. Sim
2. Não
3. Não sabe

L008

(Se L8=1, siga L9.
Se L8= 2 ou 3, passe ao L11.)

L9. Quando foi realizado o teste da orelhinha? (Leia as opções de resposta)

L009

1. Na primeira semana de vida
2. Após a primeira semana e antes do primeiro mês de vida
3. Após o primeiro mês de vida
4. Não sabe

(siga L10)

L10. Quanto tempo depois da realização do exame, a sra recebeu o resultado do teste da orelhinha?(Leia as opções de resposta)

1. Em 15 dias ou menos
2. Entre 16 dias e menos de 1 mês
3. Entre 1 mês e menos de 2 meses
4. Entre 2 meses e menos de 3 meses
5. Há 3 meses ou mais
6. Não recebeu

L010

(siga L11)

L11. Foi realizado o teste do olhinho?

1. Sim
2. Não
3. Não sabe

L011

(Se L11=1, siga L12.
Se L11=2 ou 3, passe ao L14.)

L12. Quando foi realizado o teste do olhinho? (Leia as opções de resposta)

1. Na primeira semana de vida
2. Após a primeira semana e antes do primeiro mês de vida
3. Após o primeiro mês de vida
4. Não sabe

L012

(siga L13)

L13. Quanto tempo depois da realização do exame, a sra recebeu o resultado do teste do olhinho?(Leia as opções de resposta)

1. Em 15 dias ou menos
2. Entre 16 dias e menos de 1 mês
3. Entre 1 mês e menos de 2 meses
4. Entre 2 meses e menos de 3 meses
5. Há 3 meses ou mais
6. Não recebeu

L013

(siga L14)

L14. ___ já tomou alguma vacina?

1. Sim
2. Não

L014

(Se L14=1, siga L15.
Se L14=2, passe ao L17.)

L15. A sra tem a caderneta ou cartão de ___no qual as vacinas são anotadas?

1. Sim
2. Não

L015

(Se L15=1, siga L16.
Se L15=2, passe ao L17.)

L16. Copiar as datas das vacinas Tetravalente anotadas na Caderneta da Criança:

Vacina Tetravalente (DPT + Hib)

**1ª dose**	L01613 __ __ / L01614 __ __ / L01615 __ __ __ __
**2ª dose**	L01616 __ __ / L01617 __ __ / L01618 __ __ __ __
**3ª dose**	L01619 __ __ / L01620 __ __ / L01621 __ __ __ __
**Reforço**	L01622 __ __ / L01623 __ __ / L01624 __ __ __ __

(siga L17)

L17. Você pode me dizer quais destes alimentos___ tomou ou comeu desde ontem de manhã até hoje de manhã?(Leia as opções de resposta)

a. Leite de Peito	L01701	1. Sim	2. Não	L01709	i. Feijão ou outras leguminosas (lentilha, ervilha, etc.)	1. Sim	2. Não
b. Outro leite ou derivados de leite	L01702	1. Sim	2. Não	L01710	j. Carnes ou ovos	1. Sim	2. Não
c. Água	L01703	1. Sim	2. Não	L01711	k. Batata e outros tubérculos e raízes (batata doce, mandioca)	1. Sim	2. Não
d. Chá	L01704	1. Sim	2. Não	L01712	l. Cereais e derivados (arroz, pão, cereal, macarrão, farinha, etc.)	1. Sim	2. Não
e. Mingau	L01705	1. Sim	2. Não	L01713	m. Biscoitos ou bolachas ou bolo	1. Sim	2. Não
f. Frutas ou suco natural de frutas	L01706	1. Sim	2. Não	L01714	n. Doces, balas ou outros alimentos com açúcar	1. Sim	2. Não
g. Sucos artificiais	L01707	1. Sim	2. Não	L01715	o. Refrigerantes	1. Sim	2. Não
h. Verduras/legumes	L01708	1. Sim	2. Não	L01716	p. Outros	1. Sim	2. Não

(Se b, c, d, e, ... , p todos iguais a 2, siga L18. Caso contrário, passe ao L19)

L18. Desde que ___ nasceu, tomou ou comeu outro alimento que não leite de peito?

L018

1. Sim
2. Não, somente leite de peito

(siga L19)

L19. Alguma vez ___ recebeu Sulfato Ferroso?

L019

1. Sim
2. Não
3. Recebeu um composto vitamínico, mas não sabe se contém Sulfato Ferroso

(siga L20)

L20. O informante desta parte foi:

L020

1. Mãe ou responsável
2. Outro morador
3. Não morador

(Encerre a entrevista.)

QUESTIONÁRIO DO MORADOR SELECIONADO

O adulto selecionado entre os moradores do domicílio com 18 anos ou mais de idade deve responder, individualmente, a esta parte do questionário.

Apenas no caso do indivíduo selecionado não ter condições de responder por motivo de saúde, física ou mental, solicite a outra pessoa para responder pelo indivíduo selecionado.

Módulo M. Outras características do trabalho e apoio social

Neste módulo, vamos lhe perguntar sobre as suas características de trabalho e suas relações com família e amigos.

M1. Entrevista do adulto selecionado M001

1. Realizada
2. Recusa
3. Morador não encontrado

(siga M2)

M2. Identificação da mãe do morador selecionado M002

1. Mãe moradora
2. Mãe não moradora
3. Não sabe

(Se G001=1, siga M3. Se G001=2 e E11 = 1, 2 ou 3, siga M4.
Se G001=2 e E11 não tiver sido preenchido, passe ao M14.)

M3. O informante desta parte é: M003

1. A própria pessoa
2. Outro morador
3. Não morador

Agora, vou lhe fazer algumas perguntas sobre o seu trabalho.

(Se E11 = 1, 2 ou 3, siga M5. Se E11 não tiver sido preenchido, ir para M14.)
As questões M5 a M13 devem ser respondidas apenas pelas pessoas ocupadas

M5. Em algum dos seus trabalhos, o(a) sr(a) trabalha em horário noturno? M005

1. Sim
2. Não

(Se M5=2, passe ao M9. Se M5=1, passe ao M6.)

M6. Com que frequência o(a) sr(a) trabalha em horário noturno em algum dos seus trabalhos?(Leia as opções de resposta) M006

1. Menos de 1 vez por mês
2. 1 a 3 vezes por mês
3. 1 vez por semana
4. 2 a 3 vezes por semana
5. 4 vezes por semana
6. 5 vezes ou mais por semana

(siga M7)

M7. Em algum dos seus trabalhos, o(a) sr(a) trabalha em regime de turnos ininterruptos, isto é, por 24 horas seguidas? M007

1. Sim
2. Não

(Se M7=2, passe ao M9.
Se M7=1, siga ao M8.)

M8. Com que frequência o(a) sr(a) trabalha por 24 horas seguidas?(Leia as opções de resposta) M008

1. Menos de 1 vez por mês
2. 1 a 3 vezes por mês
3. 1 vez por semana
4. 2 a 3 vezes por semana
5. 4 vezes por semana
6. 5 vezes ou mais por semana

(siga M9)

M9. O(a) sr(a) normalmente trabalha em ambientes: (Leia as opções de resposta) M009

1. Fechados
2. Abertos
3. Ambos

(Se M9=1 ou 3, siga M10. Se M9=2, passe ao M11.)

M10. Pensando em todos os seus trabalhos, durante os últimos 30 dias, alguém fumou em algum ambiente fechado onde o(a) sr(a) trabalha? M010

1. Sim
2. Não

(siga M11)

M11. Pensando em todos os seus trabalhos, o(a) sr(a) está exposto(a) a algum destes fatores que podem afetar a sua saúde?(Leia as opções de resposta)

Fator	Código			
a. Manuseio de substâncias químicas	M01101	1. Sim	2. Não	(siga M11b)
b. Exposição a ruído (barulho intenso)	M01102	1. Sim	2. Não	(siga M11c)
c. Exposição longa ao sol	M01103	1. Sim	2. Não	(siga M11d)
d. Manuseio de material radioativo (transporte, recebimento, armazenagem, trabalho com raio-x)	M01104	1. Sim	2. Não	(siga M11e)
e. Manuseio de resíduos urbanos (lixo)	M01105	1. Sim	2. Não	(siga M11f)
f. Envolvimento em atividades que levam ao nervosismo	M01106	1. Sim	2. Não	(siga M11g)
g. Exposição a material biológico (sangue, agulhas, secreções)	M01107	1. Sim	2. Não	(siga M11h)
h. Exposição a poeira industrial (pó de mármore)	M01108	1. Sim	2. Não	(siga M11i)

Se no módulo E (trabalho e rendimento) – quesito E11 = 1, siga M13.
Se no módulo E (trabalho e rendimento) – quesito E11 = 2 ou 3, leia o texto a seguir.

A próxima pergunta é referente ao trabalho principal, ou seja, aquele que o(a) sr(a) normalmente trabalha o maior número de horas. Em caso de igualdade do número de horas, o trabalho principal é o que o(a) sr(a) recebe o maior rendimento mensal. Em caso de igualdade também no rendimento mensal, o trabalho principal é o que o(a) sr(a) está há mais tempo.

M13. Há quanto tempo o(a) sr(a) está no trabalho principal?

M01301 M01302 M01303

Anos Meses Dias

(siga M14)

As próximas perguntas são sobre aspectos da sua vida com a família, amigos e algumas atividades em grupo.

M14. Com quantos familiares ou parentes o(a) sr(a) se sente à vontade e pode falar sobre quase tudo?

M014

Parentes

0. Nenhum

(siga M15)

M15. Com quantos amigos o(a) sr(a) se sente à vontade e pode falar sobre quase tudo? (sem considerar os familiares ou parentes)

M015

Amigos

0. Nenhum

(siga M16)

M16. Nos últimos 12 meses, com que frequência o(a) sr(a) participou de atividades esportivas ou artísticas em grupo?(Leia as opções de resposta)

M016

1. Mais de uma vez por semana
2. Uma vez por semana
3. De 2 a 3 vezes por mês
4. Algumas vezes no ano
5. Uma vez no ano
6. Nenhuma vez

(siga M17)

M17. Nos últimos 12 meses, com que frequência o(a) sr(a) participou de reuniões de associações de moradores ou funcionários, movimentos comunitários, centros acadêmicos ou similares?(Leia as opções de resposta)

M017

1. Mais de uma vez por semana
2. Uma vez por semana
3. De 2 a 3 vezes por mês
4. Algumas vezes no ano
5. Uma vez no ano
6. Nenhuma vez

(siga M18)

M18. Nos últimos 12 meses, com que frequência o(a) sr(a) participou de trabalho voluntário não remunerado?(Leia as opções de resposta)

M018

1. Mais de uma vez por semana
2. Uma vez por semana
3. De 2 a 3 vezes por mês
4. Algumas vezes no ano
5. Uma vez no ano
6. Nenhuma vez

(siga M19)

M19. Nos últimos 12 meses, com que frequência o(a) sr(a) compareceu a cultos ou atividades da sua religião ou de outra religião? *(sem contar com situações como casamento, batizado, ou enterro)(Leia as opções de respostas)*

M019

1. Mais de uma vez por semana
2. Uma vez por semana
3. De 2 a 3 vezes por mês
4. Algumas vezes no ano
5. Uma vez no ano
6. Nenhuma vez

(Encerre o módulo. Passe ao Módulo N.)

Módulo N. Percepção do estado de saúde

As perguntas deste módulo são sobre sua saúde em geral, tanto sobre sua saúde física como sua saúde mental.

N1. Em geral, como o(a) sr(a) avalia a sua saúde?(Leia as opções de resposta)

N001

1. Muito boa
2. Boa
3. Regular
4. Ruim
5. Muito ruim

(siga N2)

Agora vamos falar sobre as dificuldades que o(a) sr(a) tem para se locomover:

N2. O(A) sr(a) usa algum recurso como bengala, muleta, cadeira de rodas, andador ou outro equipamento para auxiliar a locomoção?

N002

1. Sim
2. Não

(siga N3)

Ao responder à próxima pergunta leve em conta o recurso que o(a) sr(a) usa para auxiliar a locomoção (se utilizar).

N3. Em geral, que grau de dificuldade o(a) sr(a) tem para se locomover?(Leia as opções de resposta)

N003

1. Nenhum
2. Leve
3. Médio
4. Intenso
5. Não consegue

(Se N3≠5, siga N4. Se N3=5, passe ao N10.)

Agora vamos perguntar sobre dor ou desconforto no peito:

N4. Quando o(a) sr(a) sobe uma ladeira, um lance de escadas ou caminha rápido no plano, sente dor ou desconforto no peito?

N004

1. Sim
2. Não
3. Não se aplica

(Se N4= 1 ou 2, siga N5. Se N4=3, N10.)

N5. Quando o(a) sr(a) caminha em lugar plano, em velocidade normal, sente dor ou desconforto no peito?

N005

1. Sim
2. Não

(Se N4 = 2 e N5 = 2, passe ao N10. Caso contrário, siga N6.)

N6. O que o(a) sr(a) faz se sente dor ou desconforto no peito?(Leia as opções de resposta)

N006

1. Para ou diminui a velocidade
2. Continua após tomar um remédio que dissolve na boca para aliviar a dor
3. Continua caminhando

(siga N7)

N7. Se o(a) sr(a) parar, o que acontece com a dor ou desconforto no peito? (Leia as opções de resposta)

N007

1. É aliviada em 10 minutos ou menos
2. É aliviada em mais de 10 minutos
3. Não é aliviada

(siga N8)

N8. O(A) sr(a) pode me mostrar onde o(a) sr(a) geralmente sente essa dor/desconforto no peito?(Leia as opções de resposta)

N008

1. Acima ou no meio do peito
2. Abaixo do peito
3. Braço esquerdo
4. Outro

(siga N10)

Agora vamos falar sobre problemas que podem ter incomodado o(a) sr(a) nas duas últimas semanas.

N10. Nas duas últimas semanas, com que frequência o(a) sr(a) teve problemas no sono, como dificuldade para adormecer, acordar frequentemente à noite ou dormir mais do que de costume? (Leia as opções de resposta)

N010

1. Nenhum dia
2. Menos da metade dos dias
3. Mais da metade dos dias
4. Quase todos os dias

(siga N11)

N11. Nas duas últimas semanas, com que frequência o(a) sr(a) teve problemas por não se sentir descansado(a) e disposto(a) durante o dia, sentindo-se cansado(a), sem ter energia? (Leia as opções de respostas)

N011

1. Nenhum dia
2. Menos da metade dos dias
3. Mais da metade dos dias
4. Quase todos os dias

(siga N12)

N12. Nas duas últimas semanas, com que frequência o(a) sr(a) teve pouco interesse ou não sentiu prazer em fazer as coisas?(Leia as opções de resposta)

N012

1. Nenhum dia
2. Menos da metade dos dias
3. Mais da metade dos dias
4. Quase todos os dias

(siga N13)

N13. Nas duas últimas semanas, com que frequência o(a) sr(a) teve problemas para se concentrar nas suas atividades habituais?(Leia as opções de resposta)

N013

1. Nenhum dia
2. Menos da metade dos dias
3. Mais da metade dos dias
4. Quase todos os dias

(siga N14)

N14. Nas duas últimas semanas, com que frequência o(a) sr(a) teve problemas na alimentação, como ter falta de apetite ou comer muito mais do que de costume?(Leia as opções de resposta)

N014

1. Nenhum dia
2. Menos da metade dos dias
3. Mais da metade dos dias
4. Quase todos os dias

(siga N15)

N15. Nas duas últimas semanas, com que frequência o(a) sr(a) teve lentidão para se movimentar ou falar, ou ao contrário, ficou muito agitado(a) ou inquieto(a)? (Leia as opções de resposta)

N015

1. Nenhum dia
2. Menos da metade dos dias
3. Mais da metade dos dias
4. Quase todos os dias

(siga N16)

N16. Nas duas últimas semanas, com que frequência o(a) sr(a) se sentiu deprimido(a), "pra baixo" ou sem perspectiva?(Leia as opções de resposta)

N016

1. Nenhum dia
2. Menos da metade dos dias
3. Mais da metade dos dias
4. Quase todos os dias

(siga N17)

N17. Nas duas últimas semanas, com que frequência o(a) sr(a) se sentiu mal consigo mesmo, se achando um fracasso ou achando que decepcionou sua família?(Leia as opções de resposta)

N017

1. Nenhum dia
2. Menos da metade dos dias
3. Mais da metade dos dias
4. Quase todos os dias

(siga N18)

N18. Nas duas últimas semanas, com que frequência o(a) sr(a) pensou em se ferir de alguma maneira ou achou que seria melhor estar morto? (Leia as opções de resposta)

N018

1. Nenhum dia
2. Menos da metade dos dias
3. Mais da metade dos dias
4. Quase todos os dias

(siga N19)

Agora vamos abordar problemas de audição e visão.

N19. O(a) sr(a) faz uso de aparelho auditivo?

N019

1. Sim
2. Não

(siga N20)

Ao responder à próxima pergunta leve em conta o **aparelho auditivo**, se o sr(a) utilizar.

N20. Em geral, que grau de dificuldade o(a) sr(a) tem para ouvir? (Leia as opções de resposta)

N020

1. Nenhum
2. Leve
3. Médio
4. Intenso
5. Não consegue

(siga N21)

N21. O(a) Sr(a) usa algum tipo de recurso (como óculos, lentes de contato etc.) para auxiliar a enxergar?

N021

1. Sim
2. Não

(siga N22)

Ao responder às duas próximas perguntas leve em conta óculos ou lente de contato ou outro recurso que o(a) sr(a) usa para auxiliar a enxergar, se utilizar.

N22. Em geral, que grau de dificuldade o(a) tem para ver de longe? (reconhecer uma pessoa conhecida do outro lado da rua a uma distância de mais ou menos 20 metros)(Leia as opções de resposta)

N022

1. Nenhum
2. Leve
3. Médio
4. Intenso
5. Não consegue

(siga N23)

N23. Em geral, que grau de dificuldade _____ tem para ver de perto? (reconhecer um objeto que esteja ao alcance das mãos ou ao ler)(Leia as opções de resposta)

N023

1. Nenhum
2. Leve
3. Médio
4. Intenso
5. Não consegue

(Encerre o módulo. Passe ao Módulo O.)

Módulo O. Acidentes e Violências

Neste módulo, abordaremos questões sobre acidentes e violências nos últimos 12 meses. Inicialmente, vamos falar sobre o uso de cinto de segurança, capacete e acidentes de trânsito.

O1. O(A) sr(a) dirige carro? O001
- 1. Sim
- 2. Não

(siga O2)

O2. O(A) sr(a) dirige motocicleta? O002
- 1. Sim
- 2. Não

(siga O3)

O3. Com que frequência o(a) sr(a) anda de carro/automóvel, van ou táxi? O003
- 1. Sempre
- 2. Quase sempre
- 3. Às vezes
- 4. Raramente
- 5. Nunca

(Se O3=5, passe ao O6. Se O3= 1 a 4, siga O4.)

O4. Com que frequência o(a) sr(a) usa cinto de segurança quando dirige ou anda como passageiro no banco da frente de carro/ automóvel, van ou táxi? (Leia as opções de resposta) O004
- 1. Nunca anda no banco da frente
- 2. Sempre usa cinto
- 3. Quase sempre usa cinto
- 4. Às vezes usa cinto
- 5. Raramente usa cinto
- 6. Nunca usa cinto

(siga O5)

O5. Com que frequência o(a) sr(a) usa cinto de segurança quando anda no banco de trás de carro/automóvel, van ou táxi? (Leia as opções de resposta) O005
- 1. Nunca anda no banco de trás
- 2. Sempre usa cinto
- 3. Quase sempre usa cinto
- 4. Às vezes usa cinto
- 5. Raramente usa cinto
- 6. Nunca usa cinto

(siga O6)

O6. Com que frequência o(a) sr(a) anda de motocicleta?(Leia as opções de resposta) O006
- 1. Sempre
- 2. Quase sempre
- 3. Às vezes
- 4. Raramente
- 5. Nunca

(Se O6 = 1 a 4 e O2 = 1, siga O7.)
(Se O6 = 1 a 4 e O2 = 2, passe ao O8.)
(Se O6 = 5, passe ao O9.)

O7. Com que frequência o(a) sr(a) usa capacete quando dirige motocicleta? (Leia as opções de resposta) O007
- 1. Sempre usa capacete
- 2. Quase sempre usa capacete
- 3. Às vezes usa capacete
- 4. Raramente usa capacete
- 5. Nunca usa capacete

(siga O8)

O8. Com que frequência o(a) sr(a) usa capacete quando anda como passageiro de motocicleta?(Leia as opções de resposta) O008
- 1. Nunca anda como passageiro de motocicleta
- 2. Sempre usa capacete
- 3. Quase sempre usa capacete
- 4. Às vezes usa capacete
- 5. Raramente usa capacete
- 6. Nunca usa capacete

(siga O9)

O9. Nos últimos 12 meses, o(a) sr(a) se envolveu em algum acidente de trânsito no qual tenha sofrido lesões corporais (ferimentos)? O009
- 1. Sim O00901 ☐☐ Quantos (siga O10)
- 2. Não

(Se O9=2, passe ao O21.)

O10. Algum desses acidentes de trânsito ocorreu quando o(a) sr(a) estava trabalhando, indo ou voltando do trabalho? O010
- 1. Sim, quando estava trabalhando
- 2. Sim, quando estava indo ou voltando do trabalho
- 3. Não

(siga O11)

O11. Durante o acidente de trânsito mais grave ocorrido nos últimos 12 meses, o(a) sr(a) era: O011
- 01. Condutor(a) de carro/van
- 02. Condutor(a) de ônibus
- 03. Condutor (a) de caminhão
- 04. Condutor(a) de motocicleta
- 05. Condutor(a) de bicicleta
- 06. Passageiro(a) de carro/van
- 07. Passageiro(a) de ônibus
- 08. Passageiro (a) de caminhão
- 09. Passageiro(a) de motocicleta
- 10. Passageiro(a) de bicicleta
- 11. Pedestre
- 12. Outro

(siga O12)

O12. Para este acidente que o(a) sr(a) considerou mais grave, o acidente envolveu transporte de carga perigosa, como gasolina, diesel, álcool, ácidos ou produtos químicos em geral? O012
- 1. Sim
- 2. Não

(Se O12 = 2, passe ao O14.
Se O12 = 1, siga O13.)

O13. O acidente resultou em derramamento de carga? O013
- 1. Sim
- 2. Não

(siga O14)

O14. Para este acidente que considerou mais grave, o(a) sr(a) deixou de realizar quaisquer de suas atividades habituais (*trabalhar, realizar afazeres domésticos, ir à escola etc.*)? O014
- 1. Sim
- 2. Não

(siga O15)

O15. Para este acidente que considerou mais grave, o(a) sr(a) recebeu algum tipo de assistência de saúde? O015
- 1. Sim
- 2. Não

(Se O15 = 2, passe ao O21.
Se O15 = 1, siga O16.)

O16. Onde o(a) sr(a) recebeu a primeira assistência de saúde? O016

- 01. No local do acidente
- 02. Unidade básica de saúde (posto ou centro de saúde ou unidade de saúde da família)
- 03. Centro de Especialidades, Policlínica pública ou PAM – Posto de Assistência Médica
- 04. UPA (Unidade de Pronto Atendimento)
- 05. Outro tipo de Pronto Atendimento Público (24 horas)
- 06. Pronto-socorro ou emergência de hospital público
- 07. Hospital público/ambulatório
- 08. Consultório particular ou clínica privada
- 09. Ambulatório ou consultório de empresa ou sindicato
- 10. Pronto-atendimento ou emergência de hospital privado
- 11. No domicílio, com médico particular
- 12. No domicílio, com médico da equipe de saúde da família
- 13. Outro

(Se O16 = 02 ao 13, passe ao O19. Se O16 = 01, siga O17.)

O17. Quem lhe prestou atendimento no local do acidente?(Leia as opções de resposta) O017

- 1. Ambulância/ Resgate do SAMU
- 2. Ambulância/ Resgate dos Bombeiros
- 3. Motos do SAMU
- 4. Ambulância/ Resgate do setor privado (particular ou convênio)
- 5. Ambulância/ Resgate da concessionária da rodovia
- 6. Outro

(siga O18)

O18. Em quanto tempo, após o acidente, o(a) sr(a) recebeu o primeiro atendimento de saúde?

O01801 O01802

Horas Minutos

(siga O19)

O19. Por causa deste acidente de trânsito, o(a) sr(a) precisou ser internado por 24 horas ou mais?

1. Sim O019 2. Não

(siga O20)

O20. O(A) sr(a) teve ou tem alguma sequela e/ou incapacidade decorrente deste acidente de trânsito?

1. Sim O020 2. Não

(siga O21)

Agora vamos perguntar sobre acidentes de trabalho.

O21. Nos últimos 12 meses o(a) sr(a) se envolveu em algum acidente de trabalho (*sem considerar os acidentes de trânsito*)?

- 1. Sim O02101 Quantos
- 2. Não O021
- 3. Não se aplica

(Se O21=2 ou 3, passe ao O25. Se O21=1, siga O22.)

O22. Para o acidente de trabalho que considerou mais grave, o(a) sr(a) deixou de realizar quaisquer de suas atividades habituais (*trabalhar, realizar afazeres domésticos, ir à escola, etc.*)?

- 1. Sim O022
- 2. Não

(siga O23)

O23. Por causa deste acidente de trabalho, o(a) sr(a) precisou ser internado por 24 horas ou mais?

- 1. Sim O023
- 2. Não

(siga O24)

O24. O(A) sr(a) teve ou tem alguma sequela e/ou incapacidade decorrente deste acidente de trabalho?

- 1. Sim O024
- 2. Não

(siga O25)

Agora vamos perguntar sobre violências e agressões.

O25. Nos últimos 12 meses, o(a) sr(a) sofreu alguma violência ou agressão de pessoa desconhecida (*como bandido, policial, assaltante etc.)?*

1. Sim O025 2. Não

(Se O25 = 2, passe ao O37. Se O25 = 1, siga ao O27.)

O27. Pensando na violência mais grave que o(a) sr(a) sofreu de pessoa desconhecida nos últimos 12 meses, que tipo de violência o(a) sr(a) sofreu? (Leia as opções de resposta) O027

- 1. Física
- 2. Sexual
- 3. Psicológica
- 4. Outra

(siga O28)

O28. Pensando na violência mais grave que o(a) sr(a) sofreu de pessoa desconhecida nos últimos 12 meses, como o(a) sr(a) foi ameaçado(a) ou ferido(a)? Leia as opções de resposta) O028

- 1. Com arma de fogo (revólver, escopeta, pistola)
- 2. Com objeto pérfuro-cortante (faca, navalha, punhal, tesoura)
- 3. Com objeto contundente (pau, cassetete, barra de ferro, pedra)
- 4. Com força corporal, espancamento (tapa, murro, empurrão)
- 5. Por meio de palavras ofensivas, xingamentos ou palavrões
- 6. Outro

(siga O29)

O29. Onde ocorreu essa violência? (Leia as opções de resposta) O029

- 1. Residência
- 2. Trabalho
- 3. Escola/Faculdade ou similar
- 4. Bar ou similar
- 5. Via pública
- 6. Banco/Caixa eletrônico/Lotérica
- 7. Outro

(siga O30)

O30. Nesta ocorrência, a violência foi cometida por: (Leia as opções de resposta)

- 1. Bandido, ladrão ou assaltante O030
- 2. Agente legal público (policial/ agente da lei)
- 3. Outro

(siga O31)

O31. Por causa dessa violência, o(a) sr(a) deixou de realizar quaisquer de suas atividades habituais (*trabalhar, realizar afazeres domésticos, ir à escola etc.*)?

1. Sim O031 2. Não

(siga O32)

O32. O(A) sr(a) teve alguma lesão corporal ou ferimento provocado por essa violência?

O032

- 1. Sim
- 2. Não

(siga O33)

O33. Por causa desta violência, o(a) sr(a) recebeu algum tipo de assistência de saúde?

O033

- 1. Sim
- 2. Não

(Se O33 = 2, passe ao O37. Se O33 = 1, siga O34.)

O34. Onde foi prestada a primeira assistência de saúde?

O034

- 01. No local da violência
- 02. Unidade básica de saúde (posto ou centro de saúde ou unidade de saúde da família)
- 03. Centro de Especialidades, Policlínica pública ou PAM – Posto de Assistência Médica
- 04. UPA (Unidade de Pronto Atendimento)
- 05. Outro tipo de Pronto Atendimento Público (24 horas)
- 06. Pronto-socorro ou emergência de hospital público
- 07. Hospital público/ambulatório
- 08. Consultório particular ou clínica privada
- 09. Ambulatório ou consultório de empresa ou sindicato
- 10. Pronto-atendimento ou emergência de hospital privado
- 11. No domicílio, com médico particular
- 12. No domicílio, com médico da equipe de saúde da família
- 13. Outro

(siga O35)

O35. Por causa desta violência, o(a) sr(a) precisou ser internado por 24 horas ou mais?

O035

- 1. Sim
- 2. Não

(siga O36)

O36. O(A) sr(a) teve ou tem alguma sequela e/ou incapacidade decorrente desta violência?

O036

- 1. Sim
- 2. Não

(siga O37)

O37. Nos últimos 12 meses, o(a) sr(a) sofreu alguma violência ou agressão de pessoa conhecida (*como pai, mãe, filho(a), cônjuge, parceiro(a), namorado(a), amigo(a), vizinho(a)*)?

O037

- 1. Sim
- 2. Não

(Se O37 = 2, passe ao Módulo P. Se O37 = 1, siga ao O38.)

O38. Nos últimos 12 meses, com que frequência sofreu alguma violência de pessoa conhecida?(Leia as opções de resposta)

O038

- 1. Uma vez
- 2. Duas vezes
- 3. De três a seis vezes
- 4. De sete a menos de 12 vezes
- 5. Pelo menos uma vez por mês
- 6. Pelo menos uma vez por semana
- 7. Quase diariamente

(siga O39)

O39. Pensando na violência mais grave que o(a) sr(a) sofreu de pessoa conhecida nos últimos 12 meses, que tipo de violência o(a) sr(a) sofreu? (Leia as opções de resposta)

O039

- 1. Física
- 2. Sexual
- 3. Psicológica
- 4. Outro

(siga O40)

O40. Pensando na violência mais grave que o(a) sr(a) sofreu de pessoa conhecida nos últimos 12 meses, como o(a) sr(a) foi ameaçado(a) ou ferido(a)?(Leia as opções de resposta)

O040

- 1. Com força corporal/espancamento (tapa, murro, beliscão, empurrão)
- 2. Com arma de fogo (revólver, escopeta, pistola)
- 3. Com objeto pérfuro-cortante (faca, navalha, punhal, tesoura)
- 4. Com objeto contundente (pau, cassetete, barra de ferro, pedra)
- 5. Com arremesso de substância/objeto quente
- 6. Com lançamento de objetos
- 7. Com envenenamento
- 8. Por meio de palavras ofensivas, xingamentos ou palavrões
- 9. Outro (

(siga O41)

O41. Onde ocorreu esta violência? (Leia as opções de resposta)

O041

- 1. Residência
- 2. Trabalho
- 3. Escola / Faculdade ou similar
- 4. Bar ou similar
- 5. Via pública
- 6. Outro

(siga O42)

O42. Nesta ocorrência, a violência foi cometida por: (Leia as opções de respostas)

O042

- 01. Cônjuge, companheiro(a), namorado(a)
- 02. Ex-cônjuge, ex-companheiro(a), ex-namorado(a)
- 03. Pai/Mãe
- 04. Padrasto/Madrasta
- 05. Filho(a)
- 06. Irmão(ã)
- 07. Outro parente
- 08. Amigos(as)/colegas
- 09. Patrão/chefe
- 10. Outra pessoa conhecida

(siga O43)

O43. Nos últimos 12 meses, o(a) sr(a) deixou de realizar quaisquer de suas atividades habituais (*trabalhar, realizar afazeres domésticos, ir à escola etc.*) por causa desta violência?

O043

- 1. Sim
- 2. Não

(siga O44)

O44. O(A) sr(a) teve alguma lesão corporal ou ferimento provocado por essa violência?

O044

- 1. Sim
- 2. Não

(siga O45)

O45. Por causa desta violência, o(a) sr(a) buscou algum tipo de assistência de saúde?

O045

- 1. Sim
- 2. Não

(Se O45 = 2, passe ao Módulo P. Se O45 = 1, siga O46.)

O46. Onde foi prestada a assistência de saúde? O046

- 01. No local da agressão
- 02. Unidade básica de saúde (posto ou centro de saúde ou unidade de saúde da família)
- 03. Centro de Especialidades, Policlínica pública ou PAM – Posto de Assistência Médica
- 04. UPA (Unidade de Pronto Atendimento)
- 05. Outro tipo de Pronto Atendimento Público (24 horas)
- 06. Pronto-socorro ou emergência de hospital público
- 07. Hospital público/ambulatório
- 08. Consultório particular ou clínica privada
- 09. Ambulatório ou consultório de empresa ou sindicato
- 10. Pronto-atendimento ou emergência de hospital privado
- 11. No domicílio, com médico particular
- 12. No domicílio, com médico da equipe de saúde da família
- 13. Outro

(siga O47)

O47. Por causa desta violência, o(a) sr(a) precisou ser internado por 24 horas ou mais? O047

- 1. Sim
- 2. Não

(siga O48)

O48. O(a) sr(a) teve ou tem alguma sequela e/ou incapacidade decorrente desta violência? O048

- 1. Sim
- 2. Não

(Encerre o módulo. Passe ao Módulo P.)

Módulo P. Estilos de Vida

Neste módulo, vou lhe fazer perguntas sobre o seu estilo de vida, como hábitos de alimentação, prática de atividade física, uso de bebidas alcoólicas e fumo.

P1. O(A) sr(a) sabe seu peso? (*mesmo que seja valor aproximado*) P001

- 1. Sim, qual? P00101 Quilograma
- 2. Não sabe

(siga P2)

P2. Quanto tempo faz que o(a) sr(a) se pesou da última vez? (Leia as opções de resposta) P002

- 1. Menos de 1 semana
- 2. Entre 1 semana e menos de 1 mês
- 3. Entre 1 mês a menos de 3 meses
- 4. Entre 3 meses e menos de 6 meses
- 5. Há 6 meses ou mais
- 6. Nunca se pesou

(Se C008 (idade) ≥ 30, siga P3. Caso contrário, passe ao P4.)

P3. O(A) sr(a) lembra qual seu peso aproximado por volta dos 20 anos de idade? (*somente para pessoas com 30 anos ou mais*) P003

- 1. Sim, qual? P00301 Quilograma
- 2. Não lembra / Não sabe

(siga P4)

P4. O(A) sr(a) sabe sua altura? (*mesmo que seja valor aproximado*) P004

- 1. Sim, qual? P00401 Centímetros
- 2. Não sabe

(Se C006 = 1, passe ao P6.) (Se C006 = 2, siga P5.)

Ser for mulher com idade entre 18 e 49 anos de idade

P5. A sra está grávida no momento? P005

- 1. Sim
- 2. Não
- 3. Não sabe

(siga P6)

Agora vou lhe fazer perguntas sobre sua alimentação.

P6. Em quantos dias da semana o(a) costuma comer feijão? P006

- Dias
- 0. Nunca ou menos de uma vez por semana

(siga P7)

P7. Em quantos dias da semana, o(a) sr(a) costuma comer salada de alface e tomate ou salada de qualquer outra verdura ou legume cru? P007

- Dias
- 0. Nunca ou menos de uma vez por semana

(Se P7=0, passe ao P9. Se P7>0, siga P8.)

P8. Em geral, quantas vezes por dia o(a) sr(a) come este tipo de salada? (Leia as opções de resposta) P008

- 1. 1 vez por dia (no almoço ou no jantar)
- 2. 2 vezes por dia (no almoço e no jantar)
- 3. 3 vezes ou mais por dia

(siga P9)

P9. Em quantos dias da semana, o(a) sr(a) costuma comer verdura ou legume cozido, como couve, cenoura, chuchu, berinjela, abobrinha? (*sem contar batata, mandioca ou inhame*) P009

- Dias
- 0. Nunca ou menos de uma vez por semana

(Se P9=0, passe ao P11. Se P9>0, siga P10.)

P10. Em geral, quantas vezes por dia o(a) sr(a) come verdura ou legume cozido? (Leia as opções de resposta) P010

- 1. 1 vez por dia (no almoço ou no jantar)
- 2. 2 vezes por dia (no almoço e no jantar)
- 3. 3 vezes ou mais por dia

(siga P11)

P11. Em quantos dias da semana o(a) sr(a) costuma comer carne vermelha (boi, porco, cabrito)? P011

- Dias
- 0. Nunca ou menos de uma vez por semana

(Se P11=0, passe ao P13. Se P11>0, siga P12.)

P12. Quando o(a) sr(a) come carne vermelha, o(a) sr(a) costuma: (Leia as opções de resposta) P012

1. Tirar o excesso de gordura visível
2. Comer com a gordura

(siga P13)

P13. Em quantos dias da semana o(a) sr(a) costuma comer frango/galinha? (Leia as opções de resposta)

P013 Dias

0. Nunca ou menos de uma vez por semana

(Se P13=0, passe ao P15. Se P13>0, siga P14.)

P14. Quando o(a) sr(a)come frango/galinha, o(a) sr(a) costuma: (Leia as opções de resposta) P014

1. Tirar a pele
2. Comer com a pele

(siga P15)

P15. Em quantos dias da semana o(a) sr(a) costuma comer peixe?

P015 Dias

0. Nunca ou menos de uma vez por semana

(siga P16)

P16. Em quantos dias da semana o(a) sr(a) costuma tomar suco de frutas natural?

P016 Dias

0. Nunca ou menos de uma vez por semana

(Se P16=0, passe ao P18. Se P16>0, siga P17.)

P17. Em geral, quantos copos por dia o(a) sr(a) toma de suco de frutas natural? (Leia as opções de resposta) P017

1. 1 copo
2. 2 copos
3. 3 copos ou mais

(siga P18)

P18. Em quantos dias da semana o(a) sr(a) costuma comer frutas?

P018 Dias

0. Nunca ou menos de uma vez por semana

(Se P18=0, passe ao P20. Se P16>0, siga P19.)

P19. Em geral, quantas vezes por dia o(a) sr(a) come frutas? (Leia as opções de resposta) P019

1. 1 vez por dia
2. 2 vezes por dia
3. 3 vezes ou mais por dia

(siga P20)

P20. Em quantos dias da semana o(a) sr(a) costuma tomar refrigerante (ou suco artificial)?

P020 Dias

0. Nunca ou menos de uma vez por semana

(Se P20=0, passe ao P23. Se P20>0, siga P21.)

P21. Que tipo de refrigerante ou suco artificial o(a) sr(a) costuma tomar? (Leia as opções de resposta) P021

1. Normal
2. Diet/Light/Zero
3. Ambos

(siga P22)

P22. Em geral, quantos copos de refrigerante ou suco artificial o(a) sr(a) costuma tomar por dia? (Leia as opções de resposta) P022

1. 1 copo
2. 2 copos
3. 3 copos ou mais

(siga P23)

P23. Em quantos dias da semana o(a) sr(a) costuma tomar leite? (*não vale leite de soja*)

P023 Dias

0. Nunca ou menos de uma vez por semana

(Se P23=0, passe ao P25. Se P23>0, siga P24.)

P24. Quando o(a) sr(a) toma leite, que tipo de leite costuma tomar? (Leia as opções de resposta) P024

1. Integral
2. Desnatado ou semidesnatado
3. Os dois tipos

(siga P25)

P25. Em quantos dias da semana o(a) sr(a) come alimentos doces, tais como pedaços de bolo ou torta, doces, chocolates, balas, biscoitos ou bolachas doces?

P025 Dias

0. Nunca ou menos de uma vez por semana

(siga P26)

P26. Em quantos dias da semana o(a) sr(a) substitui a refeição do almoço ou jantar por sanduiches, salgados ou pizzas?

P026 Dias

0. Nunca ou menos de uma vez por semana

(siga P26a)

P26a. Considerando a comida preparada na hora e os alimentos industrializados, o(a) Sr(a) acha que o seu consumo de sal é: (Leia as opções de resposta) P02601

1. Muito alto
2. Alto
3. Adequado
4. Baixo
5. Muito baixo

(siga P27)

Agora vou lhe perguntar sobre o consumo de bebidas alcoólicas.

P27. Com que frequência o(a) sr(a) costuma consumir alguma bebida alcoólica? (Leia as opções de resposta) P027

1. Não bebo nunca
2. Menos de uma vez por mês
3. Uma vez ou mais por mês

(Se P27 = 1 ou 2, passe ao P34. Se P27 = 3, siga P28.)

P28. Quantos dias por semana o(a) sr(a) costuma tomar alguma bebida alcoólica?

P028 Dias

0. Nunca ou menos de uma vez por semana

(siga P29)

P29. Em geral, no dia que o(a) sr(a) bebe, quantas doses de bebida alcoólica o(a) sr(a) consome? (*1 dose de bebida alcoólica equivale a 1 lata de cerveja, 1 taça de vinho ou 1 dose de cachaça, whisky ou qualquer outra bebida alcoólica destilada*)

P029 Doses

(Se O1 = 2 e O2 = 2, passe ao P31. Caso contrário, siga P30.)

P30. Em algum destes dias em que consumiu bebida alcoólica, o(a) sr(a) dirigiu logo depois de beber? P030

1. Sim
2. Não

(siga P31)

P31. Quantos anos o(a) sr(a) tinha quando começou a consumir bebidas alcoólicas?

P031 [|] Anos

(siga P32)

P32. Nos últimos 30 dias, o sr chegou a consumir 5 ou mais doses de bebida alcoólica em uma única ocasião? (se homem)
OU
Nos últimos 30 dias, a sra chegou a consumir 4 ou mais doses de bebida alcoólica em uma única ocasião? (se mulher)

P032

- 1. Sim
- 2. Não

(Se P32 = 2, passe ao P34. Se P32 = 1, siga ao P33.)

P33. Em quantos dias do mês isto ocorreu?(Leia as opções de resposta)

P033

- 1. 1 dia
- 2. 2 dias
- 3. 3 dias
- 4. 4 dias
- 5. 5 dias
- 6. 6 dias
- 7. 7 ou mais

(siga P34)

Agora vou lhe perguntar sobre prática de atividade física.

P34. Nos últimos três meses, o(a) sr(a) praticou algum tipo de exercício físico ou esporte? (*não considere fisioterapia*)

P034

- 1. Sim
- 2. Não

(Se P34 = 2, passe ao P38. Se P34 = 1, siga ao P35.)

P35. Quantos dias por semana o(a) sr(a) costuma praticar exercício físico ou esporte?

P035 [ ] Dias

- 0. Nunca ou menos de uma vez por semana

(Se P35=0, passe ao P38. Se P35>0, siga P36.)

P36. Qual o exercício físico ou esporte que o(a) sr(a) pratica com mais frequência?
***Entrevistador**: Anotar apenas o primeiro citado*

P036

- 01. Caminhada (não vale para o trabalho)
- 02. Caminhada em esteira
- 03. Corrida/cooper
- 04. Corrida em esteira
- 05. Musculação
- 06. Ginástica aeróbica/spinning/step/jump
- 07. Hidroginástica
- 08. Ginástica em geral/localizada/pilates/alongamento/ioga
- 09. Natação
- 10. Artes marciais e luta
- 11. Bicicleta/bicicleta ergométrica
- 12. Futebol
- 13. Basquetebol
- 14. Voleibol
- 15. Tênis
- 16. Dança (com o objetivo de praticar atividade física)
- 17. Outro

(siga P37)

P37. Em geral, no dia que o(a) sr(a) pratica exercício ou esporte, quanto tempo dura esta atividade?

P03701 [|] Horas P03702 [|] Minutos

As questões P38 a P41 são dirigidas às pessoas ocupadas.

(Se E11 = 1, 2 ou 3, siga P38. Se E11 não tiver sido preenchido, passe ao P42.)

P38. No seu trabalho, o(a) sr(a) anda bastante a pé?

P038

- 1. Sim
- 2. Não

(siga P39)

P39. No seu trabalho, o(a) sr(a) faz faxina pesada, carrega peso ou faz outra atividade pesada que requer esforço físico intenso?

P039

- 1. Sim
- 2. Não

(Se P39 = 1, siga P39a.
Se P39 = 2, passe ao P40.)

P39a. Em uma semana normal, em quantos dias o(a) sr(a) faz essas atividades no seu trabalho?

P03901 [ ] Número de dias

(siga P39b)

P39b. Quanto tempo o(a) sr(a) passa realizando atividades físicas em um dia normal de trabalho?

P03902 [|] Horas P03903 [|] Minutos

(siga P40)

P40. Para ir ou voltar do trabalho, o(a) sr(a) faz algum trajeto a pé ou de bicicleta?(Leia as opções de resposta)

P040

- 1. Sim, todo o trajeto
- 2. Sim, parte do trajeto
- 3. Não

(Se P40 = 3, passe ao P42.
Se P40 = 1 ou 2, siga P41.)

P41. Quanto tempo o(a) sr(a) gasta, por dia, para percorrer este trajeto a pé ou de bicicleta, considerando a <u>ida e a volta</u> do trabalho?

P04101 [|] Horas P04102 [|] Minutos

(siga P42)

P42. Nas suas atividades habituais (tais como ir a algum curso, escola ou clube ou levar alguém a algum curso, escola ou clube), quantos dias por semana o(a) sr(a) faz alguma atividade que envolva deslocamento a pé ou bicicleta?

P042 [ ] Dias

- 0. Nunca ou menos de uma vez por semana

(Se P42 = 0, passe ao P44.
Se P42>0, siga P43.)

P43. No dia em que o(a) sr(a) faz esta atividade, quanto tempo o(a) sr(a) gasta no deslocamento a pé ou de bicicleta, considerando a <u>ida e a volta</u>?

P04301 [|] Horas P04302 [|] Minutos

(siga P44)

P44. Nas suas atividades domésticas, o(a) sr(a) faz faxina pesada, carrega peso ou faz outra atividade pesada que requer esforço físico intenso?

P044

- 1. Sim
- 2. Não

(Se P44=1, siga P44a.
Se P44=2, passe ao P45.)

P44a. Em uma semana normal, nas suas atividades domésticas, em quantos dias o(a) sr(a) faz faxina pesada ou realiza atividades que requerem esforço físico intenso?

P04401 [ ] Número de dias

(siga P44b)

P44b. Quanto tempo gasta, por dia, realizando essas atividades domésticas pesadas?

P04403 [|] Horas P04404 [|] Minutos

(siga P45)

P45. Em média, quantas horas por dia o(a) sr(a) costuma ficar assistindo televisão?(leia as opções de resposta)

P045

1. Menos de 1 hora
2. Entre 1 horas e menos de 2 horas
3. Entre 2 horas e menos de 3 horas
4. Entre 3 horas e menos de 4 horas
5. Entre 4 horas e menos de 5 horas
6. Entre 5 horas e menos de 6 horas
7. 6 horas ou mais
8. Não assiste televisão

(siga P46)

P46. Perto do seu domicílio, existe algum lugar público (praça, parque, rua fechada, praia) para fazer caminhada, realizar exercício ou praticar esporte?

P046

1. Sim
2. Não

(siga P47)

Agora vou lhe perguntar sobre a participação em programas públicos de atividade física

P47. O(A) sr(a) conhece algum programa público no seu município de estímulo à prática de atividade física?

P047

1. Sim
2. Não

(Se P47 = 2, passe ao P50.
Se P47 = 1, siga P48.)

P48. O(A) sr(a) participa desse programa?

P048

1. Sim
2. Não

(Se P48 = 2, siga P49.
Se P48 = 1, passe ao P50.)

P49. Qual o principal motivo de não participar?(Leia as opções de resposta)

P049

1. Não é perto do meu domicílio
2. Não tenho tempo
3. Não tenho interesse nas atividades oferecidas
4. O espaço não é seguro/iluminado
5. Foi impedido de participar
6. Problemas de saúde ou incapacidade física
7. Outro

(siga P50)

Agora vou lhe perguntar sobre fumo de cigarros ou de outros produtos do tabaco que são fumados tais como charuto, cigarrilha, cachimbo, cigarros de cravo (ou de Bali) e narguilé (ou cachimbos d´água). Por favor, não responda sobre produtos de tabaco que não fazem fumaça como rapé e fumo para mascar. Não considere, também, cigarros de maconha.

P50. Atualmente, o(a) sr(a) fuma algum produto do tabaco?
(Leia as opções de resposta)

P050

1. Sim, diariamente
2. Sim, menos que diariamente
3. Não fumo atualmente

(Se P50 = 1, passe ao P53.
Se P50 = 2, siga P51.
Se P50 = 3, passe ao P52.)

P51. E no passado, o(a) sr(a) fumou algum produto do tabaco diariamente?

P051

1. Sim
2. Não

(Se P51 = 1, passe ao P53.
Se P51 = 2, passe ao P54.)

P52. E no passado, o(a) sr(a) fumou algum produto do tabaco?
(Leia as opções de resposta)

P052

1. Sim, diariamente
2. Sim, menos que diariamente
3. Não, nunca fumei

((Se P52 = 1, siga P53.
Se P52 = 2, passe ao P58.
Se P52 = 3, passe ao P67.)

P53. Que idade o(a) sr(a) tinha quando começou a fumar cigarro diariamente?

P053

Anos

(Se P52 = 1, passe ao P58.
Caso contrário, siga P54.)

P54. Em média, quantos dos seguintes produtos o(a) sr(a) fuma por dia ou por semana atualmente?(Leia as opções de resposta)

a. Cigarros industrializados?

P05401

1. Um ou mais por dia — P05402 Quantos por dia
2. Um ou mais por semana — P05403 Quantos por semana
3. Menos que uma vez por semana
4. Menos do que um por mês
5. Não fuma este produto

(siga P54b)

b. Cigarros de palha ou enrolados a mão?

P05404

1. Um ou mais por dia — P05405 Quantos por dia
2. Um ou mais por semana — P05406 Quantos por semana
3. Menos que uma vez por semana
4. Menos do que um por mês
5. Não fuma este produto

(siga P54c)

c. Cigarros de cravo ou de Bali?

P05407

1. Um ou mais por dia — P05408 Quantos por dia
2. Um ou mais por semana — P05409 Quantos por semana
3. Menos que uma vez por semana
4. Menos do que um por mês
5. Não fuma este produto

(siga P54d)

d. Cachimbos (considere cachimbos cheios)?

P05410

1. Um ou mais por dia — P05411 Quantos por dia
2. Um ou mais por semana — P05412 Quantos por semana
3. Menos que uma vez por semana
4. Menos do que um por mês
5. Não fuma este produto

(siga P54e)

e. Charutos ou cigarrilhas? P05413

1. Um ou mais por dia — P05414 Quantos por dia

2. Um ou mais por semana — P05415 Quantos por semana

3. Menos que uma vez por semana

4. Menos do que um por mês

5. Não fuma este produto

(siga P54f)

f. Narguilé (sessões)? P05416

1. Um ou mais por dia — P05417 Quantos por dia

2. Um ou mais por semana — P05418 Quantos por semana

3. Menos que uma vez por semana

4. Menos do que um por mês

5. Não fuma este produto

(siga P54g)

g. Outro? P05419

1. Um ou mais por dia — P05421 Quantos por dia

2. Um ou mais por semana — P05422 Quantos por semana

3. Menos que uma vez por semana

4. Menos do que um por mês

5. Não fuma este produto

(Se P50=1, siga P55. Se P50=2 e P54a ≠5, passe ao P56. Se P50 =2 e P54a=5, passe ao P60.)

P55. Quanto tempo depois de acordar o(a) sr(a) normalmente fuma pela primeira vez?(leia as opções de resposta) P055

1. Até 5 minutos

2. De 6 a 30 minutos

3. De 31 a 60 minutos

4. Mais de 60 minutos

(Se P54a = 5, passe ao P60. Caso contrário, siga P56.)

As próximas perguntas são referentes à última vez que o(a) sr(a) comprou cigarros industrializados para consumo próprio.

P56. A última vez em que o(a) sr(a) comprou cigarros para uso próprio, quantos cigarros comprou? P056

***Entrevistador**: Registre a quantidade e, quando necessário, registre os detalhes da unidade.*

UNIDADE	QUANTIDADE	DETALHE DA UNIDADE
1. Cigarros	P05601	
2. Maços	P05602	P05603 Quantos cigarros havia em cada maço
3. Pacotes	P05604	P05605 Quantos maços havia em cada pacote
4. Nunca comprei cigarros para uso próprio		

(Se P56=1, 2 ou 3, siga P57. Se P56=4, passe ao P60.)

P57. No total, quanto o(a) sr(a) pagou por essa compra?

R$ P057

(passe ao P60)

P58. Em média, quantos cigarros industrializados o(a) sr(a) fumava por dia ou por semana? (Leia as opções de resposta) P058

1. Um ou mais por dia — P05801 Quantos por dia

2. Um ou mais por semana — P05802 Quantos por semana

3. Menos que uma vez por semana

4. Menos do que um por mês

5. Não fumava este produto

6. Não sabe

(siga P59)

P59. Há quanto tempo o(a) sr(a) parou de fumar?

P05901	P05902	P05903	P05904
Anos	Meses	Semanas	Dias

(Se há menos de 1 ano, passe ao P61.
Se há 1 ano ou mais, passe ao P67.)

As próximas perguntas são sobre as tentativas de parar de fumar que o(a) sr(a) fez nos últimos 12 meses.

P60. Durante os últimos 12 meses, o(a) sr(a) tentou parar de fumar? P060

1. Sim

2. Não

(Se P60 = 2, passe ao P67. Se P60 = 1, siga P61.)

P61. Quando o(a) sr(a) tentou parar de fumar, procurou tratamento com profissional de saúde? P061

1. Sim

2. Não

(Se P61= 2, passe ao P67. Se P61 = 1, siga P62.)

P62. O(A) sr(a) conseguiu o tratamento com profissional de saúde? P062

1. Sim

2. Não

(Se P62 = 1, passe ao P64. Se P62 = 2, siga P63.)

P63. Por que o(a) sr(a) não conseguiu tratamento? P063

01. A consulta está marcada, mas ainda não foi realizada

02. O tempo de espera no serviço de saúde era muito grande

03. Não conseguiu marcar

04. Não sabia quem procurar ou aonde ir

05. Estava com dificuldades financeiras

06. O plano de saúde não cobria o tratamento

07. O serviço de saúde era muito distante

08. Teve dificuldades de transporte

09. O horário de funcionamento do serviço de saúde era incompatível com as atividades de trabalho ou domésticas

10. Outro

(passe ao P67)

P64. O tratamento foi coberto por algum plano de saúde? P064

1. Sim 2. Não

(siga P65)

P65. O(A) sr(a) pagou algum valor por esse tratamento? P065

***(Entrevistador**: Se o(a) entrevistado(a) responder que pagou mas teve reembolso total, marque a opção 2)*

1. Sim 2. Não

(siga P66)

P66. O tratamento foi feito através do Sistema Único de Saúde (SUS)? P066

1. Sim 2. Não 3. Não sabe

(siga P67)

A próxima pergunta é sobre o uso de tabaco sem fumaça, como fumo para mascar ou para aspirar ou algum produto do tabaco que não faz fumaça. Não considere o uso de cocaína e outras drogas.

P67. Atualmente, o(a) sr(a) masca fumo, usa rapé ou usa algum produto do tabaco que não faz fumaça?(leia as opções de resposta) P067

1. Sim, diariamente 2. Sim, menos que diariamente 3. Não usa

(siga P68)

Agora eu gostaria de lhe fazer perguntas sobre fumo em seu domicílio.

P68. Com que frequência alguém fuma dentro do seu domicílio?(Leia as opções de resposta) P068

1. Diariamente 2. Semanalmente 3. Mensalmente 4. Menos que mensalmente 5. Nunca

(siga P69)

A próxima pergunta se refere à sua exposição à propaganda a favor de cigarros.

P69. Nos últimos 30 dias, o(a) sr(a) viu alguma propaganda ou anúncio de cigarros nos pontos de venda de cigarros? P069

1. Sim 2. Não 3. Não lembra

(siga P70)

As próximas perguntas se referem à sua exposição à propaganda contra cigarros.

P70. Nos últimos 30 dias, o(a) sr(a) viu ou ouviu informações sobre os riscos de fumar cigarros ou que estimulem a parar de fumar nos seguintes meios de comunicação? (Leia as opções de resposta)

a. Nos jornais ou revistas? P07001

1. Sim 2. Não 3. Não sabe

(siga P70b)

b. Na televisão? P07002

1. Sim 2. Não 3. Não sabe

(siga P70c)

c. No rádio? P07003

1. Sim 2. Não 3. Não sabe

(siga P71)

P71. Nos últimos 30 dias, viu alguma foto ou advertência sobre os riscos de fumar nos maços de cigarros? P071

1. Sim 2. Não 3. Não vi nenhum maço de cigarros

(Se P71 = 2 ou 3, passe ao Módulo Q.)
(Se P71 = 1 e P50 = 1 ou 2, siga P72.
Se P71 = 1 e P50 = 3, passe ao Módulo Q.)

P72. Nos últimos 30 dias, as advertências nos maços de cigarro levaram o(a) sr(a) a pensar em parar de fumar? P072

1. Sim 2. Não

(Encerre o módulo. Passe ao Módulo Q.)

Módulo Q. Doenças crônicas

As perguntas deste módulo são sobre doenças crônicas. Vamos fazer perguntas sobre diagnóstico de doenças, uso dos serviços de saúde e tratamento dos problemas.

Q1. Quando foi a última vez que o(a) sr(a) teve sua pressão arterial medida?(Leia as opções de resposta) Q001

1. Há menos de 6 meses
2. Entre 6 meses e menos de 1 ano
3. Entre 1 ano e menos de 2 anos
4. Entre 2 anos e menos de 3 anos
5. 3 anos ou mais
6. Nunca

(Se Q1=1 a 5, siga Q2. Se Q1=6, passe ao Q29.)

Q2. Algum médico já lhe deu o diagnóstico de hipertensão arterial (pressão alta)? Q002

1. Sim
2. Apenas durante a gravidez (só para mulheres)
3. Não

(Se Q2=1, siga Q3. Se Q2=2 ou 3, passe ao Q29.)

Q3. Que idade o(a) sr(a) tinha no primeiro diagnóstico de hipertensão arterial (pressão alta)? Q003

Anos

0. Menos de 1 ano

(siga Q4)

Q4. O(A) sr(a) vai ao médico/serviço de saúde regularmente por causa da hipertensão arterial (pressão alta)? Q004

1. Sim
2. Não, só quando tem algum problema
3. Nunca vai

(Se Q4 = 2 ou 3, siga Q5.
Se Q4 = 1, passe ao Q6.)

Q5. Qual o principal motivo do(a) sr(a) não visitar o médico/serviço de saúde regularmente por causa da hipertensão arterial (pressão alta)? Q005

1. O serviço de saúde é muito distante
2. O tempo de espera no serviço de saúde é muito grande
3. Tem dificuldades financeiras
4. Não acha necessário
5. O horário de funcionamento do serviço de saúde é incompatível com suas atividades de trabalho ou domésticas
6. O plano de saúde não cobre as consultas
7. Não sabe quem procurar ou aonde ir
8. Dificuldade de transporte
9. Outro

(siga Q6)

Q6. Nas duas últimas semanas, o(a) sr(a) tomou medicamentos por causa da hipertensão arterial (pressão alta)? Q006

1. Sim
2. Não

(Se Q6=1, siga Q7
Se Q6=2, passe ao Q11.)

Q7. Algum dos medicamentos para hipertensão arterial foi coberto por plano de saúde? (Leia as opções de resposta) Q007

1. Sim, todos
2. Sim, alguns
3. Não, nenhum

(Se Q7 = 1, passe a Q10.
Se Q7 = 2 ou 3, siga Q8.)

Q8. Algum dos medicamentos para hipertensão arterial foi obtido no programa farmácia popular (PFP)?(Leia as opções de resposta) Q008

1. Sim, todos
2. Sim, alguns
3. Não, nenhum

(Se Q8 = 1, passe a Q10.
Se Q8 = 2 ou 3, siga Q9.)

Q9. Algum dos medicamentos para hipertensão arterial foi obtido em serviço público de saúde?(Leia as opções de resposta) Q009

1. Sim, todos
2. Sim, alguns
3. Não, nenhum

(siga Q10)

Q10. O(A) sr(a) pagou algum valor pelos medicamentos? Q010

1. Sim
2. Não

(siga Q11)

Q11. Quando foi a última vez que o(a) sr(a) recebeu assistência médica por causa da hipertensão arterial?(Leia as opções de resposta) Q011

1. Há menos de 6 meses
2. Entre 6 meses e menos de 1 ano
3. Entre 1 ano e menos de 2 anos
4. Entre 2 anos e menos de 3 anos
5. Há 3 anos ou mais
6. Nunca recebeu

(Se Q11 = 1 a 5, siga Q12. Se Q11 = 6, passe ao Q28.)

Q12. Na última vez que recebeu assistência médica para hipertensão arterial, onde o(a) sr(a) foi atendido? Q012

01. Unidade básica de saúde (posto ou centro de saúde ou unidade de saúde da família)
02. Centro de Especialidades, Policlínica pública ou PAM - Posto de Assistência Médica
03. UPA (Unidade de pronto Atendimento)
04. Outro tipo de Pronto Atendimento Público (24 horas)
05. Pronto-socorro ou emergência de hospital público
06. Hospital público/ambulatório
07. Consultório particular ou clínica privada
08. Ambulatório ou consultório de empresa ou sindicato
09. Pronto-atendimento ou emergência de hospital privado
10. No domicílio, com médico da equipe de saúde da família
11. No domicílio, com médico particular
12. Outro

(siga Q13)

Q13. Esse atendimento foi coberto por algum plano de saúde? Q013

1. Sim
2. Não

(siga Q14)

Q14. O(A) sr(a) pagou algum valor por este atendimento? Q014

***(Entrevistador**: Se o(a) entrevistado(a) responder que pagou mas teve reembolso total, marque a opção 2)*

1. Sim
2. Não

(siga Q15)

Q15. Esse atendimento foi feito pelo SUS? Q015

1. Sim
2. Não
3. Não sabe

(siga Q16)

Q16. Na última consulta, o médico que o(a) atendeu era o mesmo das consultas anteriores? Q016

1. Sim
2. Não

(siga Q17)

Q17. Na última consulta, o médico viu os exames das consultas passadas? Q017

1. Sim
2. Não
3. Não, pois não tinha realizado exames

(siga Q18)

Q18. Em algum dos atendimentos para hipertensão, algum médico ou outro profissional de saúde lhe deu alguma dessas recomendações?(Leia as opções de resposta)

a. Manter uma alimentação saudável (com frutas e vegetais) Q01801	1. Sim	2. Não	(siga Q18b)
b. Manter o peso adequado Q01802	1. Sim	2. Não	(siga Q18c)
c. Ingerir menos sal Q01803	1. Sim	2. Não	(siga Q18d)
d. Praticar atividade física regular Q01804	1. Sim	2. Não	(siga Q18e)
e. Não fumar Q01805	1. Sim	2. Não	(siga Q18f)
f. Não beber em excesso Q01806	1. Sim	2. Não	(siga Q18g)
g. Fazer o acompanhamento regular Q01807	1. Sim	2. Não	(siga Q18h)
h. Outro Q01808	1. Sim	2. Não	

(siga Q19)

Q19. Em algum dos atendimentos para hipertensão arterial foi pedido algum exame?(Leia as opções de resposta)

a. Exame de sangue (colesterol, glicemia, triglicerídeos) Q01901	1. Sim	2. Não	(siga Q19b)
b. Exame de urina Q01902	1. Sim	2. Não	(siga Q19c)
c. Eletrocardiograma Q01903	1. Sim	2. Não	(siga Q19d)
d. Teste de esforço Q01904	1. Sim	2. Não	(siga Q19e)
e. Outro Q01905	1. Sim	2. Não	

(Se todos os itens forem = 2, passe ao Q22. Caso contrário, siga Q20.)

Q20. O(A) sr(a) fez todos os exames solicitados? Q020

1. Sim
2. Não

(Se Q20 = 1, passe ao Q22.
Se Q20 = 2, siga ao Q21.)

Q21. Qual o principal motivo do(a) sr(a) não ter feito todos os exames solicitados? Q021

01. O exame está marcado, mas ainda não fez
02. Não achou necessário
03. Não conseguiu marcar
04. O tempo de espera no laboratório ou serviço de saúde era muito grande
05. Estava com dificuldades financeiras
06. O laboratório ou serviço de saúde era muito distante
07. O horário de funcionamento do laboratório ou serviço de saúde era incompatível com as suas atividades de trabalho ou domésticas
08. O plano de saúde não cobria todos os exames solicitados
09. Não sabia onde realizar os exames
10. Dificuldade de transporte
11. Outro

(siga Q22)

Q22. Em algum dos atendimentos para hipertensão arterial, houve encaminhamento para alguma consulta com médico especialista, tais como cardiologista ou nefrologista? Q022

- 1. Sim
- 2. Não
- 3. Não houve encaminhamento, pois todas as consultas para hipertensão foram com médico especialista

(Se Q22 = 1, siga Q23. Se Q22 = 2 ou 3, passe ao Q26.)

Q23. O(A) sr(a) foi a todas as consultas com o médico especialista? Q023

- 1. Sim
- 2. Não

(Se Q23 = 1, passe ao Q26. Se Q23 = 2, siga Q24.)

Q24. Qual o principal motivo do(a) sr(a) não ter ido a todas as consultas com o médico especialista? Q024

- 01. A consulta está marcada, mas a consulta ainda não foi realizada
- 02. Não achou necessário
- 03. Não sabia quem procurar ou aonde ir
- 04. Estava com dificuldades financeiras
- 05. Teve dificuldades de transporte
- 06. Não conseguiu marcar
- 07. O tempo de espera no serviço de saúde era muito grande
- 08. O plano de saúde não cobria a consulta
- 09. O serviço de saúde era muito distante
- 10. O horário de funcionamento do serviço de saúde era incompatível com as atividades de trabalho ou domésticas
- 11. Outro

(siga Q26)

Q26. Alguma vez o(a) sr(a) se internou por causa da hipertensão ou de alguma complicação? Q026

- 1. Sim
- 2. Não

(Se Q26=1, siga Q27. Se Q26=2, passe ao Q28.)

Q27. Há quanto tempo foi a última internação por causa da hipertensão ou de alguma complicação? (Leia as opções de resposta) Q027

- 1. Há menos de 6 meses
- 2. Entre 6 meses e menos de 1 ano
- 3. Entre 1 ano e menos de 2 anos
- 4. Entre 2 anos e menos de 3 anos
- 5. Há 3 anos ou mais

(siga Q28)

Q28. Em geral, em que grau a hipertensão ou alguma complicação da hipertensão limita as suas atividades habituais (*como trabalhar, estudar, realizar afazeres domésticos, etc*)?(Leia as opções de resposta) Q028

- 1. Não limita
- 2. Um pouco
- 3. Moderadamente
- 4. Intensamente
- 5. Muito intensamente

(siga Q29)

Q29. Quando foi a última vez que o(a) sr(a) fez exame de sangue para medir a glicemia, isto é, o açúcar no sangue?(Leia as opções de resposta) Q029

- 1. Há menos de 6 meses
- 2. Entre 6 meses e menos de 1 ano
- 3. Entre 1 ano e menos de 2 anos
- 4. Entre 2 anos e menos de 3 anos
- 5. Há 3 anos ou mais
- 6. Nunca fez

(Se Q29=1 a 5, siga Q30. Se Q29=6, passe ao Q59.)

Q30. Algum médico já lhe deu o diagnóstico de diabetes? Q030

- 1. Sim
- 2. Apenas durante a gravidez (*só para mulheres*)
- 3. Não

(Se Q30=1, siga Q31. Se Q30=2 ou 3, passe ao Q59.)

Q31. Que idade o(a) sr(a) tinha no primeiro diagnóstico de diabetes? Q031

__ __ Anos

0. Menos de 1 ano

(siga Q32)

Q32. O(A) sr(a) vai ao médico/serviço de saúde regularmente por causa do diabetes? Q032

- 1. Sim
- 2. Não, só quando tem algum problema
- 3. Nunca vai

(Se Q32=1, passe ao Q34. Se Q32=2 ou 3, siga Q33.)

Q33. Qual o principal motivo do(a) sr(a) não visitar o médico/serviço de saúde regularmente por causa do diabetes? Q033

- 1. O serviço de saúde é muito distante
- 2. O tempo de espera no serviço de saúde é muito grande
- 3. Tem dificuldades financeiras
- 4. Não acha necessário
- 5. O horário de funcionamento do serviço de saúde é incompatível com suas atividades de trabalho ou domésticas
- 6. O plano de saúde não cobre as consultas
- 7. Não sabe quem procurar ou aonde ir
- 8. Dificuldade de transporte
- 9. Outro

(siga Q34)

Q34. Nas duas últimas semanas, por causa do diabetes, o(a) sr(a):

a. Tomou medicamentos orais para baixar o açúcar? Q03401

- 1. Sim
- 2. Não

(siga Q34b)

b. Usou insulina? Q03402

- 1. Sim
- 2. Não

(Se Q34a=1 ou Q34b=1, siga Q35. Se Q34a=2 e Q34b=2, passe ao Q39.)

Q35. Algum dos medicamentos ou insulina para diabetes foi coberto por plano de saúde?(Leia as opções de resposta) Q035

- 1. Sim, todos
- 2. Sim, alguns
- 3. Não, nenhum

(Se Q35=1, passe ao Q38. Se Q35=2 ou 3, siga Q36.)

Q36. Algum dos medicamentos para diabetes ou insulina foi obtido no Programa de Farmácia Popular (PFP)? (Leia as opções de resposta) Q036

- 1. Sim, todos
- 2. Sim, alguns
- 3. Não, nenhum

(Se Q36=1, passe ao Q38. Se Q36=2 ou 3, siga Q37.)

Q37. Algum dos medicamentos para diabetes ou insulina foi obtido em serviço público de saúde? .(Leia as opções de resposta) Q037

- 1. Sim, todos
- 2. Sim, alguns
- 3. Não, nenhum

(siga Q38)

Q38. O(A) sr(a) pagou algum valor pelos medicamentos para diabetes ou insulina? Q038

- 1. Sim
- 3. Não

(siga Q39)

Q39. Quando foi a última vez que o(a) sr(a) recebeu assistência médica por causa do diabetes? Q039

1. Há menos de 6 meses
2. Entre 6 meses e menos de 1 ano
3. Entre 1 ano e menos de 2 anos
4. Entre 2 anos e menos de 3 anos
5. Há 3 anos ou mais
6. Nunca recebeu

(Se Q39=1 ao 5, siga Q40. Se Q39=6, passe ao Q58.)

Q40. Na última vez que recebeu assistência médica para diabetes, onde o(a) sr(a) foi atendido? Q040

01. Unidade básica de saúde (posto ou centro de saúde ou unidade de saúde da família)
02. Centro de Especialidades, Policlínica pública ou PAM - Posto de Assistência Médica
03. UPA (Unidade de pronto Atendimento)
04. Outro tipo de Pronto Atendimento Público´ (24 horas)
05. Pronto-socorro ou emergência de hospital público
06. Hospital público/ambulatório
07. Consultório particular ou clínica privada
08. Ambulatório ou consultório de empresa ou sindicato
09. Pronto-atendimento ou emergência de hospital privado
10. No domicílio, com médico da equipe de saúde da família
11. No domicílio, com médico particular
12. Outro

(siga Q41)

Q41. Esse atendimento foi coberto por algum plano de saúde? Q041

1. Sim
2. Não

(siga Q42)

Q42. O(A) sr(a) pagou algum valor por esse atendimento? ***(Entrevistador**: Se o(a) entrevistado (a) responder que pagou mas teve reembolso total, marque a opção 2)* Q042

1. Sim
2. Não

(siga Q43)

Q43. Esse atendimento foi feito pelo SUS? Q043

1. Sim
2. Não
3. Não sabe

(siga Q44)

Q44. Na última consulta, o médico que o(a) atendeu era o mesmo das consultas anteriores? Q044

1. Sim
2. Não

(siga Q45)

Q45. Na última consulta, o médico viu os exames das consultas passadas? Q045

1. Sim
2. Não
3. Não, pois não tinha realizado exames

(siga Q46)

Q46. Em algum dos atendimentos para diabetes, algum médico ou outro profissional de saúde lhe deu alguma dessas recomendações?(Leia as opções de resposta)

a. Manter uma alimentação saudável (com frutas e vegetais)	Q04601	1. Sim	2. Não	(siga Q46b)
b. Manter o peso adequado	Q04602	1. Sim	2. Não	(siga Q46c)
c. Praticar atividade física regular	Q04603	1. Sim	2. Não	(siga Q46d)
d. Não fumar	Q04604	1. Sim	2. Não	(siga Q46e)
e. Não beber em excesso	Q04605	1. Sim	2. Não	(siga Q46f)
f. Diminuir o consumo de carboidratos (massas, pães etc.)	Q04606	1. Sim	2. Não	(siga Q46g)
g. Medir a glicemia em casa	Q04607	1. Sim	2. Não	(siga Q46h)
h. Examinar os pés regularmente	Q04608	1. Sim	2. Não	(siga Q46i)
i. Outro	Q04609	1. Sim	2. Não	

(siga Q47)

Q47. Em algum dos atendimentos para diabetes foi pedido algum exame?(Leia as opções de resposta)

a. Exame de sangue (colesterol, glicemia, triglicerídeos)	Q04701	1. Sim	2. Não	(siga Q47b)
b. Hemoglobina glicada	Q04702	1. Sim	2. Não	(siga Q47c)
c. Curva glicêmica	Q04703	1. Sim	2. Não	(siga Q47d)
d. Exame de urina	Q04704	1. Sim	2. Não	(siga Q47e)
e. Outro	Q04705	1. Sim	2. Não	

(Se todos os itens forem = 2, passe ao Q50. Caso contrário, siga Q48.)

Q48. O(A) sr(a) fez todos os exames solicitados? Q048

1. Sim
2. Não

(Se Q48=2, siga Q49. Se Q48=1, passe ao Q50.)

Q49. Qual o principal motivo do(a) sr(a) não ter feito todos os exames solicitados? Q049

01. O exame está marcado, mas ainda não fez
02. Não achou necessário
03. Não conseguiu marcar
04. O tempo de espera no laboratório ou serviço de saúde era muito grande
05. Estava com dificuldades financeiras
06. O laboratório ou serviço de saúde era muito distante
07. O horário de funcionamento do laboratório ou serviço de saúde era incompatível com as suas atividades de trabalho ou domésticas
08. O plano de saúde não cobria todos os exames solicitados
09. Não sabia onde realizar os exames
10. Dificuldade de transporte
11. Outro

(siga Q50)

Q50. Em algum dos atendimentos para diabetes, houve encaminhamento para alguma consulta com médico especialista, tais como cardiologista, endocrinologista, nefrologista ou oftalmologista? Q050

1. Sim
2. Não
3. Não houve encaminhamento, pois todas as consultas para diabetes foram com médico especialista

(Se Q50=1, siga Q51. Se Q50=2 ou 3, passe ao Q53.)

Q51. O(A) sr(a) foi a todas as consultas com médico especialista? Q051

1. Sim
2. Não

(Se Q51=2, siga Q52. Se Q51=1, passe ao Q53.)

Q52. Qual o principal motivo do(a) sr(a) não ter ido a todas as consultas com o médico especialista? Q052

- 01. A consulta está marcada, mas a consulta ainda não foi realizada
- 02. Não achou necessário
- 03. Não sabia quem procurar ou aonde ir
- 04. Estava com dificuldades financeiras
- 05. Teve dificuldades de transporte
- 06. Não conseguiu marcar
- 07. O tempo de espera no serviço de saúde era muito grande
- 08. O plano de saúde não cobria a consulta
- 09. O serviço de saúde era muito distante
- 10. O horário de funcionamento do serviço de saúde era incompatível com as atividades de trabalho ou domésticas
- 11. Outro

(siga Q53)

Q53. Quando foi a última vez que realizaram um exame de vista ou fundo de olho em que dilataram sua pupila?(Leia as opções de resposta) Q053

- 1. Há menos de 6 meses
- 2. Entre 6 meses e menos de 1 ano
- 3. Entre 1 ano e menos de 2 anos
- 4. Entre 2 anos e menos de 3 anos
- 5. Há 3 anos ou mais
- 6. Nunca fez

(siga Q54)

Q54. Quando foi a última vez que um médico ou profissional de saúde examinou seus pés para verificar sensibilidade ou presença de feridas ou irritações? .(Leia as opções de resposta) Q054

- 1. Há menos de 6 meses
- 2. Entre 6 meses e menos de 1 ano
- 3. Entre 1 ano e menos de 2 anos
- 4. Entre 2 anos e menos de 3 anos
- 5. Há 3 anos ou mais
- 6. Nunca teve os pés examinados

(siga Q55)

Q55. O(A) sr(a) tem ou teve alguma destas complicações por causa do diabetes?(Leia as opções de resposta)

a. Problemas na vista	Q05501	1. Sim	2. Não	(siga Q55b)
b. Infarto	Q05502	1. Sim	2. Não	(siga Q55c)
c. AVC (Acidente Vascular cerebral) ou derrame	Q05503	1. Sim	2. Não	(siga Q55d)
d. Outro problema circulatório	Q05504	1. Sim	2. Não	(siga Q55e)
e. Problema nos rins	Q05505	1. Sim	2. Não	(siga Q55f)
f. Úlcera/ferida nos pés	Q05506	1. Sim	2. Não	(siga Q55g)
g. Amputação de membros (pés, pernas, mãos ou braços)	Q05507	1. Sim	2. Não	(siga Q55h)
h. Coma diabético	Q05508	1. Sim	2. Não	(siga Q55i)
i. Outro	Q05509	1. Sim	2. Não	

(siga Q56)

Q56. Alguma vez o(a) sr(a) se internou por causa do diabetes ou de alguma complicação? Q056

- 1. Sim
- 2. Não

(Se Q56=1, siga Q57.
Se Q56=2, passe ao Q58.)

Q57. Há quanto tempo foi a última internação por causa do diabetes ou de alguma complicação? (Leia as opções de resposta) Q057

- 1. Há menos de 6 meses
- 2. Entre 6 meses e menos de 1 ano
- 3. Entre 1 ano e menos de 2 anos
- 4. Entre 2 anos e menos de 3 anos
- 5. Há 3 anos ou mais

(siga Q58)

Q58. Em geral, em que grau o diabetes ou alguma complicação do diabetes limita as suas atividades habituais (*tais como trabalhar, realizar afazeres domésticos, etc.*)?(Leia as opções de resposta) Q058

- 1. Não limita
- 2. Um pouco
- 3. Moderadamente
- 4. Intensamente
- 5. Muito intensamente

(siga Q59)

Q59. Quando foi a última vez que o(a) sr(a) fez exame de sangue para medir o colesterol e triglicerídeos?(Leia as opções de resposta) Q059

- 1. Há menos de 6 meses
- 2. Entre 6 meses e menos de 1 ano
- 3. Entre 1 ano e menos de 2 anos
- 4. Entre 2 anos e menos de 3 anos
- 5. Há 3 anos ou mais
- 6. Nunca fez

(Se Q59=1 ao 5, siga Q60.
Se Q59=6, passe ao Q63.)

Q60. Algum médico já lhe deu o diagnóstico de colesterol alto? Q060

- 1. Sim
- 2. Não

(Se Q60=1, siga Q61. Se Q60=2, passe ao Q63.)

Q61. Que idade o(a) sr(a) tinha no primeiro diagnóstico de colesterol alto?

Q061 __ __ Anos

- 0. Menos de 1 ano

(siga Q62)

Q62. Algum médico ou outro profissional de saúde lhe deu algumas das seguintes recomendações por causa do colesterol alto?(Leia as opções de resposta)

a. Manter uma alimentação saudável (com frutas e vegetais)	Q06201	1. Sim	2. Não	(siga Q62b)
b. Manter o peso adequado	Q06202	1. Sim	2. Não	(siga Q62c)
c. Prática de atividade física	Q06203	1. Sim	2. Não	(siga Q62d)
d. Tomar medicamentos	Q06204	1. Sim	2. Não	(siga Q62e)
e. Não fumar	Q06205	1. Sim	2. Não	(siga Q62f)
f. Fazer acompanhamento regular	Q06206	1. Sim	2. Não	

(siga Q63)

Q63. Algum médico já lhe deu o diagnóstico de uma doença do coração, tais como infarto, angina, insuficiência cardíaca ou outra?(Leia as opções de resposta)

Q063 1. Sim 2. Não

(Se Q63= 2, passe ao Q68. Caso contrário, siga para os itens abaixo.)

a. Infarto	Q06301	1. Sim	2. Não	(siga Q63b)
b. Angina	Q06302	1. Sim	2. Não	(siga Q63c)
c.Insuficiência cardíaca	Q06303	1. Sim	2. Não	(siga Q63d)
d. Outra	Q06304	1. Sim	2. Não	

(Se todas = 2, passe ao Q68. Caso contrário, siga Q64.)

Q64. Que idade o(a) sr(a) tinha no primeiro diagnóstico da doença do coração?

Q064 __ __ Anos 0. Menos de 1 ano (siga Q65)

Q65. O que o(a) sr(a) faz atualmente por causa da doença do coração?
(Leia as opções de resposta)

a. Dieta Q06501 ☐ 1. Sim ☐ 2. Não (siga Q65b)

b. Prática de atividade física Q06502 1. Sim ☐ 2. Não (siga Q65c)

c. Toma medicamentos Q06503 1. Sim ☐ 2. Não (siga Q65d)

d. Outro Q06504 1. Sim ☐ 2. Não
(siga Q66)

Q66. O(A) sr(a) já fez alguma cirurgia de ponte de safena ou colocação de stent ou angioplastia? Q066

☐ 1. Sim ☐ 2. Não

(siga Q67)

Q67. Em geral, em que grau a doença do coração limita as suas atividades habituais (*tais como trabalhar, realizar afazeres domésticos, etc.*)? Q067
(Leia as opções de resposta)

☐ 1. Não limita ☐ 3. Moderadamente ☐ 5. Muito intensamente

☐ 2. Um pouco ☐ 4. Intensamente

(siga Q68)

Q68. Algum médico já lhe deu o diagnóstico de AVC (Acidente Vascular cerebral) ou derrame? Q068

☐ 1. Sim ☐ 2. Não

(Se Q68=2, passe ao Q74.
Se Q68=1, siga Q69.)

Q69. Quantos derrames (ou AVC) o(a) sr(a) já teve?

Q069
Quantos

(siga Q70)

Q70. Que idade o(a) sr(a) tinha no primeiro diagnóstico do derrame (ou AVC)?

Q070 ☐ 0. Menos de 1 ano

Anos

(siga Q71)

Q71. Por causa do derrame (ou AVC), o(a) sr(a) realizou tomografia ou ressonância da cabeça?

☐ 1. Sim Q071 ☐ 2. Não

(siga Q72)

Q72. O que o(a) sr(a) faz atualmente por causa do derrame (ou AVC)? (Leia as opções de resposta)

a. Dieta Q07201 ☐ 1. Sim ☐ 2. Não (siga Q72b)

b. Fisioterapia Q07202 ☐ 1. Sim ☐ 2. Não (siga Q72c)

c. Outras terapias de reabilitação Q07203 1. Sim ☐ 2. Não (siga Q72d)

d. Toma aspirina Q07204 ☐ 1. Sim ☐ 2. Não (siga Q72e)

e. Toma outros medicamentos Q07205 1. Sim ☐ 2. Não (siga Q72f)

d. Outro Q07206 ☐ 1. Sim ☐ 2. Não
(siga Q73)

Q73. Em geral, em que grau o derrame (ou AVC) limita as suas atividades habituais (*tais como trabalhar, realizar afazeres domésticos, etc.*)? Q073
(Leia as opções de resposta)

☐ 1. Não limita ☐ 4. Intensamente

☐ 2. Um pouco ☐ 5. Muito intensamente

☐ 3. Moderadamente

(siga Q74)

Q74. Algum médico já lhe deu o diagnóstico de asma (ou bronquite asmática)? Q074

☐ 1. Sim

☐ 2. Não

(Se Q74=1, siga Q75.
Se Q74=2, passe ao Q79.)

Q75. Que idade o(a) sr(a) tinha no primeiro diagnóstico de asma?

Q075 ☐ 0. Menos de 1 ano

Idade

(siga Q76)

Q76. Nos últimos 12 meses, o(a) sr(a) teve alguma crise de asma? Q076

☐ 1. Sim

☐ 2. Não

(Se Q76=1, siga Q77.
Se Q76=2, passe ao Q79.)

Q77. O que o(a) sr(a) faz atualmente por causa da asma?

a. Usa medicamentos (inaladores, aerossol ou comprimidos) Q07701 1. Sim ☐ 2. Não (siga Q77a)

b. Outro Q07702 ☐ 1. Sim ☐ 2. Não
(siga Q78)

Q78. Em geral, em que grau a asma limita as suas atividades habituais (*tais como trabalhar, realizar afazeres domésticos, etc.*)? Q078
(Leia as opções de resposta)

☐ 1. Não limita ☐ 4. Intensamente

☐ 2. Um pouco ☐ 5. Muito intensamente

☐ 3. Moderadamente

(siga Q79)

Q79. Algum médico já lhe deu o diagnóstico de artrite ou reumatismo? Q079

☐ 1. Sim

☐ 2. Não

(Se Q79=1, siga Q80.
Se Q79=2, passe ao Q84.)

Q80. Que idade o(a) sr(a) tinha no primeiro diagnóstico de artrite ou reumatismo?

Q080 ☐ 0. Menos de 1 ano

Anos

(siga Q81)

Q81. O que o(a) sr(a) faz atualmente por causa da artrite ou reumatismo? (Leia as opções de resposta)

a. Exercício ou atividade física Q08101 1. Sim ☐ 2. Não (siga Q81b)

b. Fisioterapia Q08102 ☐ 1. Sim ☐ 2. Não (siga Q81c)

c. Usa medicamentos ou injeções Q08103 Sim ☐ 2. Não (siga Q81d)

d. Faz acupuntura Q08104 ☐ 1. Sim ☐ 2. Não (siga Q81e)

e. Outro Q08105 ☐ 1. Sim ☐ 2. Não

(siga Q82)

Q82. O(A) sr(a) já fez alguma cirurgia por causa da artrite ou reumatismo? Q082

☐ 1. Sim

☐ 2. Não

(siga Q83)

Q83. Em geral, em que grau a artrite ou reumatismo limita as suas atividades habituais (*tais como trabalhar, realizar afazeres domésticos, etc.*)? Q083
(Leia as opções de resposta)

☐ 1. Não limita ☐ 4. Intensamente

☐ 2. Um pouco ☐ 5. Muito intensamente

☐ 3. Moderadamente

(siga Q84)

Q84. O(a) sr(a) tem algum problema crônico de coluna, como dor crônica nas costas ou no pescoço, lombalgia, dor ciática, problemas nas vértebras ou disco?

☐ 1. Sim Q084

☐ 2. Não

(Se Q84=1, siga ao Q85.
Se Q84=2, passe ao Q88.)

Q85. Que idade o(a) sr(a) tinha quando começou o problema na coluna?

Q085 ☐ 0. Menos de 1 ano

Anos

(siga Q86)

Q86. O que o(a) sr(a) faz atualmente por causa do problema na coluna? (Leia as opções de resposta)

a. Exercício ou fisioterapia Q08601 1. Sim 2. Não (siga Q86b)

b. Usa medicamentos ou injeções Q08603 1. Sim 2. Não (siga Q86c)

c. Faz acupuntura Q08604 1. Sim 2. Não (siga Q86d)

d. Outro Q08605 1. Sim 2. Não

(siga Q87)

Q87. Em geral, em que grau o problema na coluna limita as suas atividades habituais (*tais como trabalhar, realizar afazeres domésticos, etc.*)?(Leia as opções de resposta) Q087

1. Não limita
2. Um pouco
3. Moderadamente
4. Intensamente
5. Muito intensamente

(siga Q88)

Q88. Algum médico já lhe deu o diagnóstico de DORT (distúrbio osteomuscular relacionado ao trabalho)? Q088

1. Sim
2. Não

(Se Q88=1, siga Q89.
Se Q88=2, passe ao Q92.)

Q89. Que idade o(a) sr(a) tinha no primeiro diagnóstico de DORT? Q089

Anos

0. Menos de 1 ano

(siga Q90)

Q90. O que o(a) sr(a) faz atualmente por causa do DORT?(Leia as opções de resposta)

a. Exercício ou fisioterapia Q09001 1. Sim 2. Não (siga Q90b)

b. Usa medicamentos ou injeções Q09003 1. Sim 2. Não (siga Q90c)

c. Faz acupuntura Q09004 1. Sim 2. Não (siga Q90d)

d. Outro Q09005 1. Sim 2. Não

(siga Q91)

Q91. Em geral, em que grau o DORT limita as suas atividades habituais (*tais como trabalhar, realizar afazeres domésticos, etc.*)? (Leia as opções de resposta) Q091

1. Não limita
2. Um pouco
3. Moderadamente
4. Intensamente
5. Muito intensamente

(siga Q92)

Q92. Algum médico ou profissional de saúde mental (como psiquiatra ou psicólogo) já lhe deu o diagnóstico de depressão? Q092

1. Sim
2. Não

(Se Q92=1, siga Q93.
Se Q92=2, passe ao Q110.)

Q93. Que idade o(a) sr(a) tinha no primeiro diagnóstico de depressão? Q093

Anos

0. Menos de 1 ano

(siga Q94)

Q94. O(A) sr(a) vai ao médico/serviço de saúde regularmente por causa da depressão? Q094

1. Sim
2. Não, só quando tem algum problema
3. Nunca vai

(Se Q94=1, passe ao Q96.
Se Q94=2 ou 3, siga Q95.)

Q95. Qual o principal motivo do(a) sr(a) não visitar o médico/serviço de saúde regularmente por causa da depressão? Q095

01. Não está mais deprimido
02. O serviço de saúde é muito distante
03. Não tem ânimo
04. O tempo de espera no serviço de saúde é muito grande
05. Tem dificuldades financeiras
06. O horário de funcionamento do serviço de saúde é incompatível e com suas atividades de trabalho ou domésticas
07. O plano de saúde não cobre as consultas
08. Não sabe quem procurar ou aonde ir
09. Dificuldade de transporte
10. Outro

(siga Q96)

Q96. Quais tratamentos o(a) sr(a) faz atualmente por causa da depressão? (Leia as opções de resposta)

a. Faz psicoterapia Q09601 1. Sim 2. Não (siga Q96b)

b. Toma medicamentos Q09602 1. Sim 2. Não (siga Q96c)

c. Outro Q09603 1. Sim 2. Não

(Se Q96b = 2, passe ao Q101. Caso contrário, siga Q97.)

Q97. Algum dos medicamentos para depressão foi coberto por plano de saúde? (Leia as opções de resposta) Q097

1. Sim, todos
2. Sim, alguns
3. Não, nenhum

(Se Q97=1, passe ao Q100.
Se Q97=2 ou 3, siga Q98.)

Q98. Algum dos medicamentos para depressão foi obtido em serviço público de saúde?(Leia as opções de resposta) Q098

1. Sim, todos
2. Sim, alguns
3. Não, nenhum

(siga Q100)

Q100. O(A) sr(a) pagou algum valor pelos medicamentos? Q100

1. Sim
2. Não

(siga Q101)

Q101. Quando foi a última vez que o(a) sr(a) recebeu assistência médica por causa da depressão? (Leia as opções de resposta) Q101

1. Há menos de 6 meses
2. Entre 6 meses e menos de 1 ano
3. Entre 1 ano e menos de 2 anos
4. Entre 2 anos e menos de 3 anos
5. Há 3 anos ou mais
6. Nunca recebeu

(Se Q101=1 ao 5, siga ao Q102. Se Q101=6, passe ao Q109.)

Q102. Na última vez que recebeu assistência médica para depressão, onde o(a) sr(a) foi atendido? Q0102

- 1. Unidade básica de saúde (posto ou centro de saúde ou unidade de saúde da família)
- 2. Centro de Especialidades, Policlínica pública ou PAM – Posto de Assistência Médica
- 3. UPA (Unidade de Pronto Atendimento)
- 04. CAPS – Centro de Atenção Psicossocial
- 05. Outro tipo de Pronto Atendimento Público (24 horas)
- 06. Pronto-socorro ou emergência de hospital público
- 07. Hospital público/ambulatório
- 08. Consultório particular ou clínica privada
- 09. Ambulatório ou consultório de empresa ou sindicato
- 10. Pronto-atendimento ou emergência de hospital privado
- 11. No domicílio, com médico da equipe de saúde da família.
- 12. No domicílio, com médico particular
- 13. Outro

(siga Q103)

Q103. Esse atendimento foi coberto por plano de saúde? Q0103

- 1. Sim
- 2. Não

(siga Q104)

Q104. O(A) sr(a) pagou algum valor por esse atendimento?(Entrevistador: Se *o(a) e entrevistado(a) responder que pagou, mas teve reembolso total, marque a opção 2)* Q0104

- 1. Sim
- 2. Não

(siga Q105)

Q105. Esse atendimento foi feito pelo SUS? Q0105

- 1. Sim
- 2. Não
- 3. Não sabe

(siga Q106)

Q106. Em algum dos atendimentos para depressão, houve encaminhamento para algum acompanhamento com profissional de saúde mental, como psiquiatra ou psicólogo? Q0106

- 1. Sim
- 2. Não
- 3. Não houve encaminhamento, pois todas as consultas para depressão foram com profissional de saúde mental

(Se Q106=1, siga Q107. Se Q106=2 ou 3, passe ao Q109.)

Q107. O(A) sr(a) conseguiu ir a todas as consultas com profissional especialista de saúde mental? Q0107

- 1. Sim
- 2. Não

(Se Q107=1, passe ao Q109. Se Q107=2, siga Q108.)

Q108. Qual o principal motivo do(a) sr(a) não ter ido a todas as consultas com o profissional especialista de saúde mental? Q0108

- 01. A consulta está marcada, mas a consulta ainda não foi realizada
- 02. Não conseguiu marcar
- 03. Não achou necessário
- 04. Não teve ânimo
- 05. O tempo de espera no serviço de saúde era muito grande
- 06. Não sabia quem procurar ou aonde ir
- 07. Estava com dificuldades financeiras
- 08. Teve dificuldades de transporte
- 09. O plano de saúde não cobria a consulta
- 10. O serviço de saúde era muito distante
- 11. O horário de funcionamento do serviço de saúde era incompatível com as atividades de trabalho ou domésticas
- 12. Outro

(siga Q109)

Q109. Em geral, em que grau a depressão limita as suas atividades habituais (tais como trabalhar, realizar afazeres domésticos, etc.)? (Leia as opções de resposta) Q0109

- 1. Não limita
- 2. Um pouco
- 3. Moderadamente
- 4. Intensamente
- 5. Muito intensamente

(siga Q110)

Q110. Algum médico ou profissional de saúde mental (como psiquiatra ou psicólogo) já lhe deu o diagnóstico de outra doença mental, como esquizofrenia, transtorno bipolar, psicose ou TOC (Transtorno Obsessivo Compulsivo)? Q0110

- 1. Sim
- 2. Não

(Se Q110= 2, passe ao Q116. Caso contrário, siga para os itens abaixo.)

a. Esquizofrenia	Q11001	1. Sim	2. Não	(siga Q110b)
b. Transtorno bipolar	Q11002	1. Sim	2. Não	(siga Q110c)
c. TOC (Transtorno obsessivo compulsivo)	Q11003	1. Sim	2. Não	(siga Q110d)
d. Outro	Q11004	1. Sim	2. Não	

(Se todas = 2, passe ao Q116. Caso contrário, siga Q111.)

Q111. Que idade o(a) sr(a) tinha no primeiro diagnóstico de doença mental?

Q111

__ __ Anos

0. Menos de 1 ano

(siga Q112)

Q112. O(A) sr(a) visita o médico/serviço de saúde regularmente por causa dessa doença mental? Q112

1. Sim
2. Não
3. Não, só quando tenho algum problema

(Se Q112=1, passe ao Q114. Se Q112=2 ou 3, siga Q113.)

Q113. Qual o principal motivo do(a) sr(a) não visitar o médico/serviço de saúde regularmente? Q113

1. Não acha necessário
2. O serviço de saúde é muito distante
3. Acha que não vai ser bem recebido no serviço de saúde porque tem uma doença mental
4. Tem dificuldades financeiras
5. O horário de funcionamento do serviço de saúde é incompatível com suas atividades de trabalho ou domésticas
6. O plano de saúde não cobre as consultas regulares
7. Não sabe quem procurar ou aonde ir
8. Dificuldade de transporte
9. Outro

(siga Q114)

Q114. Quais tratamentos o(a) sr(a) faz atualmente por causa da doença mental?(Leia as opções de resposta)

a. Faz psicoterapia Q11401 — 1. Sim — 2. Não (siga Q114b)

b. Usa medicamentos ou injeções Q11402 — 1. Sim — 2. Não (siga Q114c)

c. Outro Q11403 — 1. Sim — 2. Não

(siga Q115)

Q115. Em geral, em que grau essa doença mental limita as suas atividades habituais (*tais como trabalhar, realizar afazeres domésticos etc.*)?(Leia as opções de resposta)

Q115

1. Não limita
2. Um pouco
3. Moderadamente
4. Intensamente
5. Muito intensamente

(siga Q116)

Q116. Algum médico já lhe deu o diagnóstico de alguma doença no pulmão, tais como enfisema pulmonar, bronquite crônica ou DPOC (Doença Pulmonar Obstrutiva Crônica)?

Q116

1. Sim
2. Não

(Se Q116= 2, passe ao Q120. Caso contrário, siga para os itens abaixo.)

a. Enfisema pulmonar Q11601 — 1. Sim — 2. Não (siga Q116b)

b. Bronquite crônica Q11602 — 1. Sim — 2. Não (siga Q116c)

c. Outra Q11603 — 1. Sim — 2. Não

(Se todas = 2, passe ao Q120. Caso contrário, siga Q117.)

Q117. Que idade o(a) sr(a) tinha no primeiro diagnóstico da doença no pulmão?

Q117

__ __ Anos

0. Menos de 1 ano

(siga Q118)

Q118. O que o(a) sr(a) faz atualmente por causa da doença no pulmão?(Leia as opções de resposta)

a. Usa medicamentos (inaladores, aerossol ou comprimidos) Q11801 — 1. Sim — 2. Não (siga Q118b)

b. Usa oxigênio Q11802 — 1. Sim — 2. Não (siga Q118c)

c. Fisioterapia respiratória Q11803 — 1. Sim — 2. Não (siga Q118d)

d. Outro Q11804 — 1. Sim — 2. Não

(siga Q119)

Q119. Em geral, em que grau a doença do pulmão limita as suas atividades habituais (*tais como trabalhar, realizar afazeres domésticos etc.*)?
(Leia as opções de resposta)

Q119

1. Não limita
2. Um pouco
3. Moderadamente
4. Intensamente
5. Muito intensamente

(siga Q120)

Q120. Algum médico já lhe deu algum diagnóstico de câncer? Q120

1. Sim
2. Não

(Se Q120=1, siga Q121.
Se Q120=2, passe ao Q124.)

Q121. No primeiro diagnóstico de câncer, que tipo de câncer o(a) sr(a) tem ou teve?
(Leia as opções de resposta) Q121

1. Pulmão
2. Intestino
3. Estômago
4. Mama (*só para mulheres*)
5. Colo de útero (*só para mulheres*)
6. Próstata (*só para homens*)
7. Pele
8. Outro

(siga Q122)

Q122. Que idade o(a) sr(a) tinha no primeiro diagnóstico de câncer?

Q122

Anos

0. Menos de 1 ano

(siga Q123)

Q123. Em geral, em que grau o câncer ou algum problema provocado pelo câncer limita as suas atividades habituais (*tais como trabalhar, realizar afazeres domésticos, etc.*)?
(Leia as opções de resposta) Q123

1. Não limita
2. Um pouco
3. Moderadamente
4. Intensamente
5. Muito intensamente

(siga Q124)

Q124. Algum médico já lhe deu o diagnóstico de insuficiência renal crônica? Q124

1. Sim
2. Não

(Se Q124=1, siga Q125.
Se Q124=2, passe ao Q128.)

Q125. Que idade o(a) sr(a) tinha no primeiro diagnóstico de insuficiência renal crônica?

Q125

Anos

0. Menos de 1 ano

(siga Q126)

Q126. O que o(a) sr(a) faz ou fez por causa da insuficiência renal crônica?(Leia as opções de resposta)

a. Toma medicamentos Q12601 1. Sim 2. Não (siga Q126b)

b. Hemodiálise Q12602 1. Sim 2. Não (siga Q126c)

c. Diálise peritoneal Q12603 1. Sim 2. Não (siga Q126d)

d. Fez transplante de rim Q12604 1. Sim 2. Não (siga Q126e)

e. Outro Q12605 1. Sim 2. Não

(siga Q127)

Q127. Em geral, em que grau a insuficiência renal crônica limita as suas atividades habituais (*tais como trabalhar, realizar afazeres domésticos, etc.)?*
Leia as opções de resposta) Q127

1. Não limita
2. Um pouco
3. Moderadamente
4. Intensamente
5. Muito intensamente

(siga Q128)

Q128. Algum médico já lhe deu algum diagnóstico de outra doença crônica, física ou mental, ou doença de longa duração (de mais de 6 meses de duração)?

Q128

1. Sim
2. Não

(Se Q128=1, siga Q130. Se Q128=2, passe ao Q132.)

Q130. Que idade o(a) sr(a) tinha no primeiro diagnóstico?

Q130

Anos

0. Menos de 1 ano

(siga Q131)

Q131. Em geral, em que grau esta doença limita suas atividades habituais (*tais como trabalhar, realizar afazeres domésticos, etc.*)?
(Leia as opções de resposta)

1. Não limita Q131
2. Um pouco
3. Moderadamente
4. Intensamente
5. Muito intensamente

(siga Q132)

Q132. Nas últimas duas semanas, o(a) sr(a) fez uso de algum medicamento para dormir? Q132

1. Sim
2. Não

(Se Q132=1, siga Q133.
Se Q132=2 e homem com 40 anos ou mais, passe ao Q136. Se Q132=2 e homem com menos de 40 anos, passe ao módulo U. Se Q132=2 e mulher, passe ao módulo R.)

Q133. Nas últimas duas semanas, por quantos dias usou o medicamento para dormir?

Q133

Dias

(siga Q134)

Q134. O medicamento que o(a) sr(a) usa para dormir foi receitado por médico? Q134

1. Sim
2. Não

(Se Q134=1, siga Q135.
Se Q134=2 e homem com 40 anos ou mais, passe ao Q136.
Se Q134=2 e homem com menos de 40 anos, passe ao módulo U. Se Q134=2 e mulher, passe ao módulo R.)

Q135. Foi receitado para o(a) sr(a) mesmo(a)? Q135

1. Sim
2. Não, foi receitado por médico para outra pessoa

(Se homem com 40 anos ou mais de idade, siga Q136. Se Homem com menos de 40 anos, passe ao módulo U. Se mulher, passe ao módulo R.)

Q136. Quando foi a última vez que o sr fez um exame físico/toque retal da próstata?(Leia as opções de resposta) Q136

1. Menos de 1 ano atrás
2. De 1 ano a menos de 2 anos
3. De 2 anos a menos de 3 anos
4. 3 anos ou mais atrás
5. Nunca fez

(Se Q136= 1 a 4, passe ao módulo U. Se Q136=5, siga Q137.)

Q137.Qual o principal motivo do sr nunca ter feito o exame? Q137

01. Não acha necessário
02. Tem vergonha
03. Nunca foi orientado para fazer o exame
04. Não sabe quem procurar ou aonde ir
05. Tem dificuldades financeiras
06. Tem dificuldades de transporte
07. Teve dificuldades para marcar consulta
08. O tempo de espera no serviço de saúde é muito grande
09. O serviço de saúde é muito distante
10. O horário de funcionamento do serviço é incompatível com suas atividades de trabalho ou habituais
11.O plano de saúde não cobre a consulta
12. Está marcado, mas ainda não realizou
13. Outro

(Se homem, passe ao Módulo U.) (Se mulher ir para Módulo R.)

Módulo R. Saúde da Mulher (mulheres de 18 anos e mais de idade)

Neste módulo, vamos fazer perguntas sobre a sua saúde, exames preventivos, história reprodutiva e planejamento familiar.

R1. Quando foi a última vez que a sra fez um exame preventivo para câncer de colo do útero? (Leia as opções de resposta) R001

- 1. Menos de 1 ano atrás
- 2. De 1 ano a menos de 2 anos
- 3. De 2 anos a menos de 3 anos
- 4. 3 anos ou mais atrás
- 5. Nunca fez

(Se R1 = 1 a 4, passe ao R3. Se R1 = 5, siga R2.)

R2. Qual o principal motivo da sra nunca ter feito um exame preventivo? R002

- 01. Nunca teve relações sexuais
- 02. Não acha necessário
- 03. Tem vergonha
- 04. Nunca foi orientada para fazer o exame
- 05. Não sabe quem procurar ou aonde ir
- 06. Tem dificuldades financeiras
- 07. Tem dificuldades de transporte
- 08. Teve dificuldades para marcar consulta
- 09. O tempo de espera no serviço de saúde é muito grande
- 10. O serviço de saúde é muito distante
- 11. O horário de funcionamento do serviço é incompatível com suas atividades de trabalho ou domésticas
- 12. O plano de saúde não cobre a consulta
- 13. Está marcado, mas ainda não realizou
- 14. Outro

(passe ao R10)

R3. O último exame preventivo para câncer do colo do útero foi coberto por algum plano de saúde? R003

- 1. Sim
- 2. Não

(siga R4)

R4. A sra pagou algum valor pelo último exame preventivo para câncer do colo do útero? R004

***(Entrevistador:** Se a entrevistada responder que pagou, mas teve reembolso, marque a opção 2)*

- 1. Sim
- 2. Não

(siga R5)

R5. O último exame preventivo para câncer do colo do útero foi feito através do Sistema Único de Saúde (SUS)? R005

- 1. Sim
- 2. Não
- 3. Não sabe

(siga R6)

R6. Quando a sra recebeu o resultado do último exame preventivo? (Leia as opções de resposta) R006

- 1. Menos de 1 mês depois
- 2. Entre 1 mês e menos de 3 meses depois
- 3.Entre 3 meses e menos de 6 meses depois
- 4. 6 meses ou mais depois
- 5. Ainda não recebi
- 6. Nunca recebi
- 7. Nunca fui buscar

(Se R6 = 1 ao 4, siga R7.
Se R6 = 5, 6 ou 7, passe ao R10.)

R7.Após receber o resultado do exame, a sra foi encaminhada a alguma consulta com ginecologista ou outro médico especialista? R007

- 1. Sim
- 2. Não
- 3. Não houve encaminhamento, pois todas as minhas consultas por este motivo foram com médico especialista

(Se R7 = 1, siga R8.
Se R7 = 2 ou 3, passe ao R10.)

R8. A sra foi à consulta? R008

- 1. Sim
- 2. Não

(Se R8 = 2, siga R9.
Se R8 = 1, passe ao R10.)

R9. Qual o principal motivo da sra não ter ido à consulta? R009

- 01. A consulta está marcada, mas ainda não foi à consulta
- 02. Não achou necessário
- 03. Não sabia quem procurar ou aonde ir
- 04. Estava com dificuldades financeiras
- 05. Teve dificuldades de transporte
- 06. Não conseguiu marcar
- 07. O tempo de espera no serviço de saúde era muito grande
- 08. O plano de saúde não cobria a consulta
- 09.O serviço de saúde era muito distante
- 10.O horário de funcionamento do serviço de saúde era incompatível com as atividades de trabalho ou domésticas
- 11. Outro

(siga R10)

R10. A sra já foi submetida a cirurgia para retirada do útero? R010

- 1. Sim
- 2. Não

(Se R10=1, siga R11.
Se R10=2, passe ao R13.)

R11. Segundo o médico, qual o motivo da retirada do útero?(Leia as opções de resposta) R011

- 1. Mioma uterino
- 2. Prolapso do útero (útero caído)
- 3. Endometriose
- 4. Câncer ginecológico
- 5. Complicações da gravidez ou parto
- 6. Sangramento vaginal anormal
- 7. Outro

(siga R12)

R12. Que idade a sra tinha quando foi submetida à cirurgia? R012

|__|__| Anos

(siga R13)

R13. Quando foi a última vez que um médico ou enfermeiro fez o exame clínico das suas mamas? (Leia as opções de resposta) R013

- 1. Menos de 1 ano atrás
- 2. De 1 ano a menos de 2 anos atrás
- 3. De 2 anos a menos de 3 anos atrás
- 4. 3 anos ou mais atrás
- 5. Nunca fez

(siga R14)

R14. Algum médico já lhe solicitou um exame de mamografia? R014

- 1. Sim
- 2. Não

(Se R14=1, siga R15.
Se R14=2, passe ao R25.)

R15. A sra fez o exame de mamografia? R015

- 1. Sim
- 2. Não

(Se R15=1, passe ao R17.
Se R15=2, siga R16.)

R16. Qual o principal motivo da sra não ter feito o exame de mamografia? R016

- 01. O exame está marcado, mas ainda não fez o exame
- 02. Não achou necessário
- 03. Não sabia onde realizar o exame
- 04. Não conseguiu marcar
- 05. Estava com dificuldades financeiras
- 06. Teve dificuldades de transporte
- 07. O tempo de espera no serviço de saúde era muito grande
- 08. O serviço de saúde era muito distante
- 09.O horário de funcionamento do serviço de saúde era incompatível com as suas atividades de trabalho ou domésticas
- 10. O plano de saúde não cobria a mamografia
- 11. Outro

(passe ao R25)

R17. Quando foi a última vez que a sra fez um exame de mamografia?
(Leia as opções de resposta) R017

- 1. Menos de 1 ano atrás
- 2. De 1 ano a menos de 2 anos
- 3. De 2 anos a menos de 3 anos
- 4. 3 anos ou mais atrás

(siga R18)

R18. A última mamografia foi coberta por algum plano de saúde? R018

- 1. Sim
- 2. Não

(siga R19)

R19. A sra pagou algum valor pela última mamografia?

(***Entrevistador**: Se a entrevistada responder que pagou, mas teve reembolso, marque a opção 2)*

R019

- 1. Sim
- 2. Não

(siga R20)

R20. A última mamografia foi feita através do Sistema Único de Saúde (SUS)? R020

- 1. Sim
- 2. Não
- 3. Não sabe

(siga R21)

R21. Quando a sra recebeu o resultado do exame de mamografia?
(Leia as opções de resposta) R021

- 1. Menos de 1 mês depois
- 2. Entre 1 mês e menos de 3 meses depois
- 3. Entre 3 meses e menos de 6 meses depois
- 4. 6 meses ou mais ou mais depois
- 5. Ainda não recebi
- 6. Nunca recebi
- 7. Nunca fui buscar

(Se R21=1 a 4, siga R22. Se R21=5, 6 ou 7, passe ao R25.)

R22. Após receber o resultado da mamografia, a sra foi encaminhada para consulta com médico especialista? R022

- 1. Sim
- 2. Não
- 3. Não houve encaminhamento, pois todas as minhas consultas por este motivo foram com médico especialista

(Se R22=1, siga R23.
Se R22=2 ou 3, passe ao R25.)

R23. A sra foi à consulta com o especialista? R023

- 1. Sim
- 2. Não

(Se R23=1, passe ao R25.
Se R23=2, siga R24.)

R24. Qual o principal motivo da sra não ter ido à consulta com o especialista? R024

- 01. A consulta está marcada, mas ainda não foi à consulta
- 02. Não conseguiu marcar
- 03. Não achou necessário
- 04. Não sabia quem procurar ou aonde ir
- 05. Estava com dificuldades financeiras
- 06. Teve dificuldades de transporte
- 07. O tempo de espera no serviço de saúde era muito grande
- 08. O plano de saúde não cobria a consulta
- 09.O serviço de saúde era muito distante
- 10. O horário de funcionamento do serviço de saúde era incompatível com as atividades de trabalho ou domésticas
- 11. Outro

(siga ao R25)

Agora vou lhe fazer perguntas sobre a menstruação e a menopausa.

R25. Com que idade a sra ficou menstruada pela primeira vez?

R025

|__|__| Anos

- 00. Não sabe

(Se R10=1, passe ao R39.
Se R10=2 , siga R26.)

R26. A sra ainda fica menstruada? R026

- 1. Sim
- 2. Não

(Se R26=1, passe ao R31.
Se R26=2, siga R27.)

R27. Com que idade a sra parou de menstruar?

R027

|__|__| Anos

- 00. Não sabe

(siga R28)

R28. A sra já entrou na menopausa? R028

- 1. Sim
- 2. Não
- 3. Não sei

(Se R28=1, siga R29.
Se R28=2 ou 3, passe ao R39.)

R29. Alguma vez a sra fez ou faz tratamento hormonal para alívio dos sintomas da menopausa (com comprimidos, adesivos, gel ou injeções)? R029

- 1. Sim, faz atualmente
- 2. Sim, já fez mas não faz mais
- 3. Não, nunca fez

(Se R29=1 ou 2, siga R30. Se R29=3, passe ao R39.)

R30. Este medicamento foi receitado por médico? R030

- 1. Sim
- 2. Não

(passe ao R39)

Agora vou lhe fazer perguntas sobre planejamento familiar e contracepção.

R31. Nos últimos 12 meses, a sra teve relações sexuais? R031

- 1. Sim
- 2. Não

(Se R31=1, siga R32.
Se R31=2, passe ao R39.)

R32. Nos últimos 12 meses, a sra participou de grupo de planejamento familiar? R032

- 1. Sim
- 2. Não

(Se R32=1, siga R33.
Se R32=2, passe ao R34.)

R33. E o seu parceiro participou de grupo de planejamento familiar? R033

- 1. Sim
- 2. Não

(siga R34)

R34. A sra usa algum método para evitar a gravidez atualmente? R034

- 1. Sim
- 2. Não

(Se R34=2, siga R35.
Se R34=1, passe ao R36.)

R35. Qual o principal motivo de não evitar a gravidez? R035

1. Quer engravidar ou não se incomoda de engravidar
2. Por motivos religiosos
3. Não sabe como evitar
4. Não sabe aonde ir ou quem procurar para lhe dar orientações
5. Está grávida
6. Ligou as trompas
7. O companheiro fez vasectomia
8. Não tem relações sexuais com homens
9. Outro

(passe ao R37)

R36. Que método para evitar a gravidez a sra usa atualmente?(leia as opções de resposta)

a. Pílula	R03601	1. Sim	2. Não	(siga R36b)
b. Tabela	R03602	1. Sim	2. Não	(siga R36c)
c. Camisinha masculina	R03603	1. Sim	2. Não	(siga R36d)
d. Camisinha feminina	R03604	1. Sim	2. Não	(siga R36e)
e. Diafragma	R03605	1. Sim	2. Não	(siga R36f)
f. DIU	R03606	1. Sim	2. Não	(siga R36g)
g. Contraceptivo Injetável	R03607	1. Sim	2. Não	(siga R36h)
h. Implantes (Norplant)	R03608	1. Sim	2. Não	(siga R36i)
i. Creme/óvulo	R03609	1. Sim	2. Não	(siga R36j)
j. Pílula do dia seguinte (Contracepção de emergência)	R03610	1. Sim	2. Não	(siga R36k)
k. Outro	R03611	1. Sim	2. Não	

(siga R37)

R37. A sra e/ou seu companheiro já fizeram ou fazem algum tratamento para engravidar? R037

1. Sim, fazem atualmente
2. Sim, já fizeram
3. Nunca fizeram

(Se R37=1, siga R38. Se R37=2 ou 3, passe ao R39.)

R38. Há quanto tempo a sra está tentando engravidar?(leia as opções de resposta) R038

1. Há menos de 6 meses
2. De 6 meses a menos de 1 ano
3. Há 1 ano ou mais

(siga R39)

Agora vou lhe fazer perguntas sobre história reprodutiva.
(Se mulher e C8 ≥ 50 ou mais → passe ao Módulo U. Se mulher e C8< 50, siga R39).

R39. Durante a sua vida, a sra já ficou grávida (mesmo que a gravidez não tenha chegado até o final)? R039

1. Sim
2. Não

(Se R39=1, siga R40.
Se R39=2, passe ao módulo U.)

R40. Com que idade a sra teve a sua primeira gravidez? R040

Anos

00. Não sabe

(siga R41)

R41. A sra já teve algum aborto espontâneo? R041

1. Sim R04101 Quantos
2. Não

(siga R42)

R42. A sra já teve algum aborto provocado? R042

1. Sim R04201 Quantos
2. Não

(siga R43)

R43. Quantos partos a sra já teve? R043

Quantos

00. Nenhum

(Se R43 = 00, passe ao Módulo U.
Caso contrário, siga R44.)

R44. Quantos partos foram cesarianas? R044

Quantos

00. Nenhum

(siga R45)

R45. Quantos filhos nasceram vivos (ou seja, que apresentaram algum sinal de vida ao nascer)? R045

Filhos vivos

(siga R46)

R46. Destes filhos nascidos vivos, quantos já morreram? R046

Quantos

00. Nenhum

(siga R47)

R47. Destes filhos nascidos vivos, quantos nasceram com peso menor que dois quilos e meio? R047

Quantos

00. Nenhum

(siga R48)

R48. Destes filhos nascidos vivos, quantos nasceram antes do tempo, isto é antes de completar 9 meses de gestação? R048

Quantos

00. Nenhum

(siga R49)

R49. Em que data foi o último parto?

R04901 / R04902 / R04903

Dia / Mês / Ano

Se posterior a ___/__/____ ir para o Módulo S.
Se anterior a __/__/____ ir para o Módulo U.
(Encerre o módulo)

Módulo S. Atendimento Pré-natal

Agora vou lhe fazer perguntas sobre o atendimento pré-natal.

***Entrevistador**: As questões deste módulo são dirigidas às mulheres que tiveram algum parto no período de 28/07/2011 a 27/07/2013. Considerar o último parto.*

S1. Na última vez que a sra esteve grávida, a sra fez pré-natal? S001

1. Sim
2. Não

(Se S1=2, passe ao S44.)

S2. Na última vez que a sra esteve grávida a sra recebeu o cartão de pré-natal? S002

1. Sim
2. Não

(siga S3)

S3. Com quantas semanas de gravidez a sra iniciou o pré-natal? S003

Semanas

88. Não sabe

(siga S4)

S4. Quantas consultas de pré-natal a sra teve? S004

Consultas

(siga S5)

S5. Onde foi realizada a maioria das consultas do pré-natal? S005

- 1. Unidade básica de saúde (posto ou centro de saúde ou unidade de saúde da família)
- 2. Centro de Especialidades, Policlínica pública ou PAM – Posto de Assistência Médica
- 3. Hospital público/ambulatório
- 4. Consultório particular ou clínica privada
- 5. Ambulatório ou consultório de empresa ou sindicato
- 6. Outro

(siga S6)

S6. As consultas do pré-natal foram cobertas por algum plano de saúde? S006

- 1. Sim, todas
- 2. Sim, algumas
- 3. Não, nenhuma

(siga S7)

S7. A sra pagou algum valor pelas consultas do pré-natal? S007

*(**Entrevistador**: Se a entrevistada responder que pagou, mas teve reembolso, marque opção 2)*

- 1. Sim
- 2. Não

(siga S8)

S8. As consultas do pré-natal foram feitas através do Sistema Único de Saúde (SUS)?(Leia as opções de resposta) S008

- 1. Sim, todas
- 2. Sim, algumas
- 3. Não, nenhuma
- 4. Não sabe

(siga S9)

S9. Quem a atendeu na maioria das consultas?(Leia as opções de resposta) S009

- 1. Médico
- 2. Enfermeira
- 3. Técnico ou auxiliar de enfermagem
- 4. Parteira
- 5. Outro

(siga S10)

S10. Durante as consultas de pré-natal, a sra recebeu algum dos seguintes aconselhamentos?(Leia as opções de resposta)

a. Não faltar às consultas agendadas	S01001	1. Sim	2. Não	(siga S10b)
b. Manter uma alimentação saudável	S01002	1. Sim	2. Não	(siga S10c)
c. Não fumar	S01003	1. Sim	2. Não	(siga S10d)
d. Não beber	S01004	1. Sim	2. Não	(siga S10e)
e. Não fazer uso de tintura/alisamento de cabelo	S01005	1. Sim	2. Não	

(siga S11)

S11. Durante as consultas de pré-natal, a sra recebeu alguma destas orientações? (Leia as opções de resposta)

a. Sobre sinais de trabalho de parto	S01101	1. Sim	2. Não	(siga S11b)
b. Sobre sinais de risco na gravidez	S01102	1. Sim	2. Não	(siga S11c)
c. Sobre aleitamento materno	S01103	1. Sim	2. Não	(siga S12)

(siga S11)

S12. Durante o pré-natal a sra foi informada sobre a qual serviço de saúde a sra deveria ir no momento do parto? S012

- 1. Sim
- 2. Não

(siga S13)

S13. Mediram a sua altura na primeira consulta de pré-natal? S013

- 1. Sim
- 2. Não

(siga S14)

S14. Durante o pré-natal, em quantas consultas: (Leia as opções de resposta)

a. Mediram sua pressão arterial?	S01401	1. Todas	2. Algumas	3. Nenhuma	(siga S14b)
b. Mediram o seu peso?	S01402	1. Todas	2. Algumas	3. Nenhuma	(siga S14c)
c. Mediram a sua barriga? (fundo de útero)	S01403	1. Todas	2. Algumas	3. Nenhuma	(siga S14d)
d. Ouviram o coração do bebê?	S01404	1. Todas	2. Algumas	3. Nenhuma	(siga S14e)
e. Examinaram suas mamas?	S01405	1. Todas	2. Algumas	3. Nenhuma (siga S15)	

S15. Em alguma consulta do pré-natal o médico ou enfermeiro falou que sua pressão estava alta? S015

- 1. Sim
- 2. Não

(Se S15=2, passe ao S20.)

S16. O médico ou enfermeiro explicou sobre os riscos da pressão alta para a sra e para o bebê? S016

- 1. Sim
- 2. Não

(siga S17)

S17. A sra foi encaminhada para consulta com médico especialista por causa da pressão alta? S017

- 1. Sim
- 2. Não

(Se S17=2, passe ao S20.)

S18. A sra foi à consulta com o médico especialista? S018

- 1. Sim
- 2. Não

(Se S18=1, passe ao S20.)

S19. Qual o principal motivo da sra não ter ido à consulta com o especialista? S019

- 01. Não conseguiu marcar
- 02. Não achou necessário
- 03. Não sabia quem procurar ou aonde ir
- 04. Estava com dificuldades financeiras
- 05. O plano de saúde não cobria a consulta
- 06. O serviço de saúde era muito distante
- 07. O tempo de espera no serviço de saúde era muito grande
- 08. O horário de funcionamento do serviço de saúde era incompatível com as atividades de trabalho ou domésticas
- 09.Não havia especialista no serviço de saúde
- 10. Dificuldade de transporte
- 11. Outro

(siga S20)

S20. Durante o pré-natal, a sra fez exame de sangue? S020

- 1. Sim
- 2. Não

(Se S20=2, passe ao S33.)

S21. Em alguma consulta do pré-natal o médico ou enfermeiro falou que seu exame de sangue mostrou açúcar alto (presença de diabetes)? S021

- 1. Sim
- 2. Não

(Se S21=2, passe ao S27.)

S22. O médico ou enfermeiro explicou os riscos do açúcar alto no sangue para a sra e seu bebê? S022

- 1. Sim
- 2. Não

(siga S23)

S23. Explicaram sobre a alimentação que a sra deveria ter para ajudar a controlar o açúcar no sangue? S023

- 1. Sim
- 2. Não

(siga S24)

S24. A sra foi encaminhada para consulta com médico especialista por causa do diabetes? S024

1. Sim 2. Não

(S24=2, passe ao S27.)

S25. A sra foi à consulta com o médico especialista? S025

1. Sim 2. Não

(Se S25=1, passe ao S27.)

S26. Qual o principal motivo da sra não ter ido à consulta com o médico especialista? S026

01. A consulta está marcada, mas ainda não foi à consulta
02. Não conseguiu marcar
03. Não achou necessário
04. Não sabia quem procurar ou aonde ir
05. Estava com dificuldades financeiras
06. Teve dificuldades de transporte
07. O tempo de espera no serviço de saúde era muito grande
08. O plano de saúde não cobria a consulta
09.O serviço de saúde era muito distante
10. O horário de funcionamento do serviço de saúde era incompatível com as atividades de trabalho ou domésticas
11. Outro

(siga S27)

S27. Durante o atendimento pré-natal a sra realizou exame de sangue para sífilis? S027

1. Sim
2. Não
3. Não sabe

(Se S27=2 ou 3, passe ao S33.)

S28. A sra recebeu o resultado do exame para sífilis antes do parto? (leia as opções de resposta) S028

1. Sim, foi negativo
2. Sim, foi positivo
3. Não recebeu o resultado/ Não foi informada antes do parto

(Se S28=1 ou 3, passe ao S33.)

S29. A sra recebeu tratamento para sífilis?(Leia as opções de resposta) S029

1. Sim, antes do parto
2. Sim, depois do parto
3. Não

(siga S30)

S30. A sra foi orientada a usar preservativo? S030

1. Sim
2. Não

(siga S31)

S31. Foi pedido exame de sífilis para o seu parceiro? S031

1. Sim
2. Não

(siga S32)

S32. O seu parceiro foi tratado? S032

1. Sim
2. Não

(siga S33)

S33. Durante seu pré-natal, foi solicitado o teste para HIV? S033

1. Sim
2. Não
3. Não sabe

(Se S33 = 2 ou 3, passe ao S35.)

S34. A sra fez o teste de HIV? (Leia as opções de resposta) S034

1. Sim
2. Não, pois já sabia que estava infectada pelo HIV
3. Não

(siga S35)

S35. Durante o atendimento pré-natal a sra realizou exame de urina? S035

1. Sim
2. Não

(siga S36)

S36. Durante o pré-natal, quantos exames de ultrassonografia foram solicitados? S036

Exames

0. Nenhum

(Se S36 = 00, passe ao S42. Caso contrário, siga S37.)

S37. A sra conseguiu realizar os exames de ultrassonografia solicitados? (Leia as opções de resposta) S037

1. Sim, todos 2. Sim, alguns 3. Não, nenhum

(Se S37=1, passe ao S39)

S38. Qual o principal motivo da sra não ter conseguido fazer todos os exames de ultras-sonografia solicitados? S038

01. Não conseguiu marcar
02. Não achou necessário
03. Não sabia quem procurar ou aonde ir
04. Estava com dificuldades financeiras
05. Teve dificuldades de transporte
06. O serviço de saúde era muito distante
07. O tempo de espera no serviço de saúde era muito grande
08.O horário de funcionamento do serviço de saúde era incompatível com as atividades de trabalho ou domésticas
09.Não havia especialista no serviço de saúde para fazer o exame
10.Não havia equipamento disponível no serviço de saúde
11. O plano de saúde não cobria todos os exames
12. Outro

(Se S37 = 3, passe ao S42. Caso contrário, siga S39.)

S39. Os exames de ultrassonografia foram cobertos por algum plano de saúde? (Leia as opções de resposta) S039

1. Sim, todos
2. Sim, alguns
3. Não, nenhum

(siga S40)

S40. A sra pagou algum valor pelos exames de ultrassonografia?

*(**Entrevistador**: Se a entrevistada responder que pagou, mas teve reembolso total, marque a opção 2)*

S040

1. Sim
2. Não

(siga S41)

S41. Os exames de ultrassonografia foram feitos através do Sistema Único de Saúde (SUS)? (Leia as opções de resposta) S041

1. Sim, todos
2. Sim, alguns
3. Não, nenhum
4. Não sabe

(siga S42)

S42. Quanto tempo antes do parto foi a sua última consulta de pré-natal? (Leia as opções de resposta) S042

1. Menos de 7 dias
2. De 7 a 14 dias
3. De 15 a 30 dias
4. Mais de 30 dias
5. Não sabe, não lembra

(siga S43)

S43. Com quantas semanas de gravidez a sra estava na última consulta de pré-natal? S043

Semanas ☐☐ 88. Não sabe

(siga S44)

S44. Qual o seu peso antes de engravidar? S044

Quilograma ☐☐☐ 0. Não sabe

(siga S45)

S45. Quantos quilos a sra engordou na gestação?) S045

Quilograma ☐☐☐ 0. Não sabe 888. Não engordou

(siga S46)

Agora, vamos lhe fazer perguntas sobre a assistência ao último parto.

S46. Quem a atendeu no último parto?(Leia as opções de resposta) S046

1. Médico
2. Enfermeira
3. Parteira
4. Auxiliar de enfermagem
5. Estudantes de enfermagem ou medicina
6. Doula
7. Outra pessoa (parente, amigo, vizinho) sem treinamento
8. Ninguém

(siga S47)

S47. Onde foi realizado o seu último parto?(Leia as opções de resposta) S047

1. Hospital ou maternidade
2. Casa de parto
3. Outro tipo de serviço de saúde
4. Em casa
5. Outro

(Se S47=1 a 3, siga S48. Se S47=4 ou 5, passe ao S56.)

S48. O parto foi realizado no estabelecimento de saúde indicado no pré-natal? S048

1. Sim
2. Não
3. Não houve indicação

(siga S49)

S49. O parto foi realizado no primeiro estabelecimento de saúde que procurou? S049

1. Sim
2. Não

(Se S49=1, passe ao S51.)

S50. Quantos estabelecimentos de saúde a sra teve que ir até conseguir a internação para o parto? S050

☐☐ Estabelecimentos

(siga S51)

S51. O parto foi coberto por algum plano de saúde? S051

1. Sim
2. Não

(siga S52)

S52. A sra pagou algum valor pelo parto?
***(Entrevistador**: Se a entrevistada responder que pagou, mais teve reembolso total, marque a opção* S052

1. Sim
2. Não

(siga S53)

S53. O parto foi feito através do Sistema Único de Saúde (SUS)? S053

1. Sim
2. Não
3. Não sabe

(siga S54)

S54. O seu companheiro ou alguma pessoa da família, ou amiga ficou com a sra durante o trabalho de parto? S054

1. Sim
2. Não

(Se S54=1, passe ao S56.)

S55. Por que a sra não teve acompanhante durante o trabalho de parto? (Leia as opções de resposta) S055

1. Não sabia que podia
2. Não quis
3. Não deixaram
4. Não tinha quem a acompanhasse

(siga S56)

S56. O seu parto foi: (Leia as opções de resposta) S056

1. Vaginal
2. Cesáreo

(Se S56=1, passe ao S59.)

S57. A cesariana foi marcada com antecedência, durante o pré-natal? S057

1. Sim
2. Não

(siga S58)

S58. Qual o principal motivo da sra ter tido parto cesáreo? (Leia as opções de resposta) S058

1. Já tinha um parto cesáreo anterior
2. Queria ligar as trompas
3. Não queria sentir a dor do parto/Por ser mais conveniente
4. Por escolha do médico durante o pré-natal
5. Indicação médica por complicações na gravidez ou no trabalho de parto
6.Indicação médica porque não entrou em trabalho de parto
7. Outro

(Encerre o módulo. Passe ao Módulo U)

Módulo U. Saúde Bucal

Neste módulo, vamos fazer perguntas sobre a saúde bucal (dentes e gengivas) e assistência odontológica.

U1. Com que frequência o(a) sr(a) escova os dentes?(Leia as opções de resposta) U001

1. Nunca escovei os dentes
2. Não escovo todos os dias
3. 1 vez por dia
4. 2 vezes ou mais por dia
5. Não se aplica / Morador não tinha dente nenhum

(Se U1 = 1 ou 5, passe ao U5.)

U2. O que o(a) sr(a) usa para fazer a limpeza de sua boca? (Leia as opções de resposta)

a. Escova de dente?	U00201	1. Sim	2. Não	(siga U2b)
b. Pasta de dente?	U00202	1. Sim	2. Não	(siga U2c)
c. Fio dental?	U00203	1. Sim	2. Não	

(siga U4)

U4. Com que frequência o(a) sr(a) troca a sua escova de dente por uma nova?
(Leia as opções de resposta) U004

1. Com menos de 3 meses
2. Entre 3 meses e menos de 6 meses
3. Entre 6 meses e menos de 1 ano
4. Com mais de um ano
5. Nunca trocou

(siga U5)

U5. Em geral, como o(a) sr(a) avalia sua saúde bucal (dentes e gengivas)?
(Leia as opções de resposta) U005

1. Muito Boa
2. Boa
3. Regular
4. Ruim
5. Muito ruim

(siga U6)

U6. Que grau de dificuldade o(a) sr(a) tem para se alimentar por causa de problemas com seus dentes ou dentadura?(Leia as opções de resposta) U006

1. Nenhum
2. Leve
3. Regular
4. Intenso
5. Muito intenso

(Se J13=1, siga U9.
Se J13≠1, passe ao U23.)

U9. Qual o principal motivo da sua última consulta ao dentista?
(Leia as opções de resposta) U009

01. Limpeza, revisão, manutenção ou prevenção
02. Dor de dente
03. Extração
04. Tratamento dentário
05. Problema na gengiva
06. Tratamento de ferida na boca
07. Implante dentário
08. Aparelho nos dentes (ortodôntico)
09. Colocação/manutenção de prótese ou dentadura
10. Fazer radiografia
11. Fazer o orçamento do tratamento
12. Outro (

(siga U10)

U10. Onde foi a última consulta odontológica? U010

01. Unidade básica de saúde (posto ou centro de saúde ou unidade de saúde da família)
02. Centro de Especialidades, Policlínica pública ou PAM – Posto de Assistência Médica
03. UPA (Unidade de Pronto Atendimento)
04. CEO – Centro de Especialidades Odontológicas
05. Outro tipo de Pronto Atendimento Público (24 horas)
06. Pronto-socorro ou emergência de hospital público
07. Hospital público/ambulatório
08. Consultório particular ou clínica privada
09. Ambulatório ou consultório de empresa ou sindicato
10. Pronto-atendimento ou emergência de hospital privado
11. Outro

(siga U11)

U11. O local onde o(a) sr(a) teve atendimento odontológico fica:
(Leia as opções de resposta) U011

1. Na mesma cidade que o(a) sr(a) mora
2. Em outra cidade

(siga U14)

U14. Como o(a) sr(a) conseguiu a consulta odontológica? U014
(Leia as opções de resposta)

1. Foi direto ao serviço de saúde, sem marcar consulta
2. Agendou a consulta previamente
3. Foi encaminhado(a) ou ajudado(a) por equipe de saúde da família
4. Foi encaminhado(a) ou ajudado(a) pela Unidade Básica de Saúde
5. Foi encaminhado(a) por outro serviço ou profissional de saúde
6. Outro

(Se U14 = 1, 3, 4, 5 ou 6, passe ao U17. Se U14=2, siga U15.)

U15. Como foi feito o agendamento? (Leia as opções de resposta) U015

1. Deixou agendado em consulta anterior
2. Por meio de visita à unidade de saúde para marcação de consulta
3. Por telefone
4. Agendamento virtual, pela internet
5. Outro

(siga U17.)

U17. Qual o tempo total que o(a) sr(a) ficou em fila de espera desde a hora que chegou ao serviço de saúde até conseguir o atendimento com dentista?

U01701 U01702

Preencher com as horas e/ou minutos que ficou esperando em fila de espera desde a hora que chegou no serviço de saúde até conseguir o atendimento.

Horas Minutos

(siga U18)

U18. Quanto tempo durou a consulta odontológica?

U01801 U01802

Preencher com as horas e/ou minutos que durou a consulta odontológica.

Horas Minutos

(siga U19)

U19. A consulta odontológica foi coberta por algum plano de saúde?
(***Entrevistador***: *Se o(a) entrevistado (a) responder que pagou, mas teve reembolso total, marque a opção 2)*

U019

1. Sim
2. Não

(siga U20)

U20. O(A) sr(a) pagou algum valor pela consulta odontológica? U020

1. Sim
2. Não

(siga U21)

U21. A consulta odontológica foi feita pelo SUS? U021

1. Sim
2. Não
3.Não sabe

(siga U22)

U22. De forma geral, como o(a) sr(a) avalia o atendimento recebido? (Leia as opções de resposta) U022

1. Muito bom
2. Bom
3.Regular
4.Ruim
5.Muito ruim

(siga U23)

U23. Lembrando-se dos seus dentes de cima, o(a) sr(a) perdeu algum dente?

1. Não
2. Sim, perdi U02301 Dentes
3. Sim, perdi todos os dentes de cima U023

(siga U24)

U24. Lembrando-se dos seus dentes de baixo, o(a) sr(a) perdeu algum dente?

1. Não
2. Sim, perdi U02401 Dentes
3. Sim, perdi todos os dentes de baixo U024

Se AMBAS as respostas dos quesitos U23 e U24 forem = 1, passe ao Módulo X. Caso contrário, siga U25.

U25. O(A) sr(a) usa algum tipo de prótese dentária (dente artificial)?(Leia as opções de resposta) U025

1. Não
2. Sim, para substituir um dente
3. Sim, para substituir mais de um dente
4. Sim, prótese dentária total (dentadura/chapa) em cima
5. Sim, prótese dentária total (dentadura/chapa) em baixo
6. Sim, próteses dentárias totais (dentaduras/chapas) em cima e em baixo

(Encerre o módulo. Passe ao Módulo X.)

Módulo X. Atendimento médico

Neste módulo, vamos fazer perguntas sobre o atendimento médico, acesso ao atendimento e sua avaliação sobre o atendimento recebido no serviço de saúde.

X1. Quando foi a última vez que o(a) sr(a) consultou um médico? (Leia as opções de resposta) X001

1. Há menos de 2 semanas
2. Entre 15 dias e um mês
3. Entre um mês e 3 meses atrás
4. Entre três meses e um ano
5. Há mais de um ano
6. Nunca foi ao médico

(Se X1= 6, passe ao X25.)

X2. Por qual motivo o(a) sr(a) precisou consultar um médico?(Leia as opções de resposta) X002

1. Acidente ou lesão
2. Continuação de tratamento ou terapia
3. Consulta pré-natal
4. Exame médico periódico
5. Outro exame médico (admissional, para carteira de motorista, etc.)
6. Problema de saúde mental
7. Doença ou outro problema de saúde
8. Outro

(siga X3)

X3. Onde procurou o primeiro atendimento médico por este motivo? X003

01. Unidade básica de saúde (posto ou centro de saúde ou unidade de saúde da família)
02. Centro de Especialidades, Policlínica pública ou PAM – Posto de Assistência Médica
03. CAPS – Centro de Atenção Psicossocial
04. UPA (Unidade de Pronto Atendimento)
05. Outro tipo de Pronto Atendimento Público (24 horas)
06. Pronto-socorro ou emergência de hospital público
07. Hospital público/ambulatório
08. Consultório particular ou clínica privada
09. Ambulatório ou consultório de empresa ou sindicato
10. Pronto-atendimento ou emergência de hospital privado
11. No domicílio, com médico particular
12. No domicílio, com médico da equipe de saúde da família
13. Outro

(siga X4)

X4. Na primeira vez que procurou atendimento médico por este motivo o(a) sr(a conseguiu ser atendido? X004

1. Sim
2. Não

(Se X4 = 2, siga X5.)
(Se X4 = 1 e X3 ≠ 11 ou 12, passe ao X8.)
(Se X4 = 1 e X3 = 11 ou 12, passe ao X15.)

X5. Quantas vezes voltou a procurar atendimento médico por este motivo? (Leia as opções de resposta)

X005 Vezes

0. Nenhuma / desistiu

(Se X5 = 00, passe ao X24. Caso contrário, siga X6.)

X6. O(A) sr(a) conseguiu o atendimento médico que precisava?(Leia as opções de resposta) X006

1. Sim
2. Não, mas continua tentando
3. Não, desistiu

(Se X6=1, siga X7.
Se X6=2, passe ao X25. Se X6=3, passe ao X24.)

X7. Onde conseguiu o atendimento médico por este motivo? X007

- 01. Unidade básica de saúde (posto ou centro de saúde ou unidade de saúde da família)
- 02. Centro de Especialidades, Policlínica pública ou PAM – Posto de Assistência Médica
- 03. UPA (Unidade de Pronto Atendimento)
- 04. CAPS – Centro de Atenção Psicossocial
- 05. Outro tipo de Pronto Atendimento Público (24 horas)
- 06. Pronto-socorro ou emergência de hospital público
- 07. Hospital público/ambulatório
- 08. Consultório particular ou clínica privada
- 09. Ambulatório ou consultório de empresa ou sindicato
- 10. Pronto-atendimento ou emergência de hospital privado
- 11. No domicílio, com médico particular
- 12. No domicílio, com médico da equipe de saúde da família
- 13. Outro

(Se X7 = 01 ao 10 ou 13, siga X8. Se X7 = 11 ou 12, passe ao X15.)

X8. Onde fica o serviço de saúde em que o(a) sr(a) teve a consulta médica? X008

1. Na mesma cidade que o(a) sr(a) mora
2. Em outra cidade

(siga X11)

X11. Como o(a) sr(a) conseguiu a consulta médica?(Leia as opções de resposta) X011

1. Foi direto ao serviço de saúde, sem marcar consulta
2. Agendou a consulta previamente
3. Foi encaminhado(a) ou ajudado(a) por equipe de saúde da família
4. Foi encaminhado(a) ou ajudado(a) por Unidade Básica de Saúde
5. Foi encaminhado(a) por outro serviço ou profissional de saúde
6. Exame periódico pago ou encaminhado pelo empregador
7. Atendimento de emergência
8. Outro

(Se X11=2 ou 8, siga X12. Se X11=1, 3, 4, 5, 6 ou 7, passe ao X14.)

X12. Como foi feito o agendamento? X012
(Leia as opções de resposta)

1. Deixou agendado em consulta anterior
2. Por meio de visita à unidade de saúde para marcação de consulta
3. Por telefone
4. Agendamento virtual, pela internet
5. Outra forma

(siga X14)

X14. Qual o tempo total que o(a) sr(a) ficou em fila de espera desde a hora que chegou ao serviço de saúde até conseguir o atendimento com médico?

X01401 X01402

Horas Minutos

Preencher com as horas e/ou minutos que ficou esperando em fila de espera desde a hora que chegou no serviço de saúde até conseguir o atendimento.

(siga X15)

X15. Quanto tempo durou a consulta médica?

X01501 X01502

Horas Minutos

Preencher com as horas e/ou minutos que durou a consulta médica..

(siga X16)

X16. Que tipo de médico o/a atendeu? (Leia as opções de resposta) X016

1. Médico da família ou generalista
2. Clínico geral
3. Ginecologista
4. Médico especialista (cardiologista, nefrologista, oftalmologista, dermatologista, urologista, oncologista, otorrinolaringologista, etc.)
5. Outra forma

(siga X17)

X17. A consulta médica foi coberta por plano de saúde? X017

1. Sim
2. Não

(siga X18)

X18. O(A) sr(a) pagou algum valor pela consulta médica? X018

*(**Entrevistador**: Se o(a) entrevistado(a) responder que pagou, mas teve reembolso total, marque opção 2)*

1. Sim
2. Não

(siga X19)

X19. A consulta médica foi feita pelo SUS? X019

1. Sim
2. Não
3. Não sabe

(Se X3 = 11 ou 12, passe ao X22. Se X3 ≠ 11 ou 12, siga X20.)

(Se X7 = 11 ou 12, passe ao X22. Se X7 ≠ 11 ou 12, siga X20.)

X20. De um modo geral, como o(a) sr(a) avalia o atendimento recebido quanto: (Leia as opções de resposta)

a. À disponibilidade de equipamentos necessários para a consulta médica? X02001	1. Muito bom	2. Bom	3. Regular	4. Ruim	5. Muito ruim	(siga X20b)
b. Ao espaço disponível para a consulta médica? X02002	1. Muito bom	2. Bom	3. Regular	4. Ruim	5. Muito ruim	(siga X20c)
c. Ao tempo gasto com deslocamento? X02003	1. Muito bom	2. Bom	3. Regular	4. Ruim	5. Muito ruim	(siga X20d)
d. Ao tempo de espera até ser atendido? X02004	1. Muito bom	2. Bom	3. Regular	4. Ruim	5. Muito ruim	(siga X20e)
e. À forma como os atendentes o/a receberam? X02005	1. Muito bom	2. Bom	3. Regular	4. Ruim	5. Muito ruim	(siga X20f)
f. À limpeza das instalações, incluindo os banheiros? X02006	1. Muito bom	2. Bom	3. Regular	4. Ruim	5. Muito ruim	(siga X22)

X22. De um modo geral, como o(a) sr(a) avalia o atendimento recebido quanto:(leia as opções de resposta)

a. Às habilidades do médico para tratá-lo (a)?	X02201	1. Muito bom	2. Bom	3. Regular	4. Ruim	5. Muito ruim	(siga X22b)
b. Ao respeito do médico na maneira de atendê-lo(a)?	X02202	1. Muito bom	2. Bom	3. Regular	4. Ruim	5. Muito ruim	(siga X22c)
c. À clareza nas explicações do médico?	X02203	1. Muito bom	2. Bom	3. Regular	4. Ruim	5. Muito ruim	(siga X22d)
d. À disponibilidade de tempo para fazer perguntas sobre o seu problema ou tratamento?	X02204	1. Muito bom	2. Bom	3. Regular	4. Ruim	5. Muito ruim	(siga X22e)
e. À possibilidade de falar em privacidade com o médico?	X02205	1. Muito bom	2. Bom	3. Regular	4. Ruim	5. Muito ruim	(siga X22f)
f. À liberdade em escolher o médico?	X02206	1. Muito bom	2. Bom	3. Regular	4. Ruim	5. Muito ruim	(siga X25)

X24. Qual o principal motivo do(a) sr(a) ter desistido de procurar atendimento médico? X024

01. Não houve mais necessidade, pois melhorou
02. Procurou diretamente a farmácia
03. Não teve mais tempo ou disponibilidade para procurar atendimento
04. Falta de dinheiro ou dificuldades financeiras
05. Não conseguiu marcar a consulta ou pegar senha
06. Nas vezes que procurou, não tinha médico atendendo
07. Nas vezes que procurou, esperou muito e desistiu
08. Nas vezes que procurou o serviço de saúde não estava funcionando
09. Não podia pagar pela consulta
10. O plano de saúde não cobria a consulta
11. Outro

(siga X25)

X25. O(A) sr(a) já se sentiu discriminado (a) ou tratado(a) pior do que as outras pessoas no serviço de saúde, por algum médico ou outro profissional de saúde por um desses motivos?(Leia as opções de resposta)

a. Falta de dinheiro	X02501	1. Sim	2. Não	(siga X25b)
b. Classe social	X02502	1. Sim	2. Não	(siga X25c)
c. Raça/cor	X02503	1. Sim	2. Não	(siga X25d)
d. Tipo de ocupação	X02504	1. Sim	2. Não	(siga X25e)
e. Tipo de doença	X02505	1. Sim	2. Não	(siga X25f)
f. Preferência sexual	X02506	1. Sim	2. Não	(siga X25g)
g. Religião/crença	X02507	1. Sim	2. Não	(siga X25h)
h. Sexo	X02508	1. Sim	2. Não	(siga X25i)
i. Idade	X02509	1. Sim	2. Não	(siga X25j)
j. Outro	X02510	1. Sim	2. Não	

(encerre a entrevista)

OBSERVAÇÕES

Módulo W - Informações Laboratoriais

W00101 - Informe o seu peso (primeira pesagem em kg) ....

W00102 - Informe o seu peso (segunda pesagem em kg) ....

W00201 - Informe a sua altura (primeira medida em cm)...........

W00202 - Informe a sua altura (segunda medida em cm) ....

W00301 - Informe a sua circunferência da cintura (primeira medida em cm)...........

W00302 - Informe a sua circunferência da cintura (segunda medida em cm)...........

W00401 - Informe a pressão arterial sistólica (primeira medida)...........

W00402 - Informe a pressão arterial diastólica (primeira medida...........

W00403 - Informe a pressão arterial sistólica (segunda medida) ....

W00404 - Informe a pressão arterial diastólica (segunda medida)...........

W00405 - Informe a pressão arterial sistólica (terceira medida)...........

W0i0406 - Informe a pressão arterial diastólica (terceira medida)...........

Muito obrigado pela sua participação! As informações que o(a) sr(a) nos forneceu serão valiosas para a formulação de políticas para a melhoria da assistência á saúde no Brasil.

(Encerre a entrevista)
