# Supplementary material for: Reasons for nonadherence to vaccination for influenza among older people in Brazil
Source: PLoS One. 2021 Nov 8;16(11):e0259640. doi: 10.1371/journal.pone.0259640 (PMC8575254; doi:10.1371/journal.pone.0259640)
Supplement: S2 File — (PDF) [file pone.0259640.s002.pdf]

Analysis Scripts

Descriptive Analysis

```
. svy linearized : proportion vacina
. svy linearized : proportion motivos
. svy linearized : mean idade
. svy linearized : proportion sexo
. svy linearized : proportion vacina
```

Expanding the sample data to the total Brazilian population

```
. svy linearized, subpop(if vacina==2) : total vacina
```

Table 1

```
. svy linearized : tabulate região vacina, row ci obs pearson
. svy linearized : tabulate sexo vacina, row ci obs pearson
. svy linearized : tabulate faixas vacina, row ci obs pearson
. svy linearized : tabulate raca vacina, row ci obs pearson
. svy linearized : tabulate conjuge vacina, row ci obs pearson
. svy linearized : tabulate escolaridade2 vacina, row ci obs pearson
. svy linearized : tabulate sabe_ler vacina, row ci obs pearson
. svy linearized : tabulate plano_saude vacina, row ci obs pearson
```

Table 2

```
. svy linearized : tabulate região motivos, row ci obs
. svy linearized : tabulate sexo motivos, row ci obs
. svy linearized : tabulate faixas motivos, row ci obs
. svy linearized : tabulate raca motivos, row ci obs
. svy linearized : tabulate conjuge motivos, row ci obs
. svy linearized : tabulate escolaridade2 motivos, row ci obs
. svy linearized : tabulate sabe_ler motivos, row ci obs
. svy linearized : tabulate plano_saude motivos, row ci obs
```

Fig. 1

```
. svy linearized : tabulate faixas motivos, row ci obs
```
